# Frontotemporal dementia associated *CHCHD10*^*V57E*^ mutation aggravates tau pathology via disrupting the CHCHD10-Rab7A-TBC1D15 complex

**DOI:** 10.1038/s41419-026-08983-9

**Published:** 2026-06-19

**Authors:** Jie Sheng, Jing Yan, Yan Xu, Xingyu Xu, Xuemei Zhang, Anqi Wang, Buhui Liu, Jun Ma, Xiuying Duan, Heng Zhou, Wei Liu

**Affiliations:** 1 Jiangsu Key Laboratory of Brain Disease Bioinformation, Xuzhou Medical University, Xuzhou, China; 2 Department of Biochemistry, School of Basic Medical Sciences, Xuzhou Medical University, Xuzhou, China; 3 The Affiliated Hospital of Xuzhou Medical University, Xuzhou, China; 4 Jiangsu Province Key Laboratory of Anesthesiology, Jiangsu Province Key Laboratory of Anesthesia and Analgesia Application Technology, NMPA Key Laboratory for Research and Evaluation of Narcotic and Psychotropic Drugs, Xuzhou Medical University, Xuzhou, China; 5 School of Life Sciences, Shandong First Medical University & Shandong Academy of Medical Sciences, Jinan, China

**Keywords:** Cell biology, Diseases

## Abstract

Frontotemporal dementia (FTD), a neurodegenerative disorder characterized by early-onset cognitive decline, includes two major pathologic types: FTD-Tau and FTD-TDP. *Coiled-coil-helix-coiled-coil-helix domain containing 10* (CHCHD10) encodes a mitochondrial protein, and the CHCHD10^V57E^ variant is a novel mutation clinically identified in FTD patients. The role of CHCHD10 mutants in the pathogenesis of FTD-TDP has been largely reported. However, whether CHCHD10 variants impact the FTD-tau pathology or tau-mediated neurodegeneration is not clear. Here, we generated *CHCHD10*^*V57E*^ mutant *Drosophila* and mammalian cell culture models on a human R406W tau or 0N4R tau expressing background. We discovered that the *CHCHD10*^*V57E*^ mutation aggravates retinal degeneration, climbing defects, and short lifespan in tau^R406W^ *Drosophila*. Additionally, the *CHCHD10*^*V57E*^ mutation promotes synaptic integrity defects, increases secretion of total and phosphorylated tau, and induces cytotoxicity in tau-overexpressing mammalian cell culture models. Further studies indicated that localization of the endogenous and wild-type CHCHD10, as well as the V57E mutant, includes the mitochondrial inner and outer membranes. Furthermore, we showed that phosphorylated tau accumulates within the endo-lysosomal system of the *CHCHD10*^*V57E*^-expressing flies and mammalian cells. Depletion of endosomal protein Rab7A partially attenuates neurodegeneration, reduces total and phosphorylated tau secretion, and diminishes cytotoxicity in tauopathy models expressing *CHCHD10*^*V57E*^. Mechanistically, we presented evidence that the CHCHD10-Rab7A-TBC1D15 complex maintains Rab7A in an inactive state. By contrast, CHCHD10^V57E^ expression dissociates the protein complex, resulting in Rab7A activation. Taken together, our study proposes a model whereby the FTD-associated CHCHD10^V57E^ mutation modulates the activation process of Rab7A, resulting in promoting the development of tau pathologies.

## Introduction

Frontotemporal dementia (FTD), a major cause of young-onset dementia, is characterized by progressive neuronal atrophy in the frontal and/or temporal lobes of the brain [1, 2]. Based on the presence of abnormal tau, TDP-43, or FUS proteins within cellular inclusions in the brain, FTD is subdivided into FTD-Tau, FTD-TDP, and FTD-FUS [3]. It has been reported that FTD-Tau and FTD-TDP constitute nearly 95% of FTD cases [4, 5]. The FTD pathology can also be confirmed by identifying specific genetic mutations. For example, mutations in the *microtubule-associated protein tau* (MAPT) gene are sufficient to cause FTD-Tau [6]. Tau mutations also drive other tauopathies related neurodegenerative diseases, such as Alzheimer’s disease (AD) [7]. Molecular mechanism studies have revealed that pathological tau may induce neurotoxicity due to its loss of function, toxic gain of function, or mediate amyloid-β-induced toxicity [7, 8]. In addition, pathological tau can also be released into the extracellular space, thereby enhancing tau pathology and spread [9, 10]. Thus, the detection of tau from cerebrospinal fluid (CSF) or blood samples has been widely used to discriminate FTD-tau from FTD-TDP or AD [11, 12]. However, the mechanisms underlying tau secretion remain incompletely understood, although several pathways have been proposed: direct plasma membrane translocation, exosomal export, secretory endo-lysosomes, and cell-to-cell transfer [11, 13].

An increasing number of genetic factors have been implicated in the tau aggregation and secretion process. For example, *APOE4*—a common genetic risk factor for AD and FTD—worsens neurodegeneration in a setting of tauopathy by regulating tau aggregation and secretion [14, 15]. *The VCP*^*D395G*^ mutation impairs its ATPase and tau disaggregase activity, thereby increasing tau aggregation [16]. In addition, the AD genetic risk factor *FRMD4A* regulates tau secretion by activating a pathway linked to presynaptic vesicle machinery and polarity signaling [17]. Although numerous genetic risk factors for FTD and other tauopathies have been identified, understanding how these factors influence tau pathology remains limited.

*Coiled-coil-helix–coiled-coil-helix 10* (*CHCHD10*) encodes a subunit protein of the mitochondrial contact site and cristae organizing system (MICOS) complex [18]. The role of *CHCHD10* mutants in the pathogenesis of FTD-TDP has been largely reported [19–23]. The most well-studied example is *CHCHD10*^*S59L*^, which has been recognized as a cause of FTD-ALS [21]. *CHCHD10*^*S59L*^ expression can lead to cell toxicity and mitochondrial defects through mitochondrial translocation of TDP-43 in the nervous system of model organisms, including worms, fruit flies, and mice [21–23]. *CHCHD10*^*R15L*^ expression induced similar mitochondrial defects as *CHCHD10*^*S59L*^ does, and was accompanied by cytoplasmic TDP-43 aggregates [20]. The *CHCHD10*^*G66V*^ mutation is associated with ALS, spinal muscular atrophy (SMAJ), and Charcot-Marie-Tooth type 2 (CMT2) [24]. Its ectopic expression leads to the accumulation of phosphorylated TDP-43 [24]. However, whether *CHCHD10* mutations influence the tau pathology or tau-mediated neurodegeneration is not understood.

*CHCHD10*^*V57E*^ is a novel mutation clinically identified in FTD patients and is associated with severe behavioral changes and cognitive defects [25]. While the *CHCHD10*^*V57E*^ mutation is reported to elevate mitochondrial superoxide levels and impair mitochondrial respiration [26], its pathogenic consequences for FTD pathogenesis remain unclear. Here, we demonstrate that *CHCHD10*^*V57E*^ expression exacerbates tauopathies in FTD-Tau *Drosophila* models and tau-overexpressing mammalian cell culture models. In addition, we showed that phosphorylated tau accumulates within the endo-lysosomal system of the *CHCHD10*^*V57E*^-expressing flies and mammalian cells. Depletion of endo-lysosomal protein Rab7A partially attenuated retinal neurodegeneration, reduced total and phosphorylated tau secretion, and diminished cell toxicity in tauopathy models expressing *CHCHD10*^*V57E*^. Mechanistically, we presented evidence that the CHCHD10-Rab7A-TBC1D15 complex maintains Rab7A in an inactive state. By contrast, CHCHD10^V57E^ expression dissociates the protein complex, resulting in Rab7A activation. Taken together, our study suggests that the CHCHD10^V57E^ affects the Rab7A activation states to regulate endo-lysosome-related tau secretion and tau-mediated neurodegeneration.

## Results

### CHCHD10^V57E^ exacerbates tau^R406W^-induced retinal degeneration, climbing defects, and short lifespan in *Drosophila*

**Fig. 1:**
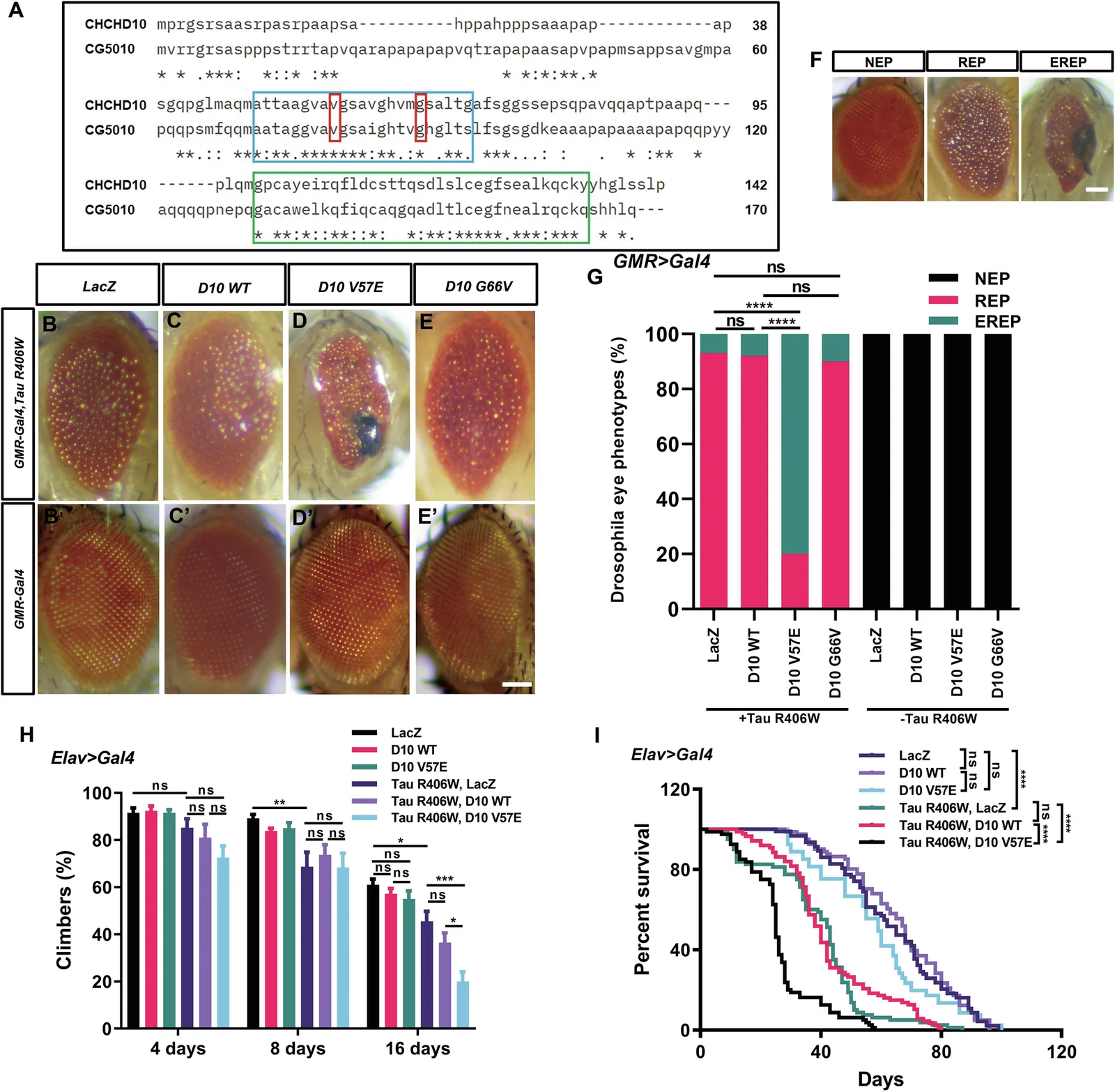
CHCHD10^V57E^ aggravates Tau^R406W^-induced neurodegeneration in *Drosophila.* **A** Alignment of human CHCHD10 and *Drosophila* CG5010 amino acid sequences reveals 44% identity and 54% similarity. Conserved regions include the hydrophobic helix domain (highlighted in blue) and the CHCH domain (highlighted in green). Notably, Val57 and Gly66 in human *CHCHD10* are conserved as Val79 and Gly88 in *Drosophila*, respectively, and both residues are located within the hydrophobic helix domain. **B**–**E**, **B’**–**E’** Eye morphology of *Drosophila* with indicated genotypes imaged by stereoscopic microscopes (Scale bar: 100 μm). **F** Classification of eye phenotypes: normal (NEP), rough (REP), and enhanced rough (EREP) (Scale bar: 100 μm). **G** Quantification of the eye phenotypes from flies with the indicated genotypes (Chi-square test, *n* = 50, ****p < 0.0001; ns, non-significant). **H** The climbing ability of flies of indicated genotypes is shown (one-way ANOVA with Bonferroni’s multiple comparisons test, 6 groups of flies, 10 flies in each group). **I** The lifespan of flies of indicated genotypes is shown (Log-rank test, 3–5 groups of flies, 20 flies in each group).

Human *CHCHD10* shares an orthologous gene with *Drosophila melanogaster*, which is *CG5010* [27]. It is noticed that disease-associated Val57 and Gly66 in human CHCHD10 are conserved as Val79 and Gly88 in *Drosophila* (Fig. 1A). To develop *Drosophila* models for *CHCHD10* mutant-induced human diseases, we generated transgenic flies carrying wild-type, *V57E*, and *G66V* mutant forms of human *CHCHD10* under the control of *u*pstream *a*ctivating *s*equence (UAS). *Tau*^*R406W*^ missense mutation results in frontotemporal lobar degeneration with an Alzheimer’s disease-like phenotype [28, 29]. Misexpression of tau^R406W^ in the fly retina caused a marked “rough eye” phenotype, characterized by reduced eye size and missing bristles [30]. To investigate how the *CHCHD10* mutations impact tau pathology, we then crossed various combinations of *lacZ* (control), *CHCHD10*^*WT*^, *CHCHD10*^*V57E*^, *CHCHD10*^*G66V*^, and *Tau*^*R406W*^ transgenes with the eye-specific *GMR-Gal4* driver. All adult progenies were collected for analysis of the rough eye phenotype (Fig. 1B–G). Without tau^R406W^ expression, all the progenies with various genotypes showed intact eye morphology noted as “normal eye phenotype, NEP” (Fig. 1B’–E’, F, G). As expected, tau^R406W^ expression in the fly eyes leads to the obvious “rough eye phenotype, REP” (Fig. 1B, F, G). Compared with the control animals (*UAS-lacZ*), expression of the human *CHCHD10*^*WT*^ in the *GMR*>*UAS-tau*^*R406W*^ flies showed no significant difference in eye size (Fig. 1B, C, F, G), indicating that overexpression of wild-type human *CHCHD10* in *Drosophila* is non-toxic. Surprisingly, when CHCHD10^V57E^ and tau^R406W^ were co-expressed, the number of “enhanced rough eye phenotype, EREP” with smaller eye size and black spots of apoptotic tissue was significantly increased compared to the control animals (Fig. 1B–D, F, G). Besides the light microscope analysis, we also visualized the eye phenotypes using a scanning electron microscope (SEM) (Fig. S1). Notably, *CHCHD10*^*V57E*^ expression in the *GMR*>*UAS-tau*^*R406W*^ flies induced severe eye defects with loss of ommatidial bristles and fusion of ommatidia (Fig. S1A–C), indicating that overexpression of human *CHCHD10*^*V57E*^ accelerates the retinal degeneration phenotypes of FTD-Tau *Drosophila* models.

Both the V57E and G66V mutations localize to the conserved hydrophobic helix region of the CHCHD10 protein, and this helix domain is highly conserved across species (Fig. 1A). Their consistent location within the same key functional/structural region of CHCHD10 makes G66V a structurally matched comparator. Previous studies have reported that the *CHCHD10*^*G66V*^ mutant induces the accumulation of phosphorylated TDP-43 [24]. To test whether *CHCHD10*^*V57E*^ specifically exacerbates tau^R406W^ toxicity, we generated *CHCHD10*^*G66V*^-expressing transgenic flies. In contrast to *CHCHD10*^*V57E*^, expression of *CHCHD10*^*G66V*^ in the *GMR*>*UAS-tau*^*R406W*^ fly showed moderate eye morphology phenotypes with around ~10% of EREP, which are comparable with the genotype of *GMR*>*UAS-CHCHD10*^*WT*^, *UAS-tau*^*R406W*^ (Fig. 1C, E–G). Therefore, it is highly suggested that the *CHCHD10*^*V57E*^ mutant might have special dominant effects that enhance the tau^R406W^ toxicity.

Neuronal degeneration is often associated with climbing defects and shortened lifespan in *Drosophila*. Thus, we overexpressed wild-type and *V57E* mutant forms of *CHCHD10* in neurons by crossing with a pan-neuronal driver *elav-Gal4*. Male progenies were collected for both climbing ability and lifespan analysis. Compared with the control animals (*elav*>*UAS-LacZ*), the *elav*>*CHCHD10*^*WT*^ and *elav*>*CHCHD10*^*V57E*^ animals displayed comparable climbing ability and lifespan in both young age and old age (Fig. 1H, I). These data indicated that expression of *CHCHD10*^*V57E*^ in neurons has little negative effect on the climbing and survival of flies. To analyze the role of *CHCHD10* on the climbing ability and lifespan of *tau*^*R406W*^-expressed flies, *tau*^*R406W*^ was separately co-expressed with *LacZ*, *CHCHD10*^*WT*^, and *CHCHD10*^*V57E*^. Consistent with previous reports [31], the expression of *tau*^*R406W*^ results in aging-associated climbing defects and shortened lifespan (Fig. 1H, I). Notably, we found that the expression of *CHCHD10*^*V57E*^ shortened lifespan and exacerbated climbing defects of the old age (16 days old) tau^R406W^-expressed flies, whereas the expression of *CHCHD10*^*WT*^ did not have such effects (Fig. 1H, I). Thus, we conclude that *CHCHD10*^*V57E*^ exacerbates the neurodegeneration phenotypes generated with human *tau*^*R406W*^ in *Drosophila*.

### CHCHD10^V57E^ exacerbates tau pathology in mammalian cell culture models

**Fig. 2:**
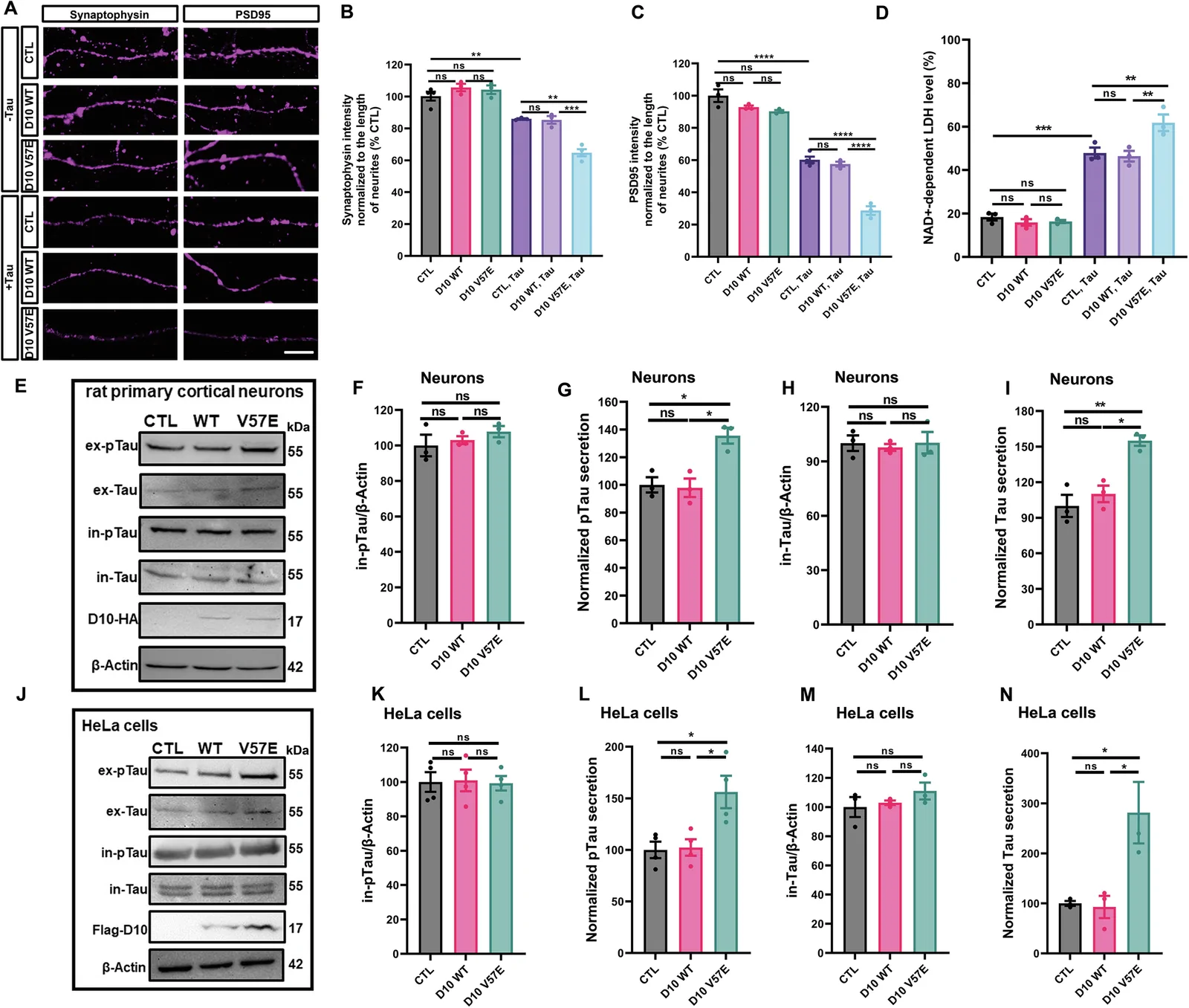
CHCHD10^V57E^ exacerbates tau pathology in mammalian cell culture models. **A** Rat primary cortical neurons were co-transduced with lentivirus expressing CHCHD10^WT^-HA, CHCHD10^V57E^-HA, or control, together with the tau-V5 or its respective control lentivirus. Synaptophysin and PSD95 antibody delineate the presynaptic area and the postsynaptic area (scale bar: 20 μm). **B**, **C** Quantification of synaptophysin and PSD95 signal intensities in (**A**) (one-way ANOVA with Bonferroni’s multiple comparisons test, *n* = 3–4, **p < 0.01; ***p < 0.001; ****p < 0.001; ns non-significant). **D** HeLa cells or stably transfected HeLa cells carrying the tau-V5 construct were transfected with *Flag-CHCHD10*^*WT*^ expression construct, *Flag-CHCHD10*^*V57E*^ expression construct, or empty vector. Cell culture media were subjected to NAD^+^-dependent D-lactate dehydrogenase detection (one-way ANOVA with Bonferroni’s multiple comparisons test, *n* = 3, **p < 0.01; ***p < 0.001; ns, non-significant). **E** Rat primary cortical neurons transduced with CHCHD10^WT^-HA, CHCHD10^V57E^-HA, or control lentivirus, together with the tau-V5 lentivirus. Cell culture media were subjected to anti-V5 immunoprecipitation. Subsequently, the immune-purified anti-V5 samples were analyzed by immunoblot using antibodies against phosphorylated tau (pTau) and total tau, which represent extracellular pTau and total tau levels (ex-pTau and ex-Tau), respectively. Cell lysates were subject to immunoblot using anti-pTau and total tau, which represent intracellular pTau and total tau levels (in-pTau and in-Tau), respectively. β-Actin serves as a loading control. **F** Quantification of intracellular pTau immunoblot in (**E**). Intracellular pTau levels normalized to β-Actin (one-way ANOVA with Bonferroni’s multiple comparisons test, *n* = 3, ns, non-significant). **G** Quantification of extracellular pTau immunoblot in (**E**). Extracellular pTau levels calculated as (extracellular pTau)/(normalized intracellular pTau) (one-way ANOVA with Bonferroni’s multiple comparisons test, *n* = 3, *p < 0.05; ns, non-significant). **H** Quantification of intracellular total tau immunoblot in (**E**). Intracellular total tau (in-Tau) levels normalized to β-Actin (one-way ANOVA with Bonferroni’s multiple comparisons test, *n* = 3, ns, non-significant). **I** Quantification of extracellular total tau immunoblot in (**E**). Extracellular total Tau levels calculated as (extracellular total Tau)/(normalized intracellular total tau) (one-way ANOVA with Bonferroni’s multiple comparisons test, *n* = 3, *p < 0.05; **p < 0.01; ns, non-significant). **J** Stably transfected HeLa cells carrying the *tau-V5* construct were transfected with *Flag-CHCHD10*^*WT*^ expression construct, *Flag-CHCHD10*^*V57E*^ expression construct, or empty vector. Extracellular and intracellular phosphorylated tau levels were analyzed by immunoblot using an antibody against phosphorylated tau. β-Actin serves as a loading control. **K** Quantification of intracellular pTau immunoblot in (**J**) (one-way ANOVA with Bonferroni’s multiple comparisons test, *n* = 4, ns, non-significant). **L** Quantification of extracellular pTau immunoblot in (**J**) (one-way ANOVA with Bonferroni’s multiple comparisons test, *n* = 4, *p < 0.05; ns, non-significant). **M** Quantification of intracellular total tau immunoblot in (**J**) (one-way ANOVA with Bonferroni’s multiple comparisons test, *n* = 3, ns, non-significant). **N** Quantification of extracellular total tau immunoblot in (**J**) (one-way ANOVA with Bonferroni’s multiple comparisons test, *n* = 3, *p < 0.05; ns non-significant).

Rat primary neurons and HeLa cells are often used to investigate tau pathology in vitro [32, 33]. To explore whether the *CHCHD10*^*V57E*^ mutant enhances tau pathology in mammalian cells, the human 0N4R *tau* isoform was separately co-expressed with *CHCHD10*^*WT*^ or *CHCHD10*^*V57E*^ in rat cortical primary neurons by using lentivirus. The presynaptic marker (synaptophysin) and the postsynaptic marker (PSD95) are frequently utilized to reflect the axonal integrity in the neurodegenerative neuronal cell cultured models [34, 35]. As previously reported [33, 36, 37], overexpression of tau in the primary neurons reduces the intensity of synaptophysin and PSD95 (Fig. 2A–C). In addition, we found that simultaneous overexpression of CHCHD10^V57E^ and tau in the neurons leads to a significant reduction of the two synaptic markers, compared to tau co-expressed with the control (empty vector) or CHCHD10^WT^ (Fig. 2A–C). These data demonstrated that *CHCHD10*^*V57E*^ worsens the synaptic integrity defects induced by tau toxicity in rat cortical primary neurons.

To evaluate if CHCHD10^V57E^ leads to cellular toxicity, the extracellular level of the enzyme lactate dehydrogenase (LDH) was examined. LDH exists in the cytoplasm of cells and is released to the extracellular space when the plasma membrane is damaged [38]. Compared with the control, the *CHCHD10*^*WT*^ and *CHCHD10*^*V57E*^-expressing cells displayed comparable LDH levels (Fig. 2D), indicating that expression of CHCHD10^WT^ or CHCHD10^V57E^ had minimal impact on cell viability. Notably, co-expression of *CHCHD10*^*V57E*^ and *tau* induced significantly greater cell toxicity than co-expression of tau with either empty vector or *CHCHD10*^*WT*^ (Fig. 2D). Hence, we conclude that *CHCHD10*^*V57E*^ expression exacerbates tau-induced synaptic integrity defects and increases the cytotoxicity in cultured tau-overexpression rat primary neurons and HeLa cells.

### CHCHD10^V57E^ promotes total and phosphorylated tau secretion in mammalian cell culture models

Tau hyperphosphorylation is strongly associated with the initiation and progression of tauopathies [39]. We further detected the expression levels of total and phosphorylated tau in the rat cortical primary neurons co-expressing tau and CHCHD10 variants. The results showed no significant changes in either total tau or pS199/202-tau levels among the control cells, *CHCHD10*^*WT*^-expressing cells, and *CHCHD10*^*V57E*^-expressing cells (Fig. 2E, F, H). Since total and phosphorylated tau may also be secreted into the extracellular milieu in cell culture and animal models of neurodegenerative diseases [40], the cell culture medium from rat cortical primary neurons was collected for the immunoblot test. Interestingly, we found that the expression of CHCHD10^V57E^ in the neurons resulted in a significant ~1.4-fold increase in extracellular pS199/202-tau levels (Fig. 2E, G) and a ~1.5-fold increase in extracellular total tau (Fig. 2E, I) compared to levels in control and CHCHD10^WT^-expressing neurons.

We replicated our investigations by using HeLa cells. The stably expressing human ON4R tau HeLa cells were transfected with the *CHCHD10*^*WT*^ expression construct, the *CHCHD10*^*V57E*^ expression construct, or the empty vector. Consistent with our findings in primary neurons, the immunoblot analysis revealed that compared with controls, *CHCHD10*^*V57E*^-expressing HeLa cells showed a ~1.5-fold increase in extracellular pS199/202-tau levels (Fig. 2J, L) and a ~3-fold increase in extracellular total tau levels (Fig. 2J, N), reflecting enhanced secretion of total and phosphorylated tau. Furthermore, neither intracellular total tau nor pS199/202-tau differed significantly between the control cells, *CHCHD10*^*WT*^-expressing cells, and *CHCHD10*^*V57E*^-expressing cells (Fig. 2J, K, M). Thus, we speculate that CHCHD10^V57E^ stimulates the secretion of total tau and pS199/202-tau in tau-overexpressing rat cortical primary neurons and HeLa cell culture models.

In neurodegenerative diseases, misfolded proteins can be secreted from cells via exosomes. However, some studies have shown that the secretion of misfolded proteins occurs in a vesicle-free form [41]. To analyze whether tau and p-tau are secreted via exosomes in the cell culture models of co-expressing tau and CHCHD10 variants, we collected cell culture media and isolated exosomes. We did not detect the total tau and pS199/202-tau protein in the collected extracellular exosome vesicles (Fig. S2), suggesting that tau and pTau undergo direct secretion to the extracellular space rather than vesicle-mediated release. Altogether, we conclude that *CHCHD10*^*V57E*^ expression promotes total and phosphorylated tau secretion in cultured tau-overexpression rat primary neurons and HeLa cells.

### CHCHD10^V57E^ leads to moderate mitochondrial defects

**Fig. 3:**
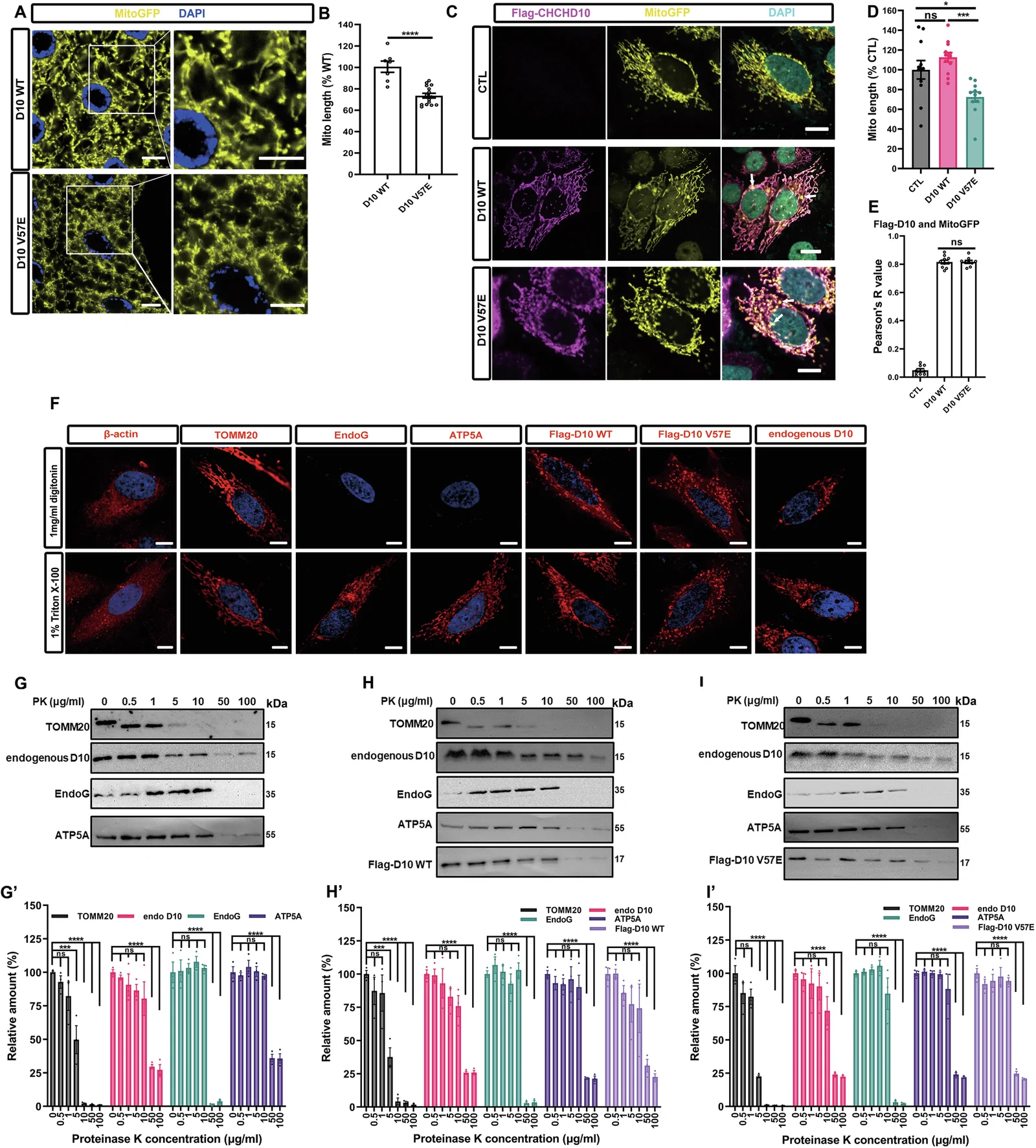
CHCHD10^V57E^ overexpression results in mitochondrial fragmentation, and CHCHD10 protein localizes on the mitochondrial inner and outer membranes. **A** Mitochondrial morphology analysis in *Drosophila* fat body cells expressing CHCHD10 variants and mitochondrial marker (MitoGFP) driven by *cg-Gal4*. Confocal images show mitochondrial structure (left panels: overview; right panels: higher-magnification views of boxed regions). Scale bars: 10 μm. **B** Quantification of mitochondrial length in *Drosophila* fat body cells (Student’s *t*-test, *n* = 7–15, ****p < 0.0001). **C** HeLa cells were transfected with *Flag-CHCHD10*^*WT*^ expression construct, *Flag-CHCHD10*^*V57E*^ expression construct, or empty vector, together with MitoGFP construct. White arrowheads indicate mitochondria that are MitoGFP-positive but the Flag-CHCHD10-negative. Scale bars: 10 μm. Immunofluorescence images in (**A** and **C**) were presented in pseudocolors to enhance accessibility. **D** Mitochondrial length quantification of HeLa cells is presented as mean ± SEM (one-way ANOVA with Bonferroni’s multiple comparisons test, *n* = 11–12, *p < 0.05; ***p < 0.001; ns, not significant). **E** Pearson’s correlation between Flag-CHCHD10 and MitoGFP (mean ± SEM, Student’s *t*-test, *n* = 9–10, ns, non-significant). **F** HeLa cells were fixed and treated with 1 mg/mL digitonin or 1% Triton X-100, followed by staining with the indicated antibodies (red). **G**–**I** HeLa cells were transfected with empty vector, *Flag-CHCHD10*^*WT*^ expression construct, or *Flag-CHCHD10*^*V57E*^ expression construct. Mitochondria were isolated and treated with protease K at the indicated concentrations, followed by western blot analysis with the indicated antibodies. **G’**–**I’** Bar charts show the digestion trend of proteins of interest by Proteinase K with the indicated protease concentrations in (**G**–**I**). The residual protein levels in each proteinase K‑treated group were normalized to the untreated control (0 μg/mL proteinase K), and results are presented as the relative amount of remaining protein (mean ± SEM, one‑way ANOVA with Bonferroni’s multiple comparisons, *n* = 3, ***p < 0.001; ****p < 0.0001; ns non-significant).

Healthy mitochondria are pivotal to normal neuronal function and integrity. To determine whether *CHCHD10*^*V57E*^ induces mitochondrial defects, we analyzed mitochondrial functions in *Drosophila* fat body tissue, HeLa cells, and rat primary cortical neurons expressing the *CHCHD10*^*V57E*^ mutation. *The Drosophila* fat body, a monolayer of large polyploid cells, constitutes a powerful in vivo model system for cell biological analysis of organelle architecture and function [42]. First, we crossed *CHCHD10*^*WT*^ or *CHCHD10*^*V57E*^ transgenic flies with a fat body tissue-related driver, *cg-Gal4*, together with the mitochondrial marker MitoGFP. In flies expressing *CHCHD10*^*V57E*^, early third *instar* larvae fat body tissues exhibited fragmented mitochondria, with the average mitochondrial length mildly reduced compared to the *CHCHD10*^*WT*^ control tissues (Fig. 3A, B). Next, we expressed *Flag-CHCHD10*^*WT*^ or *Flag-CHCHD10*^*V57E*^ in HeLa cells as well. The immunocytochemistry assay confirmed that both Flag-CHCHD10^WT^ and Flag-CHCHD10^V57E^ co-localize with MitoGFP, and cells expressing *Flag-CHCHD10*^*V57E*^ showed mitochondrial fragmentation (Fig. 3C–E). In addition, lentiviruses encoding Flag-CHCHD10^WT^ and Flag-CHCHD10^V57E^ were used to transduce rat cortical primary neurons, and mitochondria were labeled with anti-TOMM20 antibodies. The result showed that Flag-CHCHD10^V57E^ expression led to fragmented mitochondria compared to control and *CHCHD10*^*WT*^*-*expressing neurons (Fig. S3A, B). These data indicated that *CHCHD10*^*V57E*^ results in defects in mitochondrial morphology in flies and mammalian cells.

We also measured ATP production, ROS, and mitochondrial membrane potential in cells expressing *CHCHD10*^*V57E*^. In the flies, HeLa cells, and rat primary cortical neurons, *CHCHD10*^*V57E*^ expression does not impact ATP production (Fig. S3C–E). In contrast, *CHCHD10*^*WT*^ expression in flies slightly increases ATP production (Fig. S3C), which is consistent with previous studies [23]. To measure ROS production, we stained both the *CHCHD10*^*WT*^-expressing cells, *CHCHD10*^*V57E*^-expressing cells, and the control cells with fluorescent dye, 2’,7’-dichlorodihydrofluorescein (DCF). In the fat body cells, either expression of *CHCHD10*^*WT*^ or *CHCHD10*^*V57E*^ shows comparable levels of DCF fluorescence intensity to the control (Fig. S3F, G). However, DCF fluorescence intensity in *CHCHD10*^*V57E*^ expressing HeLa cells increased ~4-fold, and a ~3-fold increase was observed in *CHCHD10*^*V57E*^ expressing neurons compared with controls (Fig. S3H–J). These results indicated that HeLa cells and neurons may be more sensitive to *CHCHD10*^*V57E*^ expression, resulting in increased ROS production. To monitor mitochondrial membrane potential, we stained the *CHCHD10*^*WT*^-expressing cells, *CHCHD10*^*V57E*^-expressing cells, and the control cells with fluorescent dye, tetramethylrhodamine ethyl ester (TMRE). Either in flies, HeLa cells, or rat primary cortical neurons, the TMRE signals in CHCHD10^V57E^-expressing cells are comparable to those in controls (Fig. S3K–O). Taken together, we propose that *CHCHD10*^*V57E*^-expression causes moderate mitochondrial defects.

### Localization of wild-type CHCHD10 and the V57E mutant includes the mitochondrial outer membrane

Previous studies have shown that specific CHCHD10 mutants alter their mitochondrial location [43]. Our data show that, similar to the wild-type protein, Flag-CHCHD10^V57E^ exhibited typical mitochondrial staining and colocalized prominently with the mitochondrial marker protein MitoGFP in HeLa cells (Fig. 3C). The MitoGFP fusion protein incorporates the Cox8 mitochondrial targeting sequence fused to GFP, enabling specific localization to the mitochondrial inner membrane (IM) [44]. We note that some points of MitoGFP are not perfectly colocalized with either Flag-CHCHD10^WT^ or Flag-CHCHD10^V57E^ (Fig. 3C, E). Then, we further analyzed the sub-organelle localization of endogenous and ectopically expressed CHCHD10. HeLa cells were divided into two groups: untransfected cells and cells transfected with Flag-tagged CHCHD10 variants. Following fixation, both groups were permeabilized with either 1 mg/mL digitonin or 1% Triton X-100. Untransfected HeLa cells were then immunostained with specific antibodies targeting β-actin, TOMM20, EndoG, or ATP5A, while transfected cells were probed with an anti-Flag antibody. Digitonin at 1 mg/mL could only permeabilize the plasma membrane, but not the mitochondrial membrane, whereas 1% Triton X-100 could permeabilize both the plasma and mitochondrial membranes [45]. The cytosolic proteins, such as β-actin and the mitochondrial outer membrane (OM) proteins, such as TOMM20, were labeled in the cells treated with digitonin (Fig. 3F). The proteins localized in the mitochondrial intermembrane space (IMS), on the inner membrane (IM), and in the matrix could only be stained in the Triton X-100-treated cells (Fig. 3F). Surprisingly, we found that Flag-CHCHD10^WT^ and Flag-CHCHD10^V57E^ were detectable in both digitonin and Triton X-100-treated cells, indicating that ectopically expressed CHCHD10^WT^ and CHCHD10^V57E^ proteins localized to the mitochondrial outer membrane (Fig. 3F).

To verify whether this outer membrane localization was an artifact of CHCHD10 overexpression, we performed immunofluorescence staining with an endogenous CHCHD10 antibody. The result showed that the localization of endogenous CHCHD10 also includes the mitochondrial outer membrane (Fig. 3F). To further confirm this result, we then isolated the mitochondria from three HeLa cell lines: native (endogenous CHCHD10), Flag-CHCHD10^WT^ expressing stable, and Flag-CHCHD10^V57E^ expressing stable cells. The isolated mitochondria were subjected to proteinase K (PK) digestion at different concentrations, followed by immunoblotting analysis of various mitochondrial proteins localized in different mitochondrial sub-compartments. The OM proteins, such as TOMM20, are more accessible to PK digestion than proteins localized in the intermembrane space (e.g., EndoG), or the inner membrane (e.g., ATP5A) [45]. As expected, the mitochondrial outer membrane protein TOMM20 was the first to be completely degraded by proteinase K, with barely detectable signal observed at 10 μg/mL PK (Fig. 3G–I’). When PK concentration increased to 50 μg/mL, the mitochondrial intermembrane space protein EndoG was nearly completely degraded (Fig. 3G–I’). Consistent with the inner membrane marker ATP5A, Flag-CHCHD10^WT^, Flag-CHCHD10^V57E^, and endogenous CHCHD10 displayed similar PK digestion kinetics: these proteins were significantly reduced at 50 μg/mL PK, and ~25% of the residual proteins remained after treatment with 100 μg/mL PK (Fig. 3G–I’). These results indicated a hypothesis of CHCHD10 sequential digestion kinetics: the OM-localized CHCHD10 undergoes PK digestion first, followed by the IM-localized counterpart. Taken together, our results suggest that the Flag-CHCHD10^WT^, Flag-CHCHD10^V57E^, and endogenous CHCHD10 proteins localize in the mitochondrial inner membrane and outer membrane, at least in HeLa cells.

### Overexpression of CHCHD10^V57E^ alters endo-lysosomal organization, but does not change the distribution of autophagy markers

**Fig. 4:**
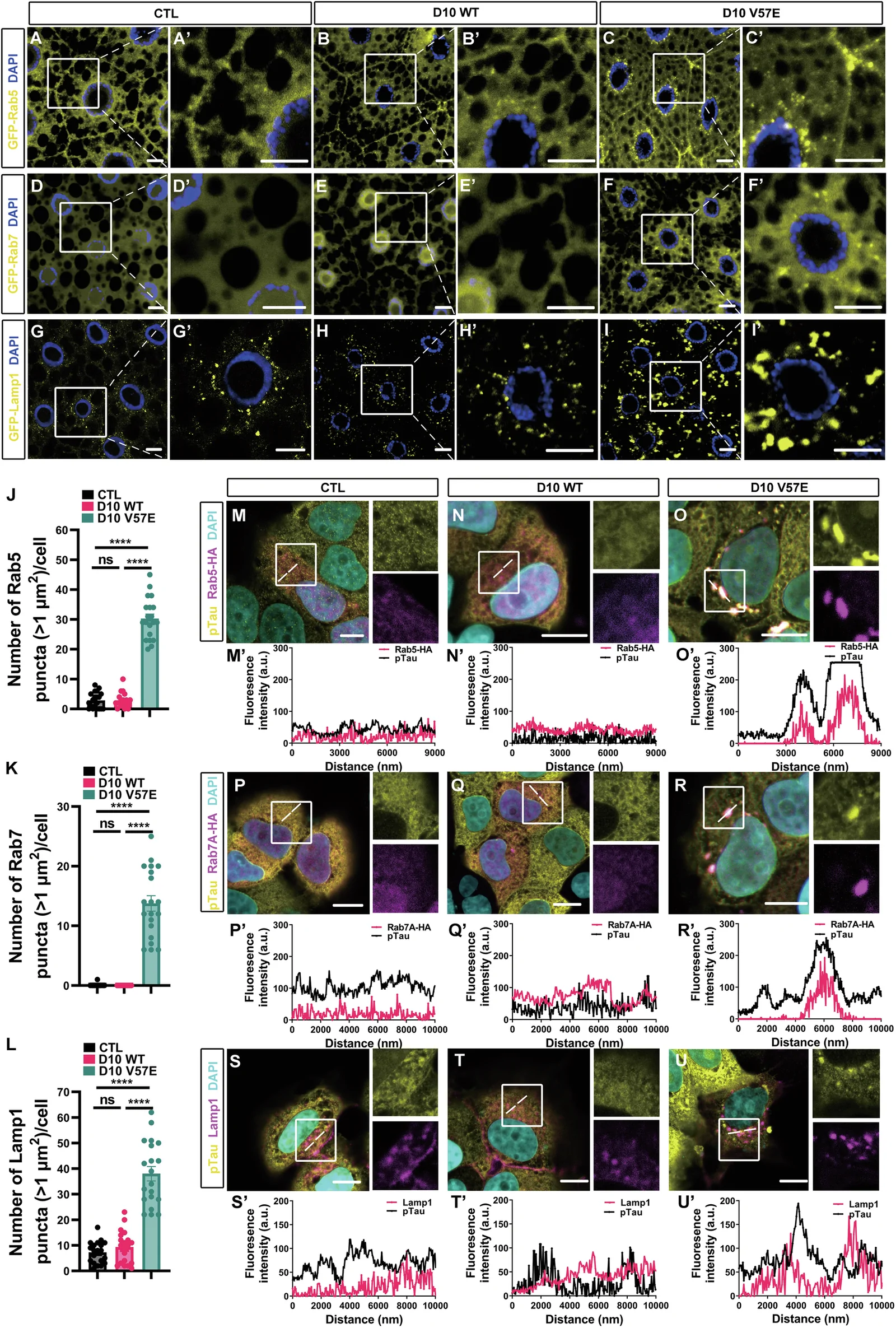
CHCHD10^V57E^ promotes phosphorylated tau trafficking to the endo-lysosomal system. **A**–**I** Flies carrying the fat body-related *cg-Gal4* driver were crossed to *UAS-LacZ*, *UAS-CHCHD10*^*WT*^*-HA* or *UAS-CHCHD10*^*V57E*^*-HA* flies together with various endo-lysosomal marker transgenes. Animals were dissected at the stage of third-*instar* larvae. The pattern of **A**–**C** GFP-Rab5, **D**–**F** GFP-Rab7, and **G**–**I** GFP-Lamp1 was examined by a laser confocal microscope. **A’**–**I’** Magnified views of boxed regions in (**A**–**I**). Scale bars: 10 μm. **J**–**L** Quantification of the number of indicated marker puncta (>1 µm²) in *LacZ*, *CHCHD10*^*WT*^ and *CHCHD10*^*V57E*^ expressing fat body tissues (one-way ANOVA with Bonferroni’s multiple comparisons test, *n* = 20, ****p < 0.0001, ns not significant). **M**–**U** Stably transfected HeLa cells carrying the *tau-V5* construct were transfected with *Flag-CHCHD10*^*WT*^ expression construct, *Flag-CHCHD10*^*V57E*^ expression construct, or empty vector, together with Rab5-HA construct or Rab7A-HA construct. Anti-HA antibody stained Rab5-HA/Rab7A-HA (violet); Anti-Lamp1 antibody stained Lamp1 (violet); Anti-phospho-Tau (Ser199, Ser202) antibody stained phosphorylated tau (yellow). **M’**–**U’** The colocalization curves of yellow and violet signals of the positions marked by short lines in the boxed region in (**M**–**U**). Immunofluorescence images in (**A**–**I**, **A’**–**I’**, and **M**–**U**) were presented in pseudocolors to enhance accessibility.

Mitochondria, as the cellular powerhouses and metabolic hubs, can communicate with the endo-lysosomal compartment within the cell to maintain cellular homeostasis [46]. Since endosome-lysosomes play a crucial role in tau trafficking, degradation, and secretion [41, 47], we explored whether the overexpression of *CHCHD10*^*V57E*^ affects the distribution of the endo-lysosomal reporters. Rab5 localizes to early endosomal compartments, and the transition from Rab5 to Rab7 is a hallmark of endosomal maturation [48]. This process requires Rab7 activation by its specific guanine nucleotide exchange factor, which arrives on endosomes together with Rab7 and is accompanied by a sharp transition from Rab5 to Rab7 [49]. It is reasonable that there is a period during which both Rab5 and Rab7 proteins co-exist on the same endosomes [50]. Rab7 and Lamp1 are the two proteins commonly used to define late endosomes and lysosomes, respectively [48]. When late endosomes fuse with lysosomes, there is a period before Rab7 proteins are fully cleared from the lysosomes after the fusion event [51]. Accordingly, the majority of endo-lysosome vesicles are positive for both Rab7 and Lamp1, and vesicles positive for Lamp1 alone or both Rab7 and Lamp1 represent terminal compartments in the endo-lysosomal pathway [51]. Although Rab5, Rab7, and Lamp1 are not strictly exclusive to discrete endo-lysosomal maturation stages, these markers are widely used to label the endo-lysosomal system. The *UAS-CHCHD10*^*WT*^ and *UAS-CHCHD10*^*V57E*^ transgenic flies were crossed to *cg-Gal4* driver flies and combined with those endo-lysosomal reporter transgenic lines. The results demonstrated that compared to cells expressing *CHCHD10*^*WT*^, *CHCHD10*^*V57E*^ expression induced dramatic accumulation of GFP-Rab5 (Fig. 4A–C’, J), GFP-Rab7 (Fig. 4D–F’, K), and GFP-Lamp1 (Fig. 4G–I’, L) in the cytoplasm, indicating that CHCHD10^V57^ alters the endo-lysosomal organization.

Given that overexpression of *CHCHD10*^*V57E*^ causes moderate mitochondrial dysfunction (Figs. 3A–E, S3), we further investigated whether this could activate the autophagy pathway, which functions to clear the macromolecules, proteins, and damaged organelles through the endo-lysosomal system [52]. We analyzed the autophagy markers in the fat body tissues of *CHCHD10*^*WT*^-expressed and *CHCHD10*^*V57E*^-expressed flies. The mCherry-Atg8a, GFP-Atg8a, GFP-Atg5, autophagy flux marker GFPRFP-Atg8a, and mitophagy-specific marker Mito-QC showed diffused cytoplasmic distribution in both *CHCHD10*^*WT*^-expressed and *CHCHD10*^*V57E*^-expressed cells (Fig. S4A–J). In addition, the fully formed autophagosome marker GFP-Syx17 and the lysosome marker Cp1/cathepsin L were co-stained to assess the autophagosome–lysosome fusion process in *CHCHD10*^*WT*^-expressed cells and *CHCHD10*^*V57E*^-expressed cells. In *CHCHD10*^*WT*^-expressed cells, GFP-Syx17 was largely diffuse, and Cp1/cathepsin L signals were weak (Fig. S4K). In *CHCHD10*^*V57E*^-expressed cells, GFP-Syx17 showed a diffuse pattern, and Cp1/cathepsin L formed several large puncta (Fig. S4L). The colocalization analysis of GFP-Syx17 and Cp1/cathepsin L indicates no significant differences between the *CHCHD10*^*WT*^-expressed and *CHCHD10*^*V57E*^*-*expressed cells (Fig. S4F’).

Next, we assessed autophagy by immunostaining using an antibody against endogenous LC3 (the Atg8a homolog in mammals), and detected LC3 puncta accumulation in the control (empty vector), *CHCHD10*^*WT*^-expressing, and *CHCHD10*^*V57E*^-expressing HeLa cells. Although a small number of LC3 puncta were detected in *CHCHD10*^*V57E*^-expressing HeLa cells (Fig. S4O), no significant difference was observed relative to the control groups (Fig. S4M–O, G’). Altogether, we conclude that expression of CHCHD10^V57E^ mainly alters endo-lysosomal organization, but does not change the distribution of autophagy markers.

### Overexpression of CHCHD10^V57E^ promotes phosphorylated tau trafficking to the endo-lysosomal system

To test whether *CHCHD10*^*V57E*^ could promote tau trafficking to the endo-lysosomal system, we co-expressed Tau-V5 and Rab5-HA in HeLa cells stably expressing Flag-CHCHD10^WT^, Flag-CHCHD10^V57E^, or a control Flag tag only. Subsequently, we performed immunofluorescence analysis using anti-phospho-tau (Ser199, Ser202) and anti-HA antibodies to detect the phosphorylated tau (pTau) distribution pattern. Indeed, in the presence of Flag-CHCHD10^V57E^, phosphorylated tau protein showed obvious colocalization with Rab5 puncta (Fig. 4O, O’). By contrast, no significant colocalization between pTau and Rab5 was observed in the Flag-CHCHD10^WT^ or control groups (Fig. 4M–N’). Similar to Rab5, we noticed that Rab7A or Lamp1 either completely or partially colocalized with cytoplasmic pTau puncta in HeLa cells expressing Flag-CHCHD10^V57E^ (Fig. 4P–U’). In addition, the colocalization between pTau and Rab7 was also confirmed in *Drosophila*. We found that the pTau proteins were positive for the endosome marker GFP-Rab7 in tau^R406W^ and CHCHD10^V57E^ co-expressed fat body tissues (Fig. S5A, B). In most cases, cytoplasmic tau aggregates can be engulfed by autophagy and delivered to endo-lysosomes [53]. Even though the distribution of autophagy reporters shows no major differences between the cells expressing CHCHD10^V57E^ alone and cells expressing CHCHD10^WT^ alone (Fig. S4A–O, A’–G’), we found that tau^R406W^ overexpression unbiasedly induces pTau colocalizing with the autophagy marker mCherry-Atg8a in the control group, *CHCHD10*^*WT*^-expressing, and *CHCHD10*^*V57E*^-expressing flies (Fig. S4P). Hence, the pTau-induced autophagy process may still help pTau trafficking to the endo-lysosomal system in the *CHCHD10*^*V57E*^-expressed cells. Taken together, our data suggest that overexpression of CHCHD10^V57E^ promotes phosphorylated tau trafficking and accumulation in the endo-lysosomal system.

### Overexpression of CHCHD10^V57E^ promotes tau pathology in a Rab7A-dependent manner

**Fig. 5:**
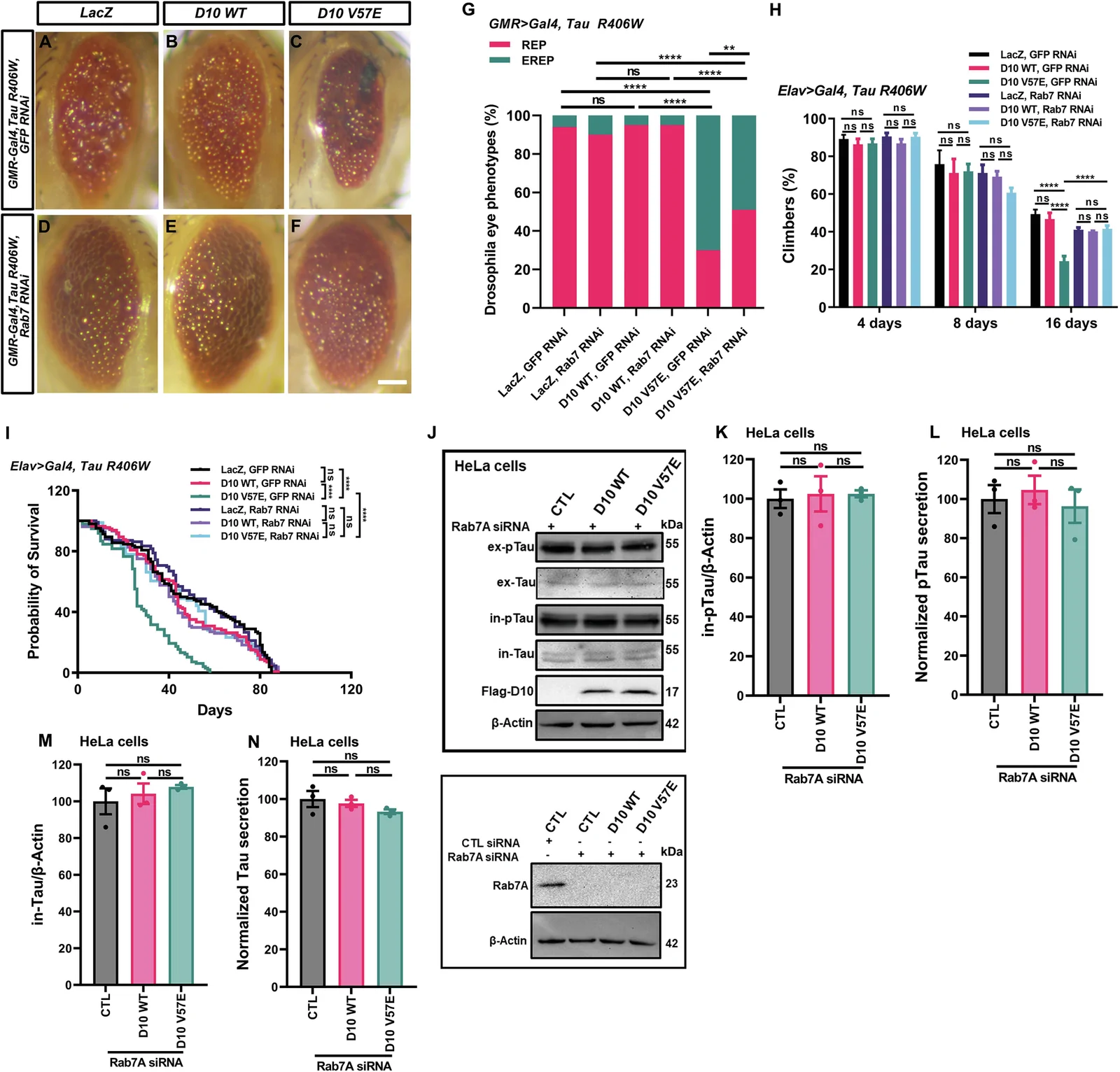
CHCHD10^V57E^ promotes tau pathology in a Rab7A-dependent manner. **A**–**F** *Drosophila* eyes of indicated genotypes imaged by stereoscopic microscopy. Scale bars: 100 μm. **G** Quantification of the eye phenotypes from flies with the indicated genotypes (Chi-square test, *n* = 50, **p < 0.01; ****p < 0.0001; ns, non-significant). **H** The climbing ability analysis of *Drosophila* with indicated genotypes (one-way ANOVA with Bonferroni’s multiple comparisons test, 6 groups of flies, 10 flies in each group). **I** Survival curves of Drosophila with specified genotypes (Log-rank test, 5 groups of flies, 20 flies in each group). **J** Stably transfected HeLa cells carrying the *tau-V5* construct were transfected with *Rab7A* siRNA to facilitate *Rab7A* knockdown, followed by transfection with various CHCHD10 expression plasmids. Immunoblot analysis of intracellular and extracellular protein fractions. Immunoblot confirmation of Rab7A knockdown (lower panel). β-Actin serves as a loading control. **K**, **L** Quantitative analysis of intracellular and extracellular pTau levels (one-way ANOVA with Bonferroni’s multiple comparisons test, *n* = 3, ns, not significant). **M**, **N** Quantitative analysis of intracellular and extracellular total tau levels (one-way ANOVA with Bonferroni’s multiple comparisons test, *n* = 3, ns not significant).

The endo-lysosome protein Rab7A belongs to the Ras-like small GTPase superfamily and plays multiple roles in vesicle-mediated endocytic processes and lysosome degradation [54]. Previous studies have shown that the constitutively active and dominant negative forms of Rab7A increased and decreased tau secretion, respectively [55]. Rab7A overexpression induced tau aggregation, whereas Rab7A knockdown decreased it [56]. Given that CHCHD10^V57E^ induces phosphorylated tau colocalization with Rab7A and enhances phosphorylated tau secretion (Figs. 2E, J, 4R, R’), we hypothesized that CHCHD10^V57E^ may regulate pTau secretion via Rab7A. To test this hypothesis, we first crossed various combinations of *CHCHD10*^*WT*^, *CHCHD10*^*V57E*^, *lacZ* (control), *Tau*^*R406W*^, and *Rab7* RNAi or *GFP* RNAi transgenes with flies carrying the eye-specific *GMR-Gal4* driver. We observed an approximately 60% reduction in Rab7 mRNA levels in flies’ eyes (Fig. S6A). The heads of adult flies were collected for eye phenotype analysis. We found that in the Rab7-depletion background, the retinal degeneration in *GMR*>*CHCHD10*^*V57E*^*; Tau*^*R406W*^ flies was partially rescued, accompanied by significantly reduced incidence of the EREP subgroup (Fig. 5A–G). Furthermore, the same set of transgenic flies was crossed with the neuronal *elav-Gal4* driver. The results showed that the aging-associated climbing defects and short lifespan phenotypes elicited in *elav*>*CHCHD10*^*V57E*^*; Tau*^*R406W*^ flies were rescued following Rab7 depletion (Fig. 5H, I).

To investigate Rab7A’s role in HeLa cells co-expressing tau and CHCHD10^V57E^, stably transfected HeLa cells carrying the tau-V5 construct were transfected with *Rab7A*-targeting siRNA or non-targeting control siRNA, followed by transfection with Flag-tagged CHCHD10 (WT, V57E mutant, or empty vector control). RT-qPCR confirmed that efficient reduction of *Rab7A* mRNA levels by siRNA knockdown (Fig. S6B). Notably, knocking down Rab7A in cells co-expressing tau and CHCHD10^V57E^ decreased extracellular LDH levels (Fig. S6C), reflecting that CHCHD10^V57E^-enhanced tau-induced cell toxicity relies on Rab7A. In addition, lysates from cells expressing either an *empty vector* (control), *CHCHD10*^*WT*^, or *CHCHD10*^*V57E*^ were analyzed by immunoblot following Rab7A depletion. Knocking down Rab7A resulted in comparable levels of the secreted total and phospho-tau across the control, *CHCHD10*^*WT*^-expressing, and *CHCHD10*^*V57E*^-expressing cells (Fig. 5J–N), indicating that CHCHD10^V57E^-enhanced total and phospho-tau secretion depends on Rab7A. Taken together, these data demonstrate that Rab7A functions as a downstream effector of CHCHD10^V57E^, contributing to neurodegeneration, cell toxicity, and the secretion of total and phosphorylated tau in FTD-Tau *Drosophila* and tau-overexpressing cell culture models.

### The V57E mutation abrogates low-affinity or transient interaction of CHCHD10 with Rab7A

**Fig. 6:**
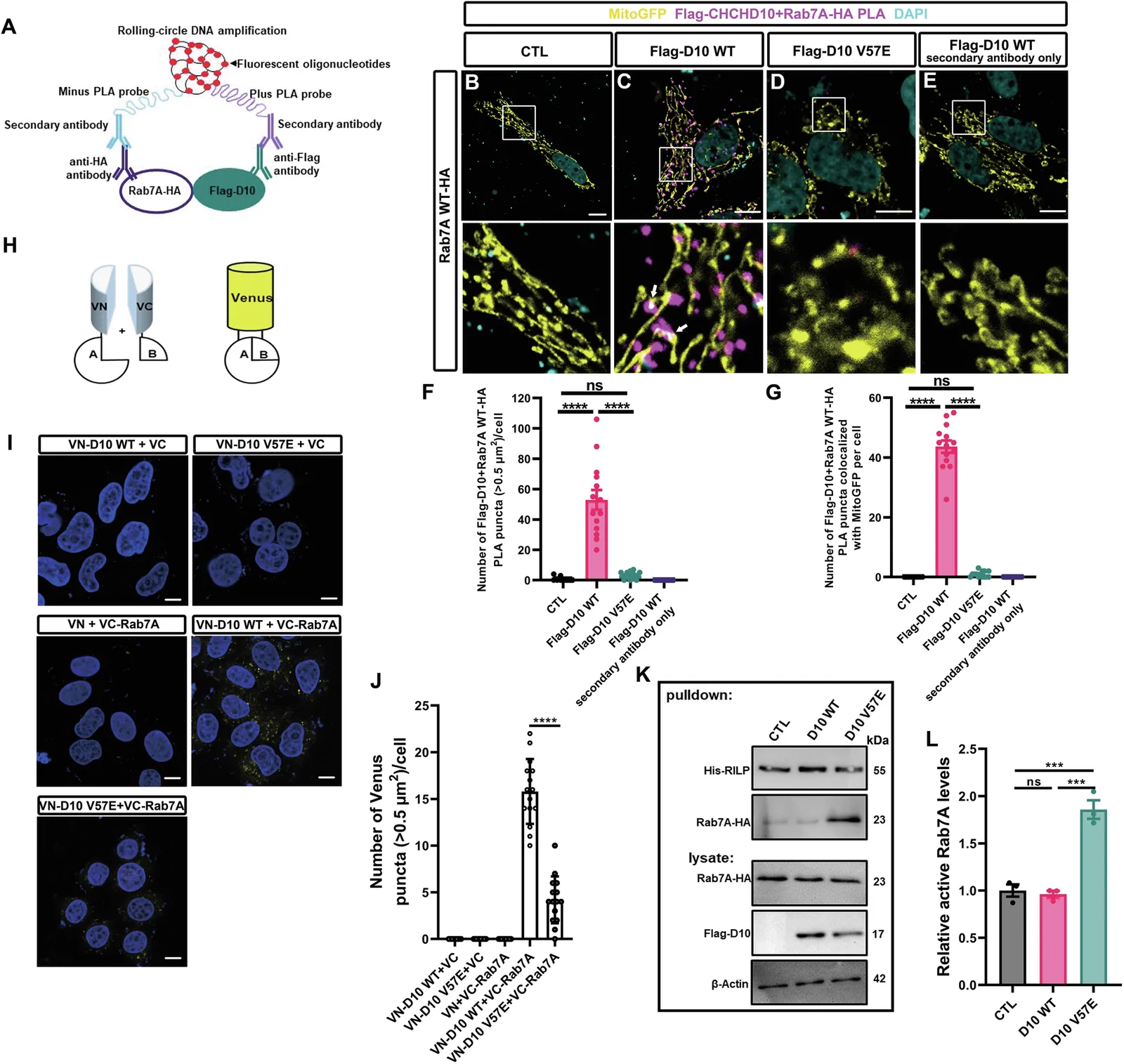
CHCHD10^V57E^ modulates Rab7A activation. **A** Schematic of the experimental principle of proximity ligation assay (PLA), exemplified by Rab7A-HA and Flag-CHCHD10. **B**–**E** HeLa cells were transfected with *Flag-CHCHD10*^*WT*^ expression construct, *Flag-CHCHD10*^*V57E*^ expression construct, or empty vector, together with *Rab7A*^*WT*^*-HA* and *MitoGFP* constructs. Subsequently, the PLA was performed, and the representative PLA images were shown. **F** Quantification of Flag-CHCHD10+Rab7A-HA PLA puncta count (>0.5 µm²) per cell in (**B**–**E**) (one-way ANOVA with Bonferroni’s multiple comparisons test, *n* = 14, ****p < 0.0001; ns non-significant). **G** The number of Flag-CHCHD10+Rab7A-HA PLA puncta colocalized with MitoGFP was quantified (one-way ANOVA with Bonferroni’s multiple comparisons test, *n* = 14, ****p < 0.0001; ns, non-significant). **H** The schematic overview of the experimental principle of the bimolecular fluorescence complementation assay. **I** Venus fluorescent images with the co-expression of the indicated plasmids in HeLa cells. **J** Quantification of Venus puncta count (>0.5 µm²) per cell in (**I**) (Student’s *t*-test, *n* = 15, ****p < 0.0001). **K** Lysates from HeLa cells expressing *Flag-CHCHD10*^*WT*^, *Flag-CHCHD10*^*V57E*^, or empty vector only were incubated with His-tagged RILP recombinant protein. Subsequently, the samples were subjected to anti-His pull-down assay. Both 10% of cell lysates and immuno-purified His-HA complexes were analyzed by immunoblot. **L** Quantification of active Rab7A levels in HeLa cells expressing *Flag-CHCHD10*^*WT*^, *Flag-CHCHD10*^*V57E*^, or empty vector only (one-way ANOVA with Bonferroni’s multiple comparisons test, *n* = 3, ***p < 0.001; ns non-significant).

Previous reports have shown that mitochondria and endosomes/lysosomes can form dynamic inter-organelle membrane contacts through mitochondrial outer membrane proteins and endo-lysosomal protein Rab7A [46, 57]. Given that CHCHD10 localizes partially to the mitochondrial outer membrane, we hypothesized that CHCHD10 may interact with Rab7A. To test this hypothesis, we first assessed potential physical interactions between Rab7A and either CHCHD10^V57E^ or wild-type CHCHD10. Our immunoprecipitation assay failed to detect these interactions (Fig. S7), suggesting no strong direct binding or that their interaction is transient and weak. We therefore performed a proximity ligation assay (PLA) to probe for the potential interactions, as PLA detects transient and weak physical interactions [58]. When candidate proteins are in close spatial proximity, the DNA probes hybridize to form circular DNA, enabling rolling-circle amplification. The amplified DNA can then be labeled with fluorescent oligonucleotides (Fig. 6A). We transfected the *Rab7A-HA* and *MitoGFP* plasmids into HeLa cells stably expressing *Flag-CHCHD10*^*WT*^, *Flag-CHCHD10*^*V57E*^, or control Flag tag only. Subsequently, the proximity ligation assay was performed to measure the number of fluorescent puncta. We observed a significantly increased number of fluorescent puncta in CHCHD10^WT^/Rab7A co-expressing cells compared to controls (Fig. 6B, C, F). However, the puncta number was markedly reduced in CHCHD10^V57E^/Rab7A co-expressing cells compared to the CHCHD10^WT^/Rab7A co-expressing cells (Fig. 6D–F), indicating that the CHCHD10^V57E^ mutation disrupts the proximity between Rab7A and CHCHD10. In addition, we found that the PLA puncta colocalized with MitoGFP in cells expressing Flag-CHCHD10^WT^, suggesting that the CHCHD10^WT^-Rab7A proximity occurred on the mitochondria (Fig. 6C, G).

To further confirm the close proximity between CHCHD10^WT^ and Rab7A, we performed bimolecular fluorescence complementation (BiFC) analysis, which is sensitive enough to catch weak and transient protein-protein interactions [59]. We generated transgenes for *CHCHD10*^*WT*^ and *CHCHD10*^*V57E*^ with C-terminal Venus N-terminal fragment (VN) tags, and for Rab7A with a C-terminal Venus C-terminal fragment (VC) tag. Protein-protein interactions will reconstitute the fluorescent Venus protein (Fig. 6H). Similar to PLA results, distinct fluorescence signals were detected in cells expressing CHCHD10^WT^ and Rab7A, whereas cells co-expressing CHCHD10^V57E^ and Rab7A exhibited significantly reduced fluorescence puncta number (Fig. 6I, J). In addition, we also validated the close proximity between CHCHD10^WT^ and Rab7A in rat cortical primary neurons. We then generated lentivirus encoding Flag-CHCHD10^WT^ and Flag-CHCHD10^V57E^, or control lentivirus. Proximity ligation assay was performed by using anti-Flag and anti-Rab7A antibodies. We found that the number of Flag-CHCHD10-Rab7A PLA fluorescent puncta was markedly increased in CHCHD10^WT^-expressing neurons than in controls (Fig. S8A, B). However, the puncta number was significantly reduced in CHCHD10^V57E^-expressing neurons compared with that in CHCHD10^WT^-expressing neurons (Fig. S8A, B), indicating that CHCHD10^WT^ was in proximity with Rab7A in the neurons. In contrast, the CHCHD10^V57E^ mutant loses the ability to interact with Rab7A. Taken together, these data solidify the notion that CHCHD10^WT^ binds Rab7A with low affinity or transiently, whereas the CHCHD10^V57E^ mutant fails to interact with Rab7A.

### CHCHD10^V57E^ modulates Rab7A activation

Rab7A cycles between GDP-bound (inactive) and GTP-bound (active) states, and constitutively active Rab7A enhances tau secretion [55]. To determine whether Rab7A activity contributes to tau pathology in *Drosophila*, we expressed a constitutively active Rab7 mutant (Rab7^Q67L^) alone or together with human Tau^R406W^ using the *GMR-Gal4* driver. Expression of Rab7^Q67L^ alone induced rough eye phenotype (REP) (Fig. S9A, B, G), which is consistent with a recent report that has implicated Rab7^Q67L^ in synapse growth defects in *Drosophila* [60]. As a positive control for active Rab7A involved in tauopathies, overexpression of Rab7A^Q67L^, induced a prominent increase in severe eye defects in Tau^R406W^ *Drosophila* (Fig. S9D, E, G). By contrast, overexpression of Rab7A^T22N^, a GDP-locked Rab7A mutant, partially alleviated eye defects in the same Tau^R406W^ *Drosophila* model (Fig. S9F, G). Collectively, these results suggested that the Tau^R406W^-induced rough eye phenotypes are dependent on Rab7 GTPase activity.

Given that *Rab7A* depletion partially rescued the tauopathy phenotypes in *CHCHD10*^*V57E*^-expressing cells (Fig. 5), we hypothesized that CHCHD10^V57E^ may modulate the Rab7A GTP-bound activity states. To test this hypothesis, we performed a pull-down assay using His-tagged Rab-interacting lysosomal protein (RILP), which specifically binds Rab7A-GTP (active) but not Rab7A-GDP (inactive) [61]. Specifically, in vitro purified His-RILP (Fig. S10) was used to affinity-precipitate Rab7A-GTP from lysates of cells expressing Rab7A-HA and CHCHD10 variants (WT or V57E). We found that expression of CHCHD10^V57E^ significantly increased the co-precipitation of His-RILP with Rab7A-HA compared to control (empty vector) and CHCHD10^WT^ (Fig. 6K, L). These data strongly suggested that CHCHD10^V57E^ promotes Rab7A activation. Then, we investigated the proximity between CHCHD10^WT^ and the constitutively active mutant Rab7A^Q67L^. Proximity ligation assay did not detect a close association between these two proteins (Fig. S11A, B). Moreover, the pathogenic CHCHD10^V57E^ mutant also failed to associate with Rab7A^Q67L^ (Fig. S11A, B). These data indicate that the activation state of Rab7A impacts its interaction with CHCHD10 variants.

It is reported that the subcellular localization of Rab7A closely relates to the activity status of Rab7A [57]. Inactive Rab7A localizes to multiple endomembrane systems, which include endosomes, lysosomes, mitochondria, endoplasmic reticulum, and trans-Golgi network, whereas hyperactivated Rab7A (Rab7A-GTP) localizes exclusively to endo-lysosomal membrane [57, 62]. To test whether CHCHD10^WT^ and CHCHD10^V57E^ impact the localization of Rab7A, PLA was performed to probe for the potential proximity between Rab7A and mitochondrial/lysosomal markers in HeLa cells stably expressing Flag-CHCHD10^WT^, Flag-CHCHD10^V57E^, or control Flag tag only. Firstly, we analyzed the interaction between Rab7A and its known interactor, the mitochondrial outer membrane protein TOMM20, in cells expressing CHCHD10 variants. We found that the TOMM20-Rab7A-HA PLA fluorescent puncta were dramatically decreased in CHCHD10^V57E^-expressing cells compared to signals in the control and CHCHD10^WT^-expressing cells (Fig. S11C, D), indicating CHCHD10^V57E^ promotes dissociation of Rab7A from mitochondria. Additionally, the proximity between Rab7A-HA and lysosome marker Lamp1 was significantly enhanced in CHCHD10^V57E^-expressing cells relative to the control and CHCHD10^WT^-expressing cells (Fig. S11E, F). Collectively, these data suggested that CHCHD10^V57E^ expression may promote Rab7A translocation from mitochondria to the endo-lysosomal membranes, reflecting Rab7A activation. Taken together, our analyses demonstrate that Rab7A preferentially interacts with CHCHD10^WT^ with low affinity or transiently, whereas the CHCHD10^V57E^ mutation weakens the mutant protein’s ability to bind Rab7A while enhancing Rab7A activation.

### CHCHD10^V57E^ disrupts Rab7A-TBC1D15 complex formation on mitochondria

**Fig. 7:**
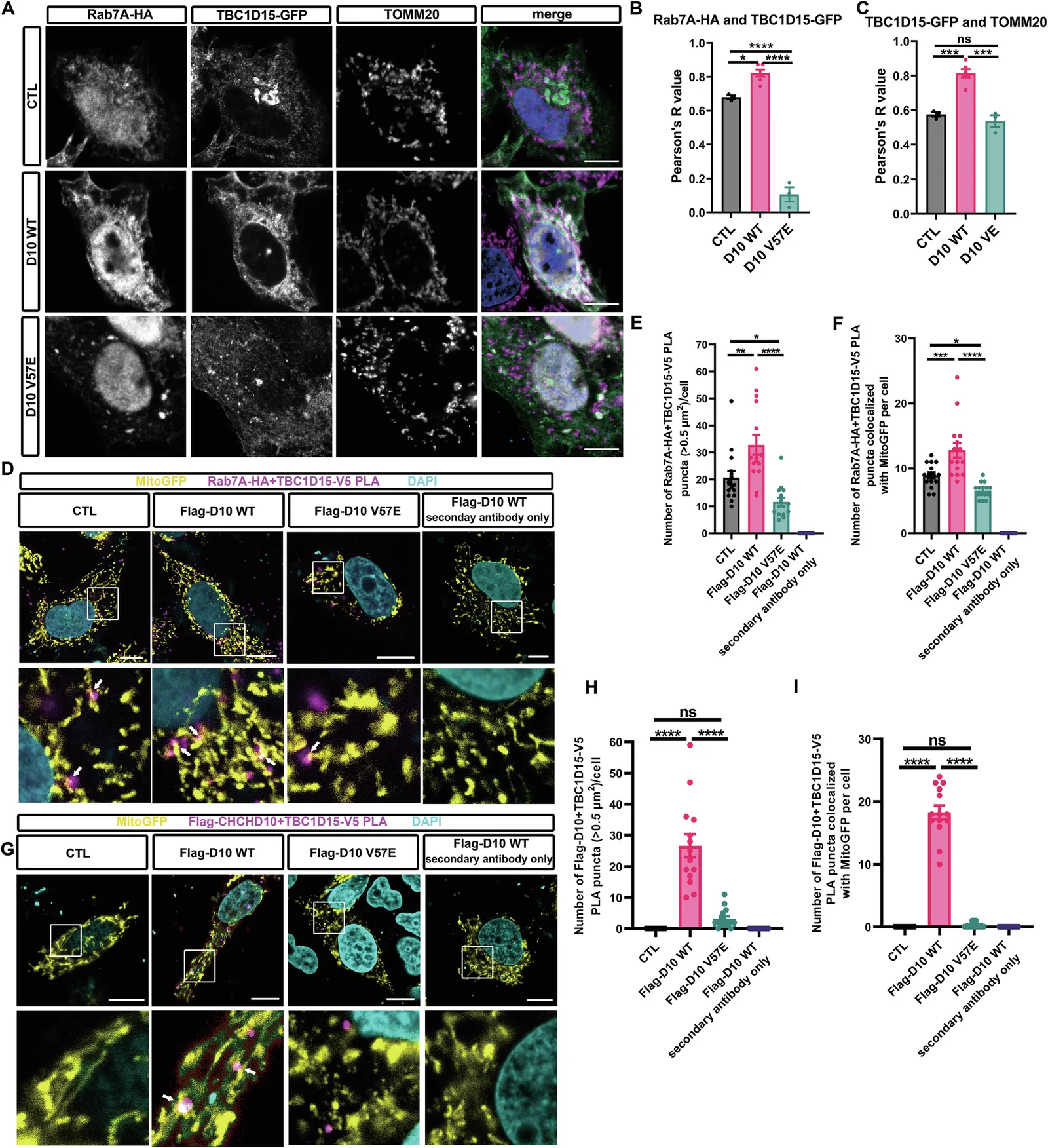
CHCHD10^WT^ enhances Rab7A-TBC1D15 interaction, whereas the V57E variant impairs the interaction. **A** HeLa cells stably expressing Flag-CHCHD10^WT^, Flag-CHCHD10^V57E^ or control vector were co-transfected with *Rab7*^*WT*^*-HA* construct and *TBC1D15-GFP* construct. GFP, anti-HA antibody, and anti-TOMM20 antibody were employed to label TBC1D15-GFP, Rab7A-HA, and mitochondria, respectively. Scale bars: 10 μm. **B**, **C** The Pearson’s correlation between Rab7A-HA and TBC1D15-GFP, or TBC1D15-GFP and TOMM20, was presented as mean ± SEM (one-way ANOVA with Bonferroni’s multiple comparisons test, *n* = 3–6, *p < 0.05; ***p < 0.001; ****p < 0.0001; ns, non-significant). **D** Representative PLA images showing the interaction between Rab7A-HA and TBC1D15-V5 complex (violet puncta) in HeLa cells. Scale bars: 10 μm. **E** Quantitative analysis of Rab7A-HA + TBC1D15-V5 PLA puncta (>0.5 µm²) per cell (one-way ANOVA with Bonferroni’s multiple comparisons test, *n* = 15, *p < 0.05; **p < 0.01; ****p < 0.0001). **F** Quantification of Rab7A-HA + TBC1D15-V5 PLA puncta colocalized with MitoGFP (one-way ANOVA with Bonferroni’s multiple comparisons test, *n* = 15, *p < 0.05; ***p < 0.001; ****p < 0.0001). **G** Representative PLA images showing the interaction between Flag-CHCHD10 variants and TBC1D15-V5 complexes (violet puncta) in HeLa cells. Scale bars: 10 μm. **H** Quantitative analysis of Flag-CHCHD10 + TBC1D15-V5 PLA puncta (>0.5 µm²) per cell (one-way ANOVA with Bonferroni’s multiple comparisons test, *n* = 14, ****p < 0.0001; ns, non-significant). **I** Quantification of Flag-CHCHD10 + TBC1D15-V5 PLA puncta colocalized with MitoGFP (one-way ANOVA with Bonferroni’s multiple comparisons test, *n* = 14, ****p < 0.0001; ns non-significant). Immunofluorescence images in (**A**, **D**, and **G**) were presented in pseudocolors to enhance accessibility.

Previous studies have shown that TBC1D15, a Rab7A GTPase-activating protein (GAP), localizes to mitochondria to regulate Rab7 inactivation through GTP hydrolysis [62, 63]. To explore the molecular mechanism of Rab7A activation, we first investigated the subcellular localization of TBC1D15-GFP and Rab7A-HA upon overexpression of CHCHD10^WT^ or CHCHD10^V57E^. Rab7A appeared to localize to a large, interconnected network spanning most of the cytosol in the control and wild-type CHCHD10-expressing HeLa cells, but not in the CHCHD10^V57E^-expressing cells (Fig. 7A). Co-staining with TBC1D15-GFP revealed that a substantial portion of it was positive for Rab7A in CHCHD10^WT^-expressing cells, but the colocalization between Rab7A and TBC1D15 was dramatically reduced in the CHCHD10^V57E^-expressing cells (Fig. 7A, B). Notably, we found that in the CHCHD10^WT^-expressing cells, the tubular distribution of TBC1D15 exhibited greater overlap with mitochondrial TOMM20 than in either control or CHCHD10^V57E^-expressing cells (Fig. 7A, C). These data suggested that TBC1D15 and Rab7A tend to co-move toward mitochondria in the CHCHD10^WT^-expressing cells, whereas they become dissociated in the CHCHD10^V57E^-expressing cells (Fig. 7A, C).

To further confirm TBC1D15 organelle localization, we co-expressed TBC1D15-GFP with either wild-type or V57E mutant Flag-CHCHD10 in HeLa cells and performed differential centrifugation-based subcellular fractionation. The immunoblot analysis confirmed that TBC1D15-GFP localized to both the cytosolic fraction (S) and mitochondria-enriched membrane fraction (P) in CHCHD10^WT^-expressing cells, but was confined to the cytoplasm in CHCHD10^V57E^-expressing cells (Fig. S12A–C).

We noted that both Rab7A and TBC1D15 tend to localize to mitochondria in cells expressing CHCHD10^WT^, whereas the correlation between Rab7A and TBC1D15 was significantly reduced in the CHCHD10^V57E^-expressing cells (Figs. 7A–C, S11C, D, S12A). Thus, we hypothesized that CHCHD10 variants may regulate Rab7A activity by modulating the interaction between Rab7A and its GAP protein TBC1D15. To test this hypothesis, HeLa cells stably expressing control, Flag-CHCHD10^WT^, and Flag-CHCHD10^V57E^ were co-transfected with *Rab7A-HA*, *TBC1D15-V5*, and *MitoGFP* constructs. Proximity ligation assays were first conducted to assess the proximity between Rab7A and TBC1D15 in the presence of CHCHD10 variants. We found that in the presence of CHCHD10^WT^, the number of Rab7A-HA-TBC1D15-V5 PLA puncta was significantly elevated compared to controls, but dramatically reduced in the CHCHD10^V57E^-expressing cells (Fig. 7D, E). Furthermore, we observed increased colocalization of Rab7A-HA-TBC1D15-V5 PLA puncta with MitoGFP in cells expressing Flag-CHCHD10^WT^, relative to both the control cells and Flag-CHCHD10^V57E^-expressing cells (Fig. 7F). We next assessed the proximity between Rab7A and TBC1D15 in rat cortical primary neurons expressing either a control vector or CHCHD10 variants. Consistent with results obtained in HeLa cells, PLA analysis showed that the proximity between Rab7A and TBC1D15 was significantly enhanced in CHCHD10^WT^-expressing neurons compared with controls (Fig. S12D, E). In contrast, the proximity between Rab7A and TBC1D15 was dramatically decreased in CHCHD10^V57E^-expressing neurons relative to CHCHD10^WT^-expressing neurons (Fig. S12D, E). Collectively, these results indicated that CHCHD10^WT^ promotes the interaction between Rab7A and its GAP TBC1D15, whereas CHCHD10^V57E^ may dampen the interaction.

### CHCHD10^V57E^ regulates Rab7A activity through TBC1D15

Given that Rab7A interacts with CHCHD10^WT^, and CHCHD10^WT^ expression promotes Rab7A-TBC1D15 interaction (Figs. 6A–J, 7A, B, D, E). We further explored the interaction between CHCHD10 variants and TBC1D15. In our immunoprecipitation assay, we did not detect any interactions between TBC1D15-V5 and the CHCHD10 variants. As a positive control, we also tested the interaction between TBC1D15-V5 and Rab7-HA to rule out technical artifacts. Thus, it suggests that the interactions between TBC1D15-V5 and the CHCHD10 variants may be weak or transient (Fig. S12G). HeLa cells were co-transfected with *TBC1D15-V5*, *MitoGFP* plus either *Flag-CHCHD10*^*WT*^, *Flag-CHCHD10*^*V57E*^, or empty vector. PLA analysis demonstrated that there was a close proximity between CHCHD10^WT^ and TBC1D15, with most of the PLA puncta also colocalizing with MitoGFP (Fig. 7G–I). In contrast, CHCHD10^V57E^ failed to bind with TBC1D15 (Fig. 7G–I). In addition, we employed the BiFC assay to confirm the proximity between CHCHD10 and TBC1D15. The results revealed that TBC1D15 interacts with CHCHD10^WT^ with low affinity or transiently, but not with CHCHD10^V57E^ (Fig. S12H, I). We further extend these analyses to rat cortical primary neurons to test the proximity between TBC1D15 and CHCHD10 variants. Primary neurons were transduced with lentivirus encoding Flag-tagged CHCHD10 variants or control lentivirus, and PLA was performed using anti-Flag and anti-TBC1D15 antibodies. We found that the number of Flag-CHCHD10-TBC1D15 PLA fluorescent puncta was markedly higher in CHCHD10^WT^-expressing neurons than in empty-vector controls (Fig. S12J, K). By contrast, the number of Flag-CHCHD10-TBC1D15 PLA puncta was significantly reduced in CHCHD10^V57E^-expressing neurons relative to CHCHD10^WT^-expressing neurons (Fig. S12J, K). Taken together, our analyses showed that CHCHD10^WT^ may function as a scaffold protein that interacts with both Rab7A and its GAP TBC1D15. In contrast, CHCHD10^V57E^ disrupts the protein complex, thereby fine-tuning the activity of Rab7A.

We next investigated whether the CHCHD10^G66V^ mutant participates in the CHCHD10-Rab7A-TBC1D15 protein complex. We transfected the *Rab7A-HA*, *TBC1D15-V5*, or both into HeLa cells stably expressing *Flag-CHCHD10*^*WT*^, *Flag-CHCHD10*^*G66V*^, or a Flag-only control, then performed proximity ligation assay as previously described. In cells expressing Flag-CHCHD10^G66V^, the number of Flag-CHCHD10-Rab7-HA PLA puncta was significantly increased relative to controls, and was similar to that detected in Flag-CHCHD10^WT^/Rab7A-HA co-expressing cells (Fig. S13A, B). Consistent with the results for CHCHD10^WT^, the Rab7A-HA-TBC1D15-V5 PLA puncta number was significantly higher in Flag-CHCHD10^G66V^/Rab7A-HA co-expressing cells than in controls (Fig. S13C, D). Additional PLA analysis further confirmed the proximity between CHCHD10^G66V^ and TBC1D15 (Fig. S13E, F). Collectively, these results indicate that the G66V mutation of CHCHD10 does not alter CHCHD10’s proximity with Rab7-TBC1D15, which also highlights the specific role of the CHCHD10^V57E^ mutant.

To further confirm the role of the CHCHD10-Rab7A-TBC1D15 protein complex in Tau-induced neuronal phenotypes, we crossed various combinations of *CHCHD10*^*WT*^, *CHCHD10*^*V57E*^, *lacZ* (control), *Tau*^*R406W*^, and *Tbc1d15-17* (the *Drosophila* homolog of mammalian Rab7-GAP TBC1D15) RNAi transgenes with *GMR-Gal4* driver flies. Adult fly heads were harvested for subsequent eye phenotype analysis. First, we confirmed Tbc1d15-17 knockdown efficiency in *Drosophila* eyes reached ~40% (Fig. S14A). Next, we found that the depletion of Tbc1d15-17 in either control, CHCHD10^WT^, or CHCHD10^V57E^ flies moderately exacerbated Tau^R406W^ retinal defects (Fig. S14B–G), as indicated by slightly increased incidence of the enhanced rough eye phenotypes (EREP) subgroup (Fig. S14H). This could be due to the fact that TBC1D15 knockdown will reduce the formation of the CHCHD10-Rab7A-TBC1D15 protein complex, leaving insufficient TBC1D15 to interact with and inhibit Rab7 activity. In addition, it has been reported that loss of Tbc1d15-17 leads to elevated Rab7 activity ^70^. Thus, the active Rab7 is likely to contribute to the enhanced tau-induced retinal defects observed in both control flies and those expressing CHCHD10 variants.

## Discussion

**Fig. 8:**
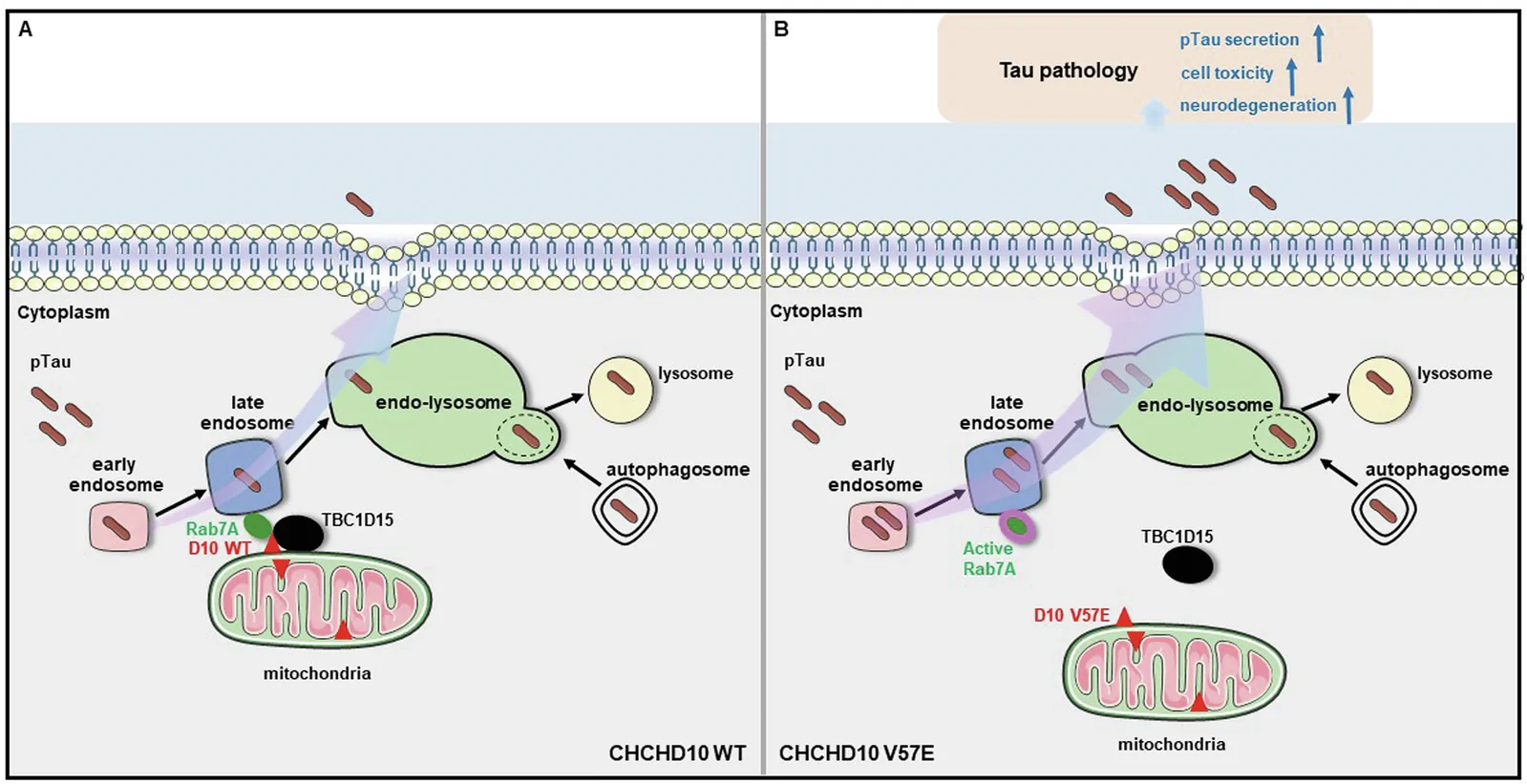
A diagram depicting the role of CHCHD10^V57E^ in tau pathology. **A** With CHCHD10^WT^ expression, the mitochondrial outer membrane-localized CHCHD10^WT^ acts as a scaffold protein, which promotes the interaction between Rab7A and TBC1D15, thereby maintaining Rab7A in an inactive state. **B** Upon CHCHD10^V57E^ expression, the mitochondrial outer membrane-localized CHCHD10^V57E^ dissociates the interaction between Rab7A and TBC1D15. Rab7A activation is enhanced, leading to an increase in the endo-lysosome-related phosphorylated tau secretion and tau-mediated neurodegeneration.

In this study, we reported that the pathological *CHCHD10*^*V57E*^ mutation exacerbates FTD-tau pathology and tau-mediated neurodegeneration. We show that overexpression of *CHCHD10*^*V57E*^ in either FTD-tau flies or tau-overexpressing mammalian cells exacerbates neurodegeneration, increases total and phosphorylated tau secretion, and enhances cell toxicity. In addition, expression of *CHCHD10*^*V57E*^ promotes phosphorylated tau accumulation within the endo-lysosomal system, while silencing *Rab7A* partially rescues the associated neurodegeneration, total and phosphorylated tau secretion, and cell toxicity phenotypes in tauopathy models expressing *CHCHD10*^*V57E*^. Regarding the molecular mechanism, we provide evidence that the CHCHD10-Rab7A-TBC1D15 complex maintains Rab7A in an inactive state. In contrast, CHCHD10^V57E^ expression dissociates the protein complex, resulting in Rab7A activation. Thus, our study uncovers that the FTD-associated CHCHD10^V57E^ mutant modulates the activation process of Rab7A, thereby promoting the development of tau pathologies (Fig. 8). However, it remains unclear whether this mechanism operates similarly in brain tissue samples from patients carrying the CHCHD10^V57E^ mutation. This will be an interesting direction for future investigation.

The CHCHD10 protein was thought to be primarily localized to the mitochondrial intermembrane space or inner membrane [64], where it serves as a key component of the mitochondrial MICOS complex [18]. Previous studies have also reported nuclear translocation of CHCHD10, enabling participation in transcriptional regulation [65]. Here, we found that CHCHD10 localizes not only to the mitochondrial inner membrane but also to the mitochondrial outer membrane (Fig. 3F–I’). Multiple mitochondrial proteins exhibit dynamic distribution across sub-compartments rather than maintaining fixed positions. For example, the Bcl-2 protein localizes to both mitochondrial outer and inner membranes, where it modulates apoptosis [66]. PINK1, a key regulator of mitophagy, is imported into mitochondria via the outer membrane TOM complex and inner membrane TIM23 complex, where it is subsequently cleaved. When mitochondria are damaged, PINK1 accumulates exclusively on the outer membrane [67, 68]. Our data showed that both endogenous and overexpressed wild-type CHCHD10 localize to the mitochondrial outer membrane, indicating that this distribution is intrinsic and unrelated to mitochondrial abnormalities (Fig. 3F). The disease-associated CHCHD10^V57E^ mutant protein exhibits a similar mitochondrial distribution to its wild-type counterpart (Fig. 3F–I’). It is reported that Mia40 primarily mediates CHCHD10 import into the mitochondrial intermembrane space [43]. However, the molecular mechanism by which CHCHD10 localizes to the mitochondrial outer membrane requires further investigation.

Mitochondrial dysfunction leads to multiple human neurodegenerative diseases. Our study revealed that expression of pathological *CHCHD10*^*V57E*^ causes mitochondrial fragmentation (Figs. 3A–D, S3A, B), yet does not markedly impact mitochondrial ATP production and membrane potential (Fig. S3C–E, K–O). This phenomenon would be one of the reasons for the absence of apparent cytotoxicity in *Drosophila* and mammalian culture cells expressing *CHCHD10*^*V57E*^ only (Figs. 1G–I, 2B–D). In addition, our assessment revealed that *CHCHD10*^*V57E*^ expression induced more pronounced intracellular ROS generation in mammalian cells relative to *Drosophila* cells (Fig. S3F–J). This suggests that different cell types may exhibit varying responses to *CHCHD10*^*V57E*^ expression. Thus, further studies are needed to determine whether *CHCHD10*^*V57E*^ causes damage in other cell types or tissues.

Tau protein lacks conventional signal sequences and is secreted via unconventional protein secretion pathways rather than the classical ER-Golgi route [41]. The endo-lysosomal pathway has been implicated as a mechanism for tau secretion [11]. We found that CHCHD10^V57E^ overexpression promotes intracellular accumulation of phosphorylated tau within the endo-lysosomal system (Figs. 4M–U’, S5). However, intracellular total tau and pTau levels remain unchanged, while the secreted tau and pTau significantly increase in the CHCHD10^V57E^-expressing cells compared to the controls (Fig. 2E–N). CHCHD10^V57E^ overexpression may facilitate pTau entry into the endo-lysosomal system and subsequent secretion into the extracellular space. This phenomenon represents a typical hallmark of tauopathies, including AD and FTD-Tau. Pathological tau is released from cells and internalized by neighboring cells, where it templates the misfolding of native tau molecules, propagating tau pathology [13, 69]. In addition, consistent with previous reports [41], we did not detect the total tau and pTau protein in the collected extracellular exosome vesicles (Fig. S2), suggesting that tau and pTau entering the endo-lysosomal system undergo direct secretion to the extracellular space rather than vesicle-mediated release.

We observed that CHCHD10^V57E^ alone has minimal effects on early or late stages of autophagy markers (Fig. S4A–O, A’–G’). In contrast, tau^R406W^ overexpression consistently promotes pTau colocalization with mCherry-Atg8a in control, CHCHD10^WT^-expressing, and CHCHD10^V57E^-expressing flies (Fig. S4P, H’). This indicates that tau^R406W^ primarily induces autophagy and facilitates pTau entry into autophagosomes. Prior studies have shown that when autophagy is impaired, misfolded or hyperphosphorylated tau protein accumulates in late endosomes and lysosomes [70–72]. This accumulation occurs due to disrupted autolysosome-mediated tau clearance [73]. Given the close functional interplay between autophagy and the endo-lysosomal system, we propose that in cells co-expressing tau^R406W^ and CHCHD10^V57E^, autophagy may act jointly with secretory endo-lysosomes to promote phosphorylated tau accumulation within the endo-lysosomal compartments.

Previous studies have shown that extracellular tau and pTau can be released both actively by living neurons and passively by dead cells [74, 75]. Notably, co-expression of CHCHD10^V57E^ and tau induced significantly more cell death than co-expression of tau with either empty vector or CHCHD10^WT^ (Figs. 2D, S6C). Thus, there is still a possibility that cell death in cells co-expressing CHCHD10^V57E^ and tau may also contribute to increased levels of total tau and pTau in the extracellular medium.

Accumulating evidence suggests that mitochondria require inter-organelle communication with the endosome or lysosome to execute their physiological functions [76, 77]. Mitochondria-endosome/lysosome interactions are essential for mitochondrial quality control, modulation of mitochondrial dynamics, intracellular cargo trafficking, and lysosomal dynamics [62, 76–78]. Mitochondrial Fis1 oligomers can also regulate Rab7 GTP hydrolysis by recruiting Rab7 GTPase-activating proteins, leading to lysosomal untethering [78]. Our study identified CHCHD10 as a novel mitochondrial regulator of Rab7A activity. Through its localization on the outer mitochondrial membrane, CHCHD10 enables contact with surface molecules of other organelles. Specifically, we found that wild-type CHCHD10 assembles with TBC1D15 and Rab7A on the mitochondria, which facilitates Rab7A GTP hydrolysis via TBC1D15 to maintain Rab7A in an inactive state (Figs. 6K, L, 7A–F, S12). In contrast, the CHCHD10^V57E^ mutant induces TBC1D15 and Rab7A leaves from mitochondria, disrupting TBC1D15-Rab7A interaction and thereby stabilizing Rab7A in its active GTP-bound conformation (Figs. 6K, L, 7A–F, S12). Our analysis revealed that the activation states of Rab7A play an important role in regulating total and phosphorylated tau secretion, which is consistent with previous findings [55, 56]. However, it remains unclear why the V57E mutation of CHCHD10 causes loss of binding to Rab7A and TBC1D15. Recent studies have revealed that the V57E mutation induces changes in the secondary and tertiary structures of CHCHD10 [26], which may alter its ability to bind other proteins. Moreover, it remains to be investigated whether other functions of CHCHD10^V57E^, such as leading to mitochondrial dysfunction, are also involved in regulating the Rab7A activity.

## Materials and methods

### Antibodies

The following antibodies were applied to probe target proteins in this research: anti-Flag-tag primary antibody 1:10000 for WB and 1:250 for IF (AlpVHHs, 016-303-001); anti-phospho-tau (Ser199, 202) primary antibody 1:1000 for WB (Invitrogen, 44–768 G); anti-tau primary antibody (A-10) 1:1000 for WB (Santa Cruz Biotechnology, sc-390476); anti-HA-tag primary antibody 1:8000 for WB and 1:200 for IF (Proteintech, 51064-2-AP); anti-HA-tag primary antibody 1:800 for IF (BioLegend, 901533); anti-V5-tag primary antibody 1:10000 for WB and 1:200 for IF (Proteintech, 14440-1-AP); anti-β actin primary antibody 1:10000 for WB and 1:500 for IF (HuaAn biotechnology, EM21002); anti-lamp1 primary antibody 1:250 for IF (Proteintech, 21997-1-AP); anti-LC3 primary antibody 1:200 for IF (Abcam, AB109364); anti-TOMM20 primary antibody 1:5000 for WB and 1:250 for IF (Proteintech, 11802-1-AP); anti-ATP5A primary antibody 1:2000 for WB and 1:200 for IF (Abcam, AB14748); anti-EndoG primary antibody 1:2000 for WB and 1:300 for IF (Proteintech, 22148-1-AP); anti-His-tag primary antibody 1:10000 for WB (AlpVHHs, 004-202-001); anti-GFP-tag primary antibody 1:200 for IF (Proteintech, 66002-1-lg); anti-Rab7A primary antibody 1:2000 for WB (Proteintech, 55469-1-AP); anti-Rab7A primary antibody 1:200 for IF (Abclonal, A28294); anti-PSD95 primary antibody 1:500 for IF (Proteintech, 81106-1-RR); anti-Synaptophysin primary antibody 1:500 for IF (Sangon Biotech, D264558); anti-CD9 primary antibody 1:2000 for WB (Proteintech, 20597-1-AP); anti-CHCHD10 primary antibody 1:2000 for WB and 1:200 for IF (Proteintech, 22148-1-AP); anti-TBC1D15 primary antibody 1:400 for IF (Proteintech, 17252-1-AP); HRP conjugated goat anti-rabbit IgG antibody 1:10000 (HuaAn biotechnology, HA1001); HRP conjugated goat anti-mouse IgG antibody 1:10000 (HuaAn biotechnology, HA1006); Alexa Fluor™ 488-conjugated anti-rabbit recombinant secondary antibody 1:1000 (Invitrogen, A-21206); Alexa Fluor™ 488-conjugated anti-mouse recombinant secondary antibody 1:1000 (Invitrogen, A-21202); Alexa Fluor™ 594-conjugated anti-rabbit recombinant secondary antibody 1:1000 (Invitrogen, A-21207); Alexa Fluor™ 594-conjugated anti-mouse recombinant secondary antibody 1:1000 (Invitrogen, A-21203); Alexa Fluor™ 647-conjugated anti-rabbit recombinant secondary antibody 1:1000 (Invitrogen, A-31573); Alexa Fluor™ 647-conjugated anti-mouse recombinant secondary antibody 1:1000 (Invitrogen, A-21235).

### Molecular cloning

Constructs used for fly studies and mammalian cell studies are generated according to the standard protocols. Specifically, the cDNA of CHCHD10, 0N4R Tau, Rab7A, Rab5, and TBC1D15 was amplified by using the indicated primers in Table supplementary 1. The amplified PCR products were inserted into digested plasmids. All constructs were verified by DNA sequencing (GENEWIZ, Suzhou, China). The plasmid of pcDNA3.1-MitoGFP was a kind gift from Prof. Dr. Chao Tong (ZJU, Hangzhou, China). The pLV2-TRE3GS-EGFP-TBC1D15-TetOne-Puro and pLV3-CMV-mCherry-LAMP1(human)-Puro constructs were purchased from Miaoling Biology (Wuhan, Hubei, China). The pET-28a-His-RILP construct was made by Generalbio (Chuzhou, Anhui, China). CHCHD10 V57E and Rab7A Q67L mutants were generated by site-directed mutagenesis using primers indicated in Table supplementary 1. Mutations were verified by DNA sequencing (GENEWIZ, Suzhou, China). pcDNA3.1-TBC1D15-V5, pUAS-attB-Rab7^T22N^-V5, pLVX-IRES-ZsGreen, pLVX-IRES-Flag-CHCHD10 WT-P2A-ZsGreen, pLVX-IRES-Flag-CHCHD10^V57E^-P2A-ZsGreen, and p3x-Flag-CHCHD10^G66V^ constructs were purchased from GENEWIZ (Suzhou, China).

### *Drosophila* genetics

The fly stocks and fly genotypes used in this study are listed in Additional file 1. Flies were reared at room temperature using standard fly food media. The composition of the fly food is as follows: sucrose (40 g/L), dry yeast (25 g/L), cornmeal (66.8 g/L), soy flour (9.1 g/L), agar (6 g/L), maltose (42.4 g/L), sodium benzoate (1 g/L), propionic acid (6.8 mL/L), and 15% nipagin solution (2.5 mL/L).

### Cell culture

HeLa cells were cultured in Dulbecco’s modified Eagle’s medium (DMEM, Bio-Channel, BC-M-005), supplemented with 10% fetal bovine serum (FBS, Bio-Channel, BC-SE-FBS01) and 1% penicillin/streptomycin (P/S, Bio-Channel, Pricella, PB180120).

### Primary neuronal culture

Primary cortical neurons were prepared from isolated embryonic day-18 (E18) Sprague-Dawley (SD) rats. Cortical tissues were dissected in ice-cold Dulbecco’s modified Eagle medium (DMEM; Gibco, 12800017) and digested with 0.25% trypsin-EDTA (Gibco, 25200072) at 37 °C for 15 min. Dissociated neurons were plated onto poly-D-lysine-coated dishes (35 mm diameter) at densities of 1.0 × 10⁵/cm², and initially cultured in DMEM supplemented with 10% horse serum (Gibco, 16050130) for 2–4 h. Subsequently, the medium was replaced with Neurobasal medium (Gibco, 21103049) containing 2% B27 (Gibco, 17504044) and 0.5 mmol/L GlutaMAX (Gibco, 35050061). Half of the medium was refreshed twice per week. At 6 days in vitro, neurons were subjected to viral infection.

### DNA/siRNA transfection and lentivirus transduction

HeLa cells were transfected with DNA plasmids or siRNA using Lipo8000 (Beyotime, C0533). Primary rat cortical neurons were transduced with lentivirus. The siRNA used to knock down Rab7A in HeLa cells is targeting 5’-gggagauucuggagucgggaa-3’.

### Fly climbing assay

We crossed the indicated genotypes of flies with the *elav-Gal4* driver. Sixty male flies per genotype were collected and divided into groups of 10. Flies were randomly assigned to six groups using a random number generator. All flies (1–3 days post-exclusion) were maintained at 25 °C. Climbing ability was assessed in adult flies at 4, 8, and 16 days of age. The investigator performing the fly climbing assay was blinded to fly genotypes.

### Fly lifespan assay

We crossed the indicated genotypes of flies with neuronal-specific elav-Gal4 driver. Eighty male flies per genotype were collected and divided into groups of 20. All flies (1–3 days post-eclosion) were maintained at 25 °C with 60% of humidity and in a 12 h day-night cycle based on standard *Drosophila* food. Flies were transferred to fresh food every 2–3 days, with mortality recorded until all flies were sacrificed. The investigator performing the fly lifespan assay was blinded to fly genotypes.

### Tau secretion analysis

Collected cells were lysed with RIPA buffer supplemented with protease inhibitors (MCE, HY-K0010) and phosphatase inhibitors (Proteintech, PR20015) for 20 min on ice. Lysates were centrifuged at 13,000×*g* for 10 min at 4 °C, and supernatants were collected. Samples were mixed with Laemmli sample buffer (50 mM Tris, pH 6.8, 2.5% β-Mercapto-ethanol, 2% SDS, 0.02% Brom phenol blue, and 10% Glycerol), and intracellular phosphorylated tau (pTau), total tau, β-actin, and Flag-CHCHD10 protein levels were analyzed by western blotting.

For extracellular tau analysis, cell culture media were centrifuged at 1000×*g* for 10 min at 4 °C. Supernatants were incubated overnight at 4 °C with 30 μL Protein A + G Agarose (Beyotime, P2055) and 0.5 μg V5-tag antibody (Proteintech, 14440-1-AP) under constant rotation. Beads were washed 4 times and pelleted. SDS sample buffer was added to the beads, and extracellular pTau and total tau levels were assessed via western blotting.

To analyze whether tau is secreted via exosomes, cell culture media were collected, and the exosomes were isolated using the Exosome Isolation Kit (Cusabio, CSB-EI0110). The exosome-free serum was incubated overnight at 4 °C with 30 μL Protein A + G Agarose (Beyotime, P2055) and 0.5 μg V5-tag antibody (Proteintech, 14440-1-AP) under constant rotation. Beads were washed and pelleted on the following day. Phosphorylated tau and total tau levels on both exosomes and exosome-free serum were determined by western blotting.

### Scanning electron microscopy (SEM)

Flies (*n* = 6) were mounted on the specimen stage and gold sputter-coated to a 4 nm thickness using an ACE600 high-vacuum coater (Leica EM, Germany). Post-coating, eye morphology was examined using a Teneo VS scanning electron microscope (FEI, USA).

### Immunofluorescence assay for *Drosophila* fat body tissues

For *Drosophila* fat body analysis, dissected fat body tissues from 15 flies were fixed in 4% PFA at room temperature for 45 min, then washed three times with PBX (0.1% Triton X-100 in PBS). Tissues underwent direct DAPI staining before mounting onto objective slides (Sail Brand, 7101) using an anti-fade mounting medium (MCE, HY-K1042) and square coverslips (Merck, BR470050). Images were acquired with a Zeiss LSM710 confocal microscope (Germany) equipped with a Plan-Apochromat 63x/1.40 Oil DIC M27 objective lens. All images were captured at a resolution of 1024 × 1024 pixels.

To analyze the number of specific markers (e.g., GFP-Rab5, GFP-Rab7, Lamp1, mCherry-Atg8a, GFP-Atg8a, and GFP-Atg5) puncta formed in each cell, images were imported into ImageJ software. The color channels were split to isolate the channel in which the signal represents the specific marker. The threshold was adjusted to include the signals of interest. Images were calibrated according to the scale bar of each image, which enables the unit of measurement to be converted from μm^2^ to pixel^2^. Particles with an area exceeding a defined threshold (e.g., 0.5 or 1 μm²) were then selected. The “Count” value was then used for quantification. To analyze the colocalization of tandem reporter (e.g., the number of GFPRFP-Atg8a puncta per cell), overlapping of red (pseudo-colored violet) and green (pseudo-colored yellow) signals, appeared as yellow (pseudo-colored white), were counted for further quantification.

Pearson’s correlation coefficient was used to determine colocalization of different signals (e.g., the colocalization between GFP-Rab7 and pTau). Images were imported into ImageJ software, and color channels were then split to separate the green (pseudo-colored yellow) signal (GFP-Rab7) from the red (pseudo-colored violet) signal (pTau). Using the “Colocalization Finder” plugin, the red channel image was assigned to “Image 1” and the green channel image to “Image 2.” In the resulting composite image, regions corresponding to cells of interest were selected, and Pearson’s correlation coefficient was measured to assess the degree of colocalization.

### Immunofluorescence assay for HeLa cells

HeLa cells cultured on φ14 mm cover slip (CITOGLAS, 10210014CE) were fixed in 4% paraformaldehyde (PFA) for 30 min, permeabilized with 0.1% Triton X-100 for 10 min, and blocked with 5% bovine serum albumin (BSA) for 30 min. Following primary antibody incubation overnight at 4 °C, cells were stained with secondary antibodies targeting specific primaries for 1 h at room temperature. Nuclei were counterstained with DAPI. The samples were then mounted onto objective slides (Sail Brand, 7101) using an anti-fade mounting medium (MCE, HY-K1042). Images were acquired with a Zeiss LSM710 confocal microscope (Germany) equipped with a Plan-Apochromat 63x/1.40 Oil DIC M27 objective lens. All images were captured at a resolution of 1024 × 1024 pixels.

To assess the colocalization of Rab5-HA and pTau, Rab7-HA and pTau, Lamp1 and pTau in HeLa cells, images were imported into ZEN 2.3 (blue edition) software. Using the “Profile” tool, regions of interest were selected, and signal intensity profiles along a defined distance were obtained. The resulting data were subsequently plotted in GraphPad Prism, with distance on the x-axis and signal intensity on the y-axis.

Pearson’s correlation coefficient was used to determine the degree of colocalization between Rab7-HA and TBC1D15-GFP or between TBC1D15-GFP and TOMM20, and the procedure was similar as described above.

### ATP assay

ATP production level was measured by utilizing ATP testing assay kit (Beyotime Biotechnology, S0026) and BCA protein quantification kit (Abbkine, Georgia, USA). For mammalian cells and neurons, ice-cold ATP lysis buffer was used for cell collection and lysis. *Drosophila* fat body tissues from 30 flies were dissected from third-instar larvae in chilled PBS, followed by ice-cold ATP lysis buffer homogenization. Lysates underwent centrifugation at 12,000×*g* (4 °C, 10 min), with subsequent collection. Simultaneous ATP and protein concentration measurements were performed using a GloMax® Discover microplate reader (Promega, USA). Final ATP concentrations were normalized as nmol per mg protein.

### ROS assay

Reactive oxygen species (ROS) levels were measured using an ROS assay kit (Beyotime Biotechnology, S0033S). For HeLa cell analysis, cells were washed and stained with the cell-permeable fluorescent probe 2’,7’-dichlorodihydrofluorescein (DCFH) for 20 min at 37 °C. Following final washing, cellular ROS levels were quantified using a BD FACSCanto™ II flow cytometer (BD Biosciences, San Jose, CA). For the *Drosophila* fat body, third-instar larval tissues from 15 flies were dissected in chilled Schneider’s insect medium (Sigma-Aldrich, S0146). After washing, tissues underwent identical DCFH staining (20 min, 25 °C) and mounted onto objective slides (Sail Brand, 7101) using an anti-fade mounting medium (MCE, HY-K1042) and square coverslips (Merck, BR470050). For neurons, cells cultured on φ14 mm cover slip (CITOGLAS, 10210014CE) were stained with DCFH (30 min, 37 °C) and then mounted onto objective slides (Sail Brand, 7101) using an anti-fade mounting medium (MCE, HY-K1042). Images were acquired with a Zeiss LSM710 confocal microscope (Germany) equipped with an EC Plan-Neofluar 40x/1.30 Oil DIC M27 objective lens. All images were captured at a resolution of 1024 × 1024 pixels. Images were imported into ImageJ software. The color channels were split to isolate the green channel, and the threshold was adjusted to ensure the signal represents the DCF fluorescence. The integrated density value was used for quantification.

### Mitochondrial membrane potential measurement

Cells were loaded with 500 nM TMRE (Beyotime, C2001S), a mitochondrial membrane potential-sensitive fluorescent dye, through 20-min incubation at 37 °C. Following final washing, cellular TMRE fluorescence levels were quantified using a BD FACSCanto™ II flow cytometer (BD Biosciences, San Jose, CA). For *Drosophila* fat body, 3rd-instar larval tissues from 15 flies were dissected in chilled Schneider’s insect medium (Sigma-Aldrich, S0146). After washing, tissues underwent identical TMRE staining (20 min, 25 °C) and mounted onto objective slides (Sail Brand, 7101) using an anti-fade mounting medium (MCE, HY-K1042) and square coverslips (Merck, BR470050). For neurons, cells cultured on φ14 mm cover slip (CITOGLAS, 10210014CE) were stained with 500 nM TMRE (45 min, 37 °C) and then mounted onto objective slides (Sail Brand, 7101) using an anti-fade mounting medium (MCE, HY-K1042). Images were acquired using a Zeiss LSM710 confocal microscope (Germany) equipped with an EC Plan-Neofluar 40x/1.30 Oil DIC M27 objective lens. All images were captured at a resolution of 1024 × 1024 pixels. Images were imported into ImageJ software. The color channels were split to isolate the red channel, and the threshold was adjusted to ensure the signal represents the TMRE fluorescence. The integrated density value was used for quantification.

### Lactate dehydrogenase (LDH) cytotoxicity assay

Cellular toxicity was determined by using the LDH Cytotoxicity Assay Kit with WST-8 (Beyotime Biotechnology, C0018M). Specifically, cell cultures with indicated gene modifications were cultured in duplicate. One portion was treated with LDH release agent for subsequent measurement of total LDH levels, while culture medium was added to the other portion. After incubation at room temperature for 15 min, the LDH detection working solution was added to each well, and the samples were incubated at room temperature protected from light for 25 min. The reaction was terminated by adding the stop solution. Absorbance was measured at 450 nm. Cytotoxicity level is determined as the ratio of the sample group absorbance to total LDH absorbance from fully lysed cells.

### Proximity ligation assay

HeLa cells stably expressing Flag-CHCHD10^WT^, Flag-CHCHD10^V57E^, or control cells were cultured on φ14 mm cover slip (CITOGLAS, 10210014CE) and were transfected with Rab7A-HA construct and MitoGFP construct. The proximity between Rab7A-HA and Flag-CHCHD10^WT^ or Flag-CHCHD10^V57E^ was analyzed using Duolink™ proximity ligation assay (PLA) technology with species-specific secondary antibodies (Sigma-Aldrich, DUO92001/DUO92005/DUO92008). Fixed cells were sequentially incubated with primary antibodies against target proteins, followed by PLA probe-conjugated secondary antibodies. Antibody pairs within <40 nm proximity underwent enzymatic ligation to form circular DNA templates, enabling subsequent rolling-circle amplification via polymerase. Nuclear counterstaining was performed using DAPI and then mounted onto objective slides (Sail Brand, 7101) using an anti-fade mounting medium (MCE, HY-K1042). The fluorescent signals were visualized through a Zeiss LSM710 confocal system (Germany), equipped with a Plan-Apochromat 63x/1.40 Oil DIC M27 objective lens. All images were captured at a resolution of 1024 × 1024 pixels.

Images were imported into ImageJ software. The color channels were split to isolate the red channel, and the threshold was adjusted to include the signals of interest. Images were calibrated according to the scale bar of each image, which enables the unit of measurement to be converted from μm^2^ to pixel^2^. Particles with an area exceeding 0.5 μm² were then selected. The “Count” value was used for quantification. To analyze the number of PLA puncta colocalized with MitoGFP, the white dot signals, representing the colocalization of red PLA puncta (pseudo-colored violet) and MitoGFP signals (pseudo-colored yellow), were counted.

### Quantitative real-time PCR assay

Total RNA was isolated from HeLa cells or flies with Trizol reagent (Invitrogen, 15596018). cDNA was reverse transcribed with the SweScript RT I First Strand cDNA Synthesis Kit (Servicebio, G3330). Briefly, 2 μg of RNA was mixed with Random Hexamer Primer, incubated at 65 °C for 5 min, and then combined with SweScript RT I Enzyme Mix and reaction buffer. The reaction was performed at 25 °C for 5 min, 55 °C for 30 min and 85 °C for 5 s in PCR machine (Life Technologies, 4483636). Quantitative PCR analysis was conducted with Perfect Start® Green qPCR Super Mix (TransGen Biotech, AQ601-01-V2) on an Applied Biosystems Step One Plus™ Real-Time PCR system (Carlsbad, CA). Relative mRNA levels from HeLa cells and flies were normalized by the endogenous Act57B and RP49 mRNA levels, respectively. qPCR reactions were in triplicate for every experiment. The forward primer 5’-gaccaaggaggtgatggtgg-3’ and the reverse primer 5’-cacaccgagagactggaacc-3’ were used to detect Rab7A mRNA, and the forward primer 5’-catcctggcctctctgtcca-3’ and reverse primer 5’-gaccagatgagcaggagaca-3’ were used for Act57B. The forward primer 5’-atcggttacggatcgaacaa-3’ and the reverse primer 5’-gacaatctccttgcgcttct-3’ were used to detect RP49 mRNA. The forward primer 5’-cggacgtaagaaatccctactga-3’ and the reverse primer 5’-attggttggagaagcgtttgt-3’ were used for Rab7 mRNA analysis. The forward primer 5’-aacactagacccattgaaccga-3’ and the reverse primer 5’-cgcacctctacagaagacaaatc-3’ were for TBC1D15 mRNA analysis in flies.

### Rab7A-GTP pull-down assay

Cell pellets were harvested and lysed in binding buffer (50 mM Tris-HCl pH 7.5, 150 mM NaCl, 0.5% NP-40, 10% glycerol, 5 mM MgCl₂, 1 mM DTT). For affinity purification, 2 μg His-tagged RILP recombinant protein was combined with 480 mg total cellular protein extract for overnight incubation at 4 °C. Magnetic His-Tag isolation beads (Beyotime Biotechnology, P2135) were subsequently added and incubated with rotation for 1 h at 4 °C. Following magnetic separation, the bead-protein complexes underwent three sequential washes with ice-cold binding buffer. Bound proteins were eluted in 2× Laemmli buffer and subjected to SDS-PAGE immunodetection.

### Co-immunoprecipitation assay

Cells were harvested and lysed in 200 µL ice-cold Lysis buffer (10 mM Tris-HCl pH 7.5, 150 mM NaCl, 0.5% NP-40, 0.5 mM EDTA) with a supplement of protease inhibitor cocktail (MCE, HY-K0010) and 1 mM PMSF (MCE, HY-B0496). After 30 min of incubation on ice, the lysates were cleared by centrifugation at 16,000×*g* for 10 min at 4 °C, and 300 µL Dilution buffer (10 mM Tris-HCl pH 7.5, 150 mM NaCl, 0.5 mM EDTA) containing protease inhibitor cocktail and 1 mM PMSF was added. A 25 µL aliquot of the diluted lysate was saved as the input fraction. The remaining diluted lysate was then mixed with the 25 µL prepared Binding control magnetic beads (ALPVHHS, 100-100-200) or DYKDDDDK tag Nanoselector Magnetic beads (ALPVHHS, 016-101-003) or V5 tag Nanoselector Magnetic beads (ALPVHHS, 064-101-003), followed by incubation with gentle rotation at 4 °C overnight to allow binding. After incubation, the beads were washed 3–5 times with 500 µL of ice-cold Dilution buffer, with magnetic separation between washes. Bound proteins were eluted by re-suspending the beads in 60 µL of 2× SDS-sample buffer and boiling at 95 °C for 5 min. The beads were then separated magnetically, and the resulting supernatant was analyzed by immunoblotting.

### Bimolecular fluorescence complementation assay (BiFC)

pBiFC-VN173 and pBiFC-VC155 were kind gifts from Chang-Deng Hu (Addgene plasmid #22010 and #22011)1. The BiFC technique utilizes protein-protein interactions to mediate the assembly of non-fluorescent Venus N-terminal (VN) and C-terminal (VC) fragments, thereby reconstituting the Venus fluorescent protein through fragment complementation. Flag-CHCHD10 WT/V57E was constructed into pBiFC-VN173, and HA-Rab7A or HA-TBC1D15 was fused to pBiFC-VC155 by using primers in supplementary Table 1. HeLa cells cultured on cover slip (CITOGLAS, 10210014CE) were transfected with pBiFC plasmids and cultured for 48 h. Cells were then fixed in 4% paraformaldehyde, and nuclei were counterstained with DAPI. Samples were then mounted onto objective slides (Sail Brand, 7101) using an anti-fade mounting medium (MCE, HY-K1042). Images were acquired with a Zeiss LSM710 confocal microscope (Germany) equipped with a Plan-Apochromat 63x/1.40 Oil DIC M27 objective lens. All images were captured at a resolution of 1024 × 1024 pixels.

Images were imported into ImageJ software. The color channels were split to isolate the green channel, and the threshold was adjusted to include the signals of interest. Images were calibrated according to the scale bar of each image, which enables the unit of measurement to be converted from μm^2^ to pixel^2^. Particles with an area exceeding 0.5 μm² were then selected. The “Count” value was used for quantification.

### Mitochondria isolation and proteinase K digestion assay

Mitochondrial purification was performed using the Cell Mitochondria Isolation Kit (Beyotime Biotechnology, C3601). Briefly, harvested cells were quantified using a Countess™ II Automated Cell Counter (Invitrogen, USA). The cell pellet was resuspended in ice-cold mitochondrial isolation buffer and maintained at 4 °C for 15 min. Following mechanical homogenization, the lysate underwent sequential centrifugation: primary centrifugation at 600×*g* (4 °C, 10 min) to pellet cellular debris, with supernatant retained; secondary centrifugation at 11,000×*g* (4 °C, 10 min) to separate cytosolic (supernatant) and mitochondrial (pellet) fractions.

Mitochondrial fraction was resuspended in isotonic mitochondrial buffer, and an increasing concentration of proteinase K (Yaxin Bio, RPK09) was added and treated for 16 min on ice. PMSF of 5 mM (Beyotime, C3601) was utilized to terminate the digestion. The mitochondria were collected by centrifugation at 13,000×*g* for 20 min at 4 °C. Mitochondria were resuspended in the isotonic mitochondrial buffer, and mitochondrial protein markers localized to the outer membrane, intermembrane space, and inner membrane were detected by Western blotting. Immunoblot images were imported into ImageJ for quantitative analysis. All images were converted to 8‑bit format, and the band intensity of each target protein was quantified using the lane plot function. The residual protein levels in each proteinase K‑treated group were normalized to the untreated control (0 μg/mL proteinase K), and results are presented as the relative amount of remaining protein (mean ± SEM, *n* = 3 independent experiments). Statistical significance was assessed by one‑way ANOVA with Bonferroni’s multiple comparisons test against the untreated control.

### Protein expression and purification

The recombinant His-tagged Rab-interacting lysosomal protein (RILP) was expressed by transforming pET-28a-RILP into Escherichia coli BL21 Rosetta 2(DE3) competent cells (Sigma-Aldrich, USA). Bacterial cultures were grown in media supplemented with 33 μg/mL chloramphenicol and 10 μg/mL kanamycin. Protein expression was induced with 0.5 mM isopropyl β-D-1-thiogalactopyranoside (IPTG) at 20 °C for 18 h. Cells were harvested and lysed using a Q125 ultrasonic homogenizer (Homogenizers, Inc., USA) with pulse cycles of 2 s on/1 s off at 80% amplitude during 30-min ice-cooled sonication. Soluble fractions were isolated through centrifugation at 16,000×*g* for 20 min at 4 °C. His-RILP was affinity-purified using Ni Sepharose™ 6 Fast Flow resin (Thermo Fisher Scientific, USA) and eluted with 100 mM imidazole in buffer (50 mM Tris-HCl pH 7.5, 150 mM NaCl). Imidazole removal was achieved through centrifugal filtration (30 kDa MWCO; Sigma-Aldrich) followed by buffer exchange into storage buffer (20 mM Tris-HCl pH 7.5, 150 mM NaCl) containing protease inhibitors. Protein purity was verified by Coomassie blue-stained SDS-PAGE, with identity confirmation via immunoblotting.

### Statistical analysis

Experiments in this research were repeated at least three times. No statistical methods were used to predetermine sample sizes on the fly climbing assay and fly lifespan assay, but our sample sizes are similar to those reported in previous publications [31, 52]. The *Drosophila* eye phenotype was quantified by the Chi-square test. For the climbing assay, significance was determined by one-way ANOVA with Bonferroni’s multiple comparisons test. Lifespan curves represent average percent survival, and significance was assessed using the Log-rank test. Data were presented as mean ± SEM and analyzed by two-tailed unpaired Student’s *t*-test or ordinary one-way ANOVA with Bonferroni’s multiple comparisons test for multiple comparisons. All analyses were performed in Prism (GraphPad Prism 10). Statistical significance was defined as *p* < 0.05.

## Supplementary information


Supplementary materials
Full and uncropped western blots


## Data Availability

The datasets used and/or analyzed during the current study are available from the corresponding author upon reasonable request.
