# Supplementary material for: Frontotemporal dementia associated CHCHD10V57E mutation aggravates tau pathology via disrupting the CHCHD10-Rab7A-TBC1D15 complex
Source: Cell Death Dis. 2026 Jun 19;17(1):762. doi: 10.1038/s41419-026-08983-9 (PMC13526787; doi:10.1038/s41419-026-08983-9)
Supplement: Supplementary file 1 [file 41419_2026_8983_MOESM1_ESM.pdf]

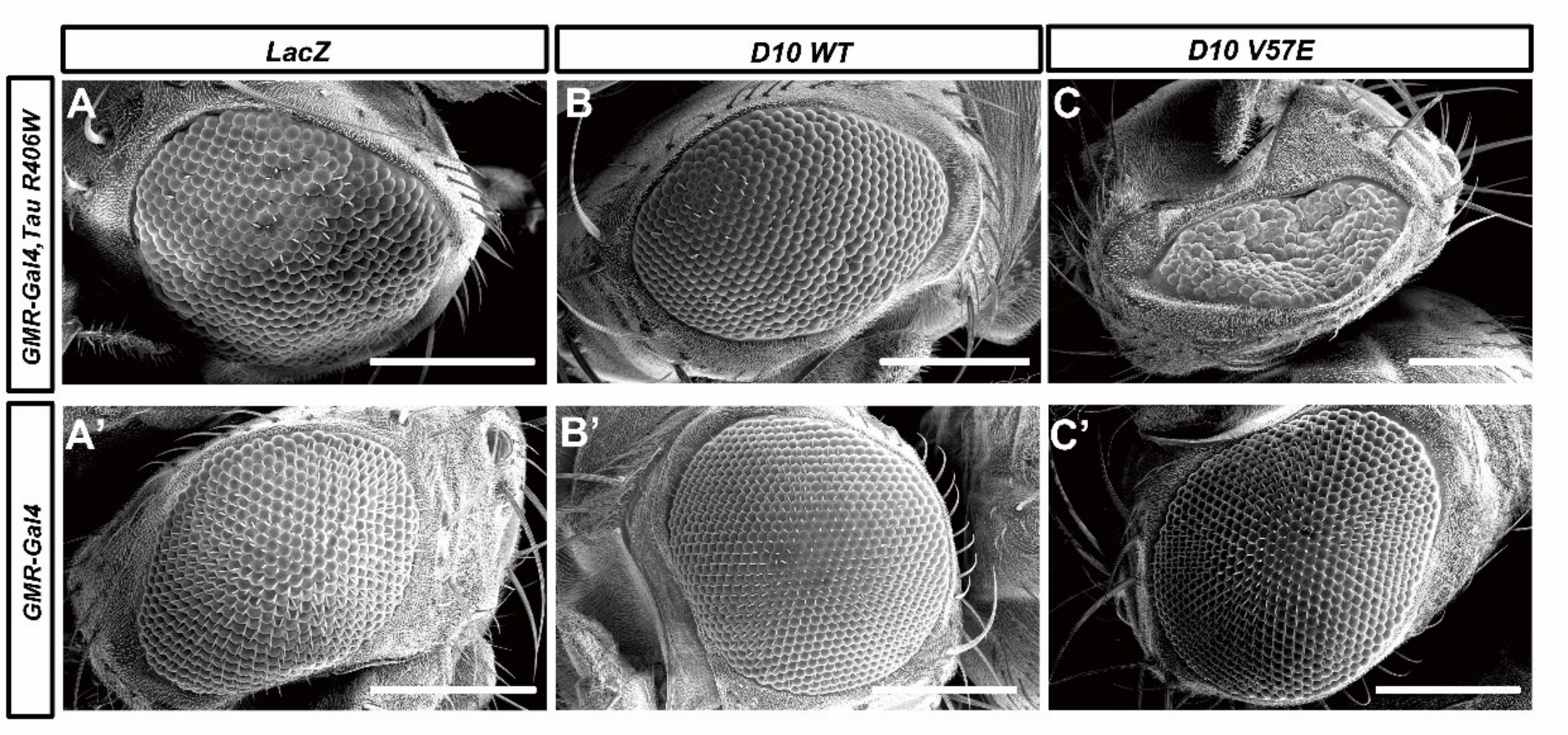

Figure S1. CHCHD10^{V57E} potentiates tau^{R406W}-induced retinal degeneration in *Drosophila*. (**A-C**, **A'-C'**) Expression of the indicated gene driven by *GMR-Gal4* in *Drosophila* eyes. Eye phenotypes were imaged using scanning electron microscopy (SEM). Scale bars: 200 μm.

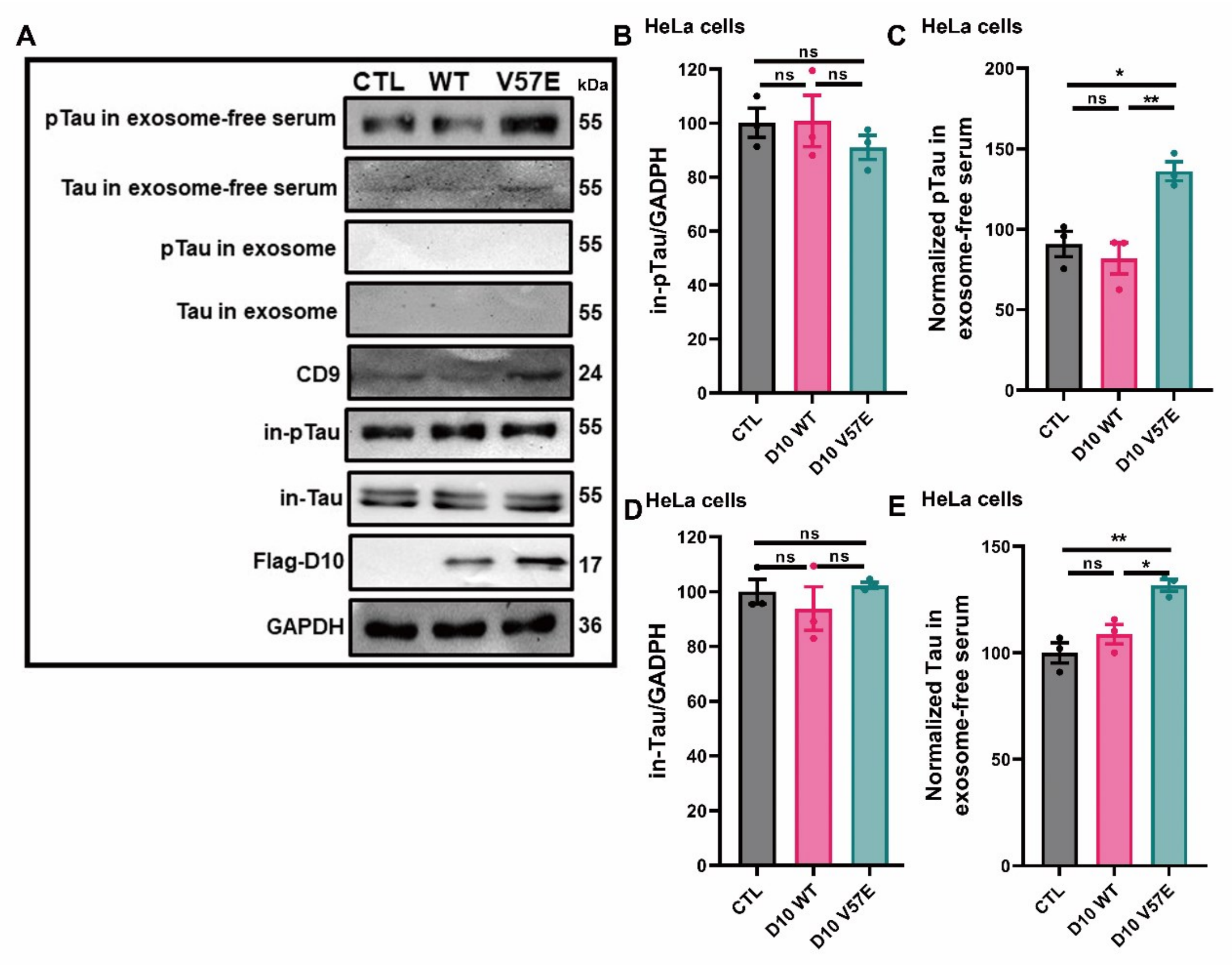

Figure S2. CHCHD10^{V57E}-induced pTau secretion is not via exosomes. (**A**) Cytosols, exosomes, and exosome-depleted cell media were collected from HeLa cells stably expressing tau-V5 that had been transfected with either *Flag-CHCHD10^{WT}*, *Flag-CHCHD10^{V57E}*, or empty vector constructs. Immunoblot analysis of intracellular pTau and total tau, pTau and total tau in exosomes and exosome-free serum were performed. GAPDH serves as a loading control. CD9 serves as an exosome marker. (**B**) Quantification of intracellular pTau in **A**. Intracellular pTau (in-pTau) levels normalized to GAPDH (one-way ANOVA with Bonferroni's multiple comparisons test, n=3, ns, non-significant). (**C**) Quantification of pTau in exosome-free serum in **A**. Phosphorylated tau levels in exosome-free serum calculated as (pTau in exosome-free serum)/(normalized intracellular pTau) (one-way ANOVA with Bonferroni's multiple comparisons test, n=3, * $p<0.05$; ** <0.001; ns, non-significant). (**D**) Quantification of

intracellular total tau in **A.** Intracellular total tau (in-Tau) levels normalized to GAPDH (one-way ANOVA with Bonferroni's multiple comparisons test, n=3, ns, non-significant). (**E**) Quantification of total tau in exosome-free serum in **A.** Total tau levels in exosome-free serum calculated as (total tau in exosome-free serum)/(normalized intracellular total tau) (one-way ANOVA with Bonferroni's multiple comparisons test, n=3, * <0.05; ** $p<0.01$; ns, non-significant).

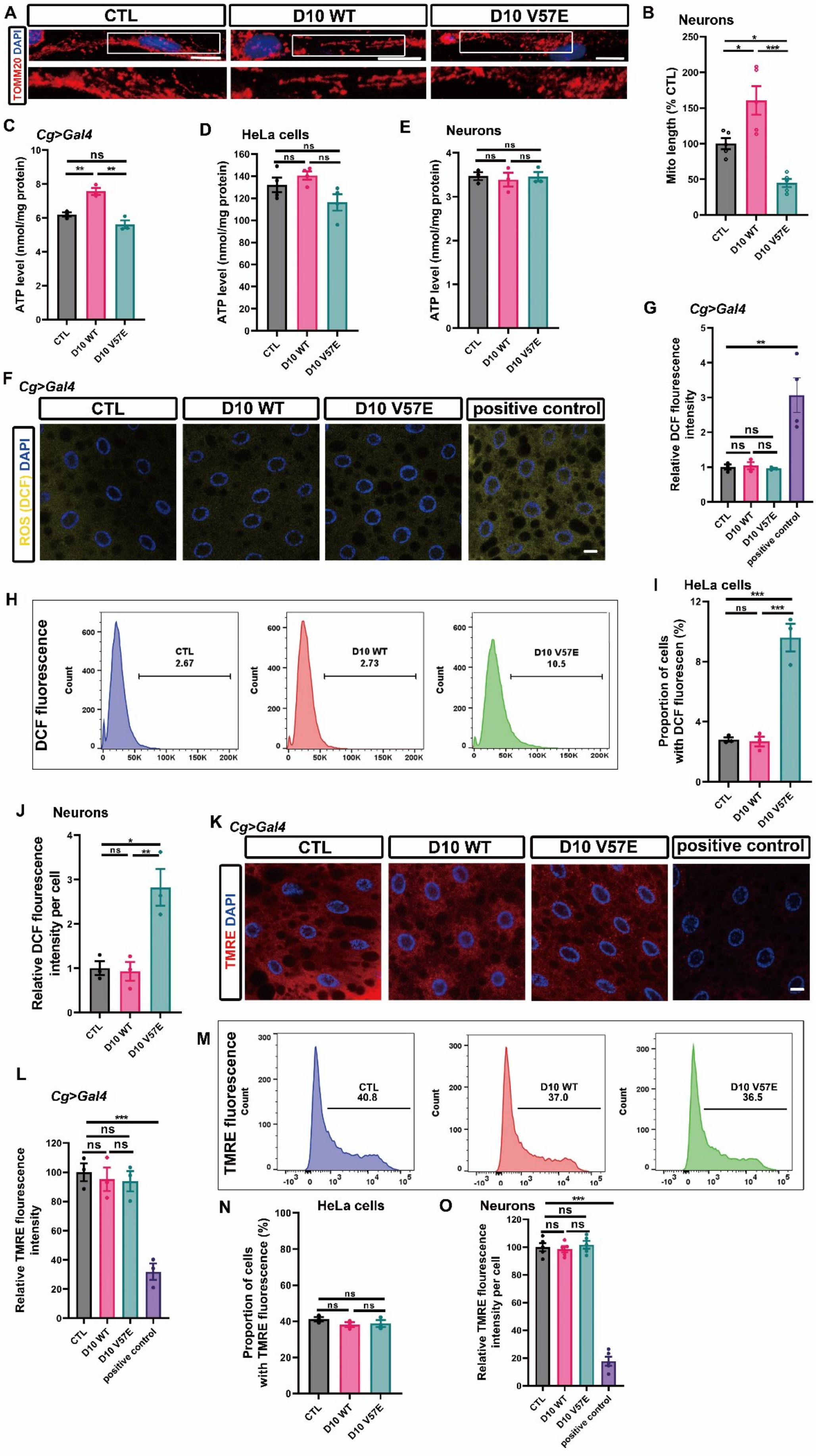

A
CTL
D10 WT
D10 V57E
TOMM20 DAPI
B
Neurons
Mito length (% CTL)
C
Cg>Gal4
ATP level (nmol/mg protein)
D
HeLa cells
ATP level (nmol/mg protein)
E
Neurons
ATP level (nmol/mg protein)
F
Cg>Gal4
positive control
ROS (DCF) DAPI
G
Cg>Gal4
Relative DCF flourescence intensity
H
DCF fluorescence
CTL 2.67
D10 WT 2.73
D10 V57E 10.5
Count
I
HeLa cells
Proportion of cells with DCF fluorescen (%)
J
Neurons
Relative DCF flourescence intensity per cell
K
Cg>Gal4
TMRE DAPI
L
Cg>Gal4
Relative TMRE flourescence intensity
M
TMRE fluorescence
CTL 40.8
D10 WT 37.0
D10 V57E 36.5
N
HeLa cells
Proportion of cells with TMRE fluorescence (%)
O
Neurons
Relative TMRE flourescence intensity per cell

Figure S3. CHCHD10^{V57E} overexpression induces mitochondrial defects. (**A-B**) Rat primary cortical neurons were transduced with CHCHD10^{WT}-HA, CHCHD10^{V57E}-HA or control lentivirus. (**A**) Neurons were stained with TOMM20 antibody (scale bar: 20 µm). (**B**) Mitochondrial length was quantified (one-way ANOVA with Bonferroni's multiple comparisons test, n=5, * p<0.05; *** p<0.001). (**C**) Flies carrying the *cg-Gal4* driver were crossed to *UAS-lacZ* (control), *UAS-CHCHD10^{WT}*, or *UAS-CHCHD10^{V57E}* flies. ATP levels in the fat body were measured and quantified (one-way ANOVA with Bonferroni's multiple comparisons test, n=3, ** p<0.01; ns, non-significant). (**D**) ATP levels in HeLa cells stably expressing *Flag-CHCHD10^{WT}*, *Flag-CHCHD10^{V57E}*, or empty vector were quantified (one-way ANOVA with Bonferroni's multiple comparisons test, n=4, ns, non-significant). (**E**) ATP levels of rat primary cortical neurons with the indicated genotypes were quantified (one-way ANOVA with Bonferroni's multiple comparisons test, n=3, ns, non-significant). (**F-G**) ROS levels in the fat body of the indicated genotypes were measured by DCF fluorescence and quantified (one-way ANOVA with Bonferroni's multiple comparisons test, n=3-4, ** p<0.01; ns, non-significant). Images in **F** were presented in pseudocolors to enhance accessibility. (**H-I**) ROS levels were measured by flow cytometry in HeLa cells expressing CHCHD10 variants using DCFH-HA staining and quantified (one-way ANOVA with Bonferroni's multiple comparisons test, n=3, ns, non-significant). (**J**) ROS levels in rat primary cortical neurons with the indicated genotypes were assessed by DCF fluorescence and quantified (one-way ANOVA with Bonferroni's multiple comparisons test, n=3, * p<0.05; ** p<0.01; ns, non-significant). (**K-L**) Mitochondrial membrane potential in fat

body cells with the indicated genotypes was measured using TMRE staining and quantified (one-way ANOVA with Bonferroni's multiple comparisons test, n=3, *** $p<0.001$; ns, non-significant). (**M**) Mitochondrial membrane potential was measured by flow cytometry in HeLa cells expressing CHCHD10 variants using TMRE staining. (**N**) Quantification of results in **M** (one-way ANOVA with Bonferroni's multiple comparisons test, n=3, ns, non-significant). (**O**) Mitochondrial membrane potential in rat primary cortical neurons with the indicated genotypes was assessed by TMRE fluorescence and quantified (one-way ANOVA with Bonferroni's multiple comparisons test, n=5, *** p<0.001, ns, non-significant).

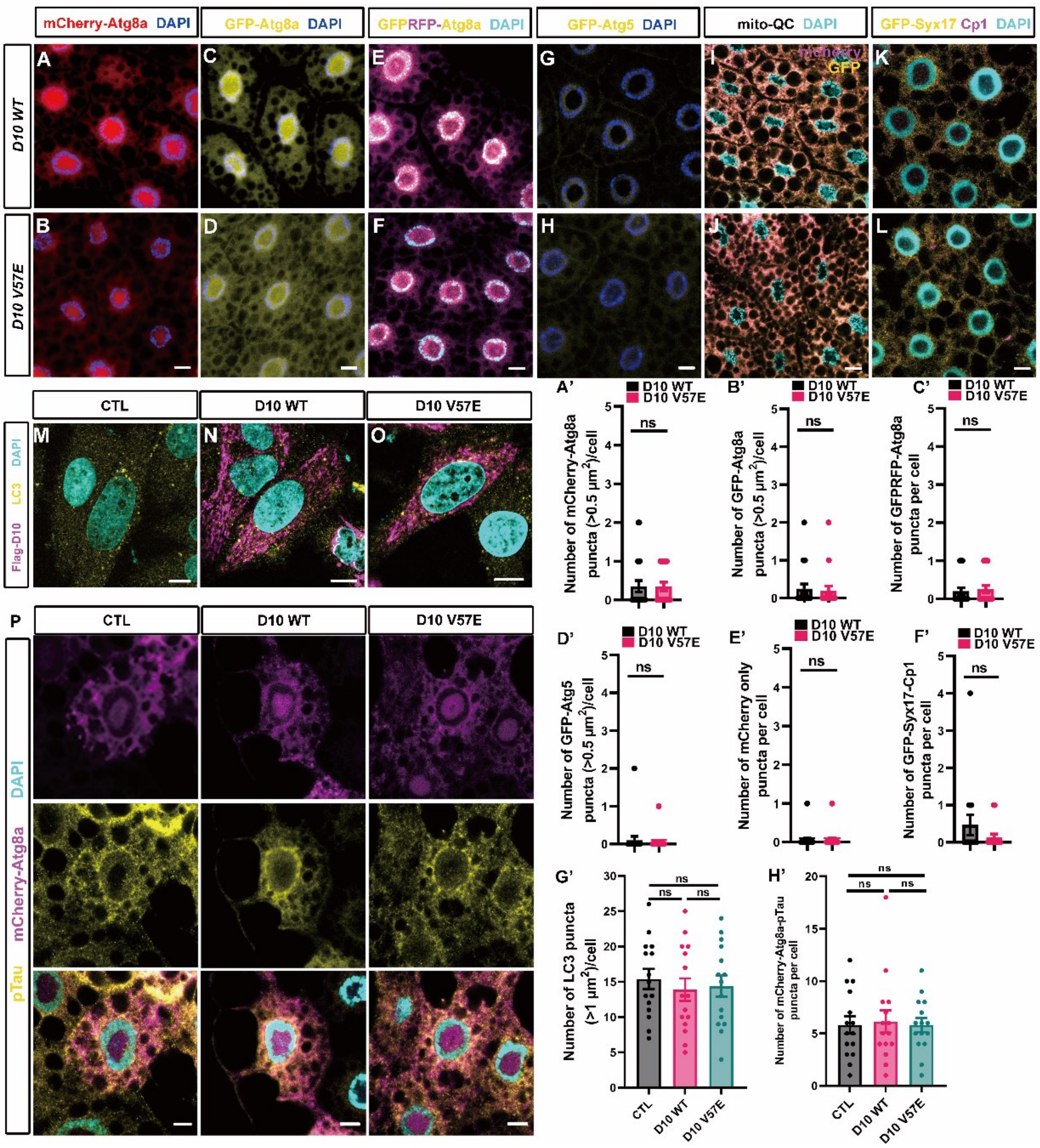

Figure S4. No significant changes were detected in the distribution of autophagy reporters between cells expressing CHCHD10^{WT} and CHCHD10^{V57E}. (**A-L**) Flies carrying the *cg-Gal4* driver were crossed to *UAS-CHCHD10^{WT}* or *UAS-CHCHD10^{V57E}* flies together with the various autophagy markers transgenic flies (*UAS-mCherry-Atg8a*, *UAS-GFP-Atg8a*, *UAS-GFPRFP-Atg8a*, *UAS-GFP-Atg5,* and *UAS-GFP-Syx17*) or mitophagy marker transgenic flies (*UAS-mito-QC*). Animals were dissected at the stage of third-instar larvae. The pattern of (**A-B**) mCherry-Atg8a, (**C-D**) GFP-Atg8a, (**E-F**) GFPRFP-Atg8a, (**G-H**) GFP-Atg5, (**I-J**) mito-QC, and (**K-L**) GFP-Syx17 with

lysosome marker Cp1 was examined by confocal microscopy. Scale bars: 10 μm. (**A'-F'**) Quantification of puncta for indicated markers (Student's t-test, n=15-20, ns, non-significant). (**M-O**) HeLa cells were transfected with *Flag-CHCHD10*^{WT}, *Flag-CHCHD10*^{V57E}, or empty vector. Violet signals indicate Flag-CHCHD10, yellow signals label LC3. Scale bars: 10 μm. (**G'**) Quantification of LC3 puncta per cell (one-way ANOVA with Bonferroni's multiple comparisons test, n=15, ns, non-significant). (**P**) Flies carrying the *cg-Gal4* driver were crossed to *UAS-lacZ* (control)*, UAS-CHCHD10*^{WT}*,* or *UAS-CHCHD10*^{V57E} flies together with the *UAS-Tau*^{R406W} and *UAS-mCherry-Atg8a* transgene. mCherry, anti-phosphorylated tau antibody, and DAPI were employed to label Atg8a, pTau, and nuclei, respectively. Scale bar: 10 μm. (**H'**) Quantification of mCherry-Atg8a-pTau puncta per cell (one-way ANOVA with Bonferroni's multiple comparisons test, n=15, ns, non-significant). Images in **C-P** were presented in pseudocolors to enhance accessibility.

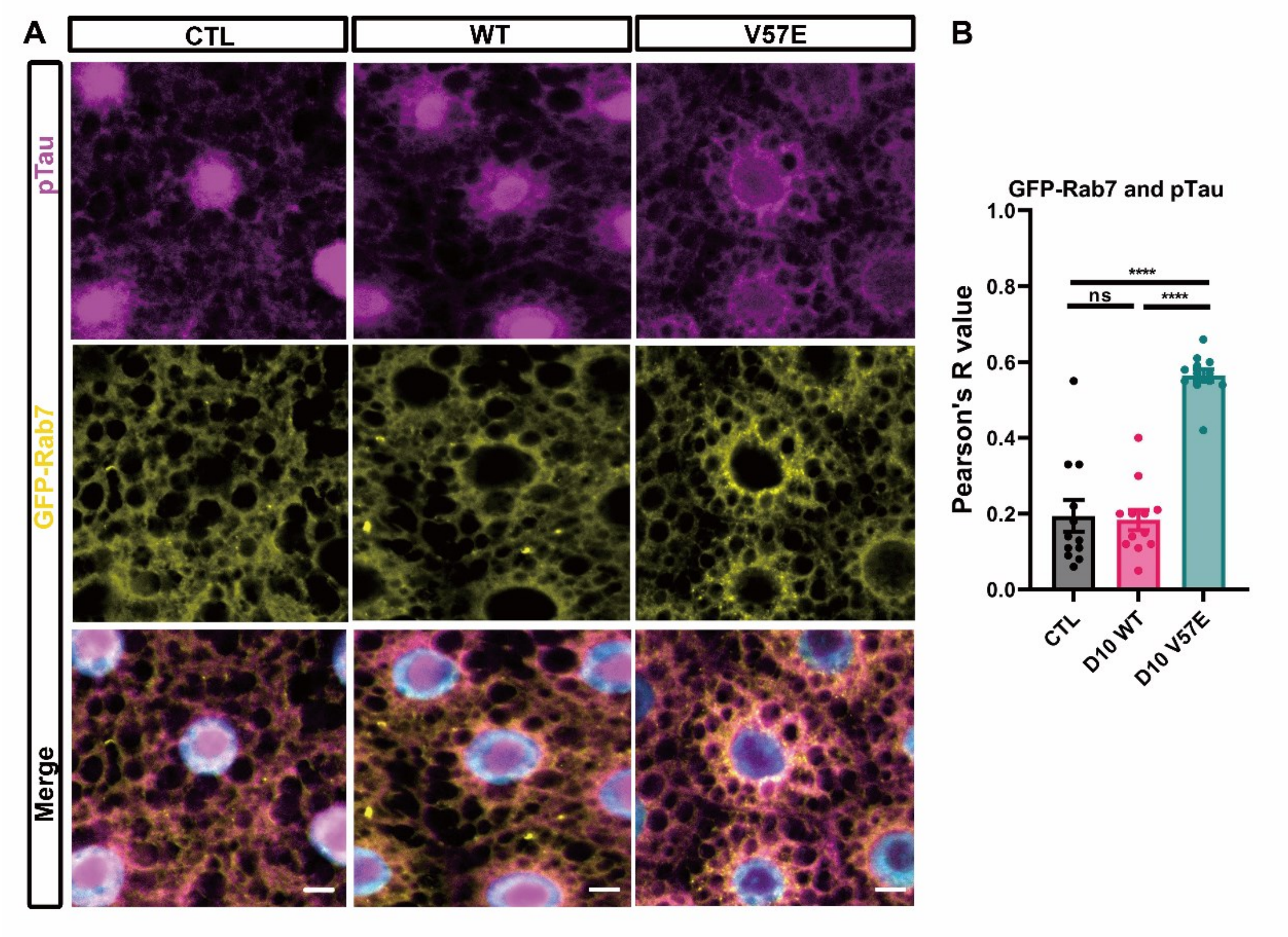

Figure S5. CHCHD10^{V57E} overexpression promotes pTau accumulation in Rab7A positive endo-lysosomal vesicles in *Drosophila*. (**A**) Flies carrying the *cg-Gal4* driver were crossed to *UAS-lacZ* (control), *UAS-CHCHD10^{WT}*, or *UAS-CHCHD10^{V57E}* flies together with the *UAS-Tau^{R406W}* and *UAS-GFP-Rab7* transgene. Animals were dissected at the stage of third-instar larvae. GFP, anti-phosphorylated tau antibody, and DAPI were employed to label Rab7, pTau, and nuclei, respectively. Images were presented in pseudocolors to enhance accessibility. Scale bars: 10 µm. (**B**) The Person's correlation between GFP-Rab7 and pTau was presented as mean ± SEM (one-way ANOVA with Bonferroni's multiple comparisons test, n=12, **** $p<0.0001$; ns, non-significant).

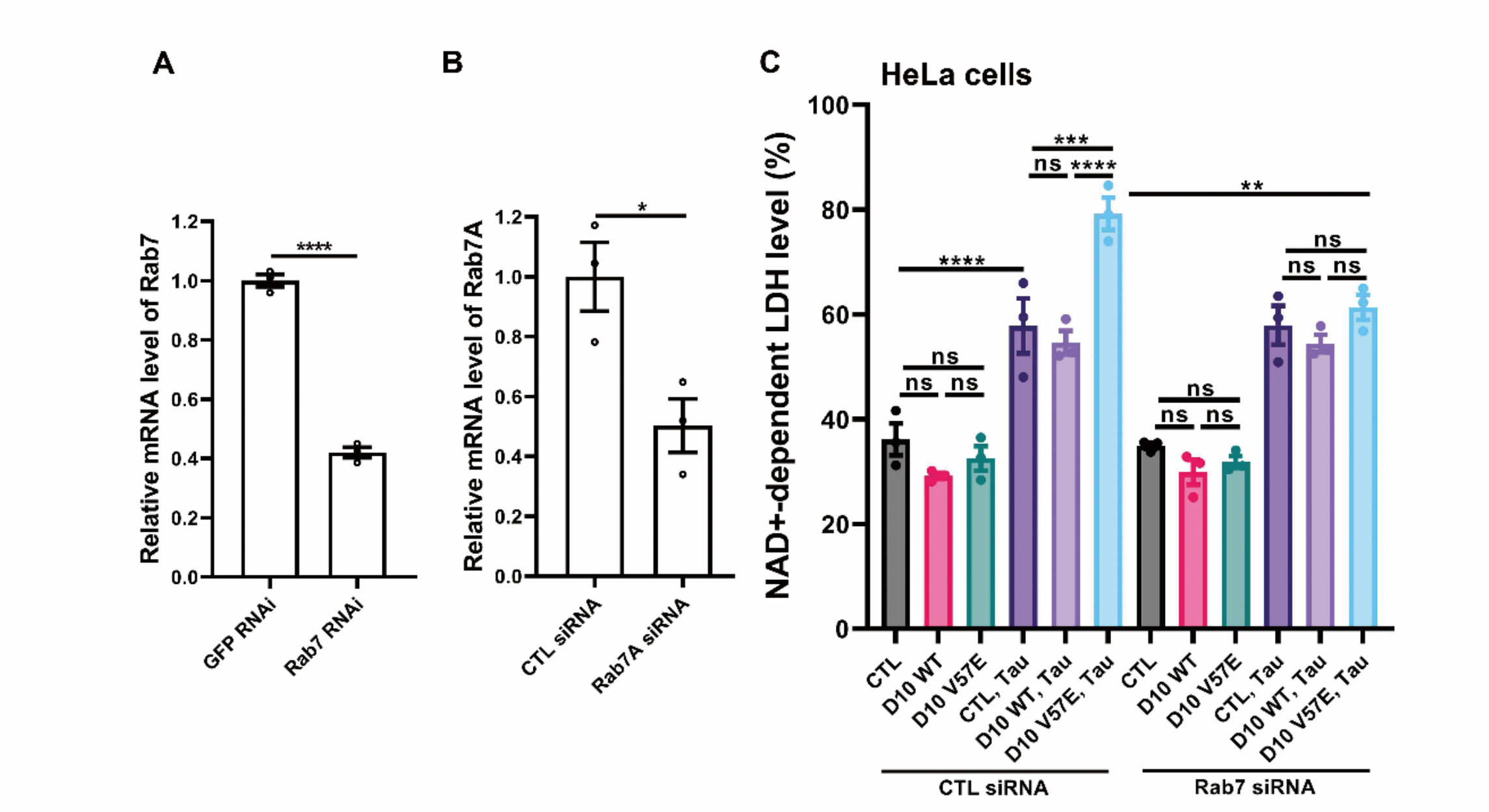

Figure S6. CHCHD10^{V57E} accelerates tau-related cellular toxicity through Rab7A. (**A**) Flies with *GMR-Gal4* driving *UAS-shGFP* or *UAS-shRab7* were collected 1-3 days post-eclosion. Rab7A mRNA levels in eyes were measured by RT-qPCR and quantified (Student's t-test, n = 3, **** p<0.0001). (**B**) HeLa cells expressing Tau-V5 were treated with control or *Rab7A* siRNA. Total RNA from cells was extracted and RT-qPCR was performed to quantify levels of Rab7A mRNA (Student's t-test, n = 3, * p<0.05). (**C**) Various Flag-CHCHD10 plasmids, together with control or *Rab7A* siRNA, were transfected into HeLa cells or HeLa cells stably expressing tau-V5. Cell culture media were subjected to NAD+-dependent D-lactate dehydrogenase detection (one-way ANOVA with Bonferroni's multiple comparisons test, n=3, ** p<0.01; *** p<0.001; **** p<0.0001; ns, non-significant).

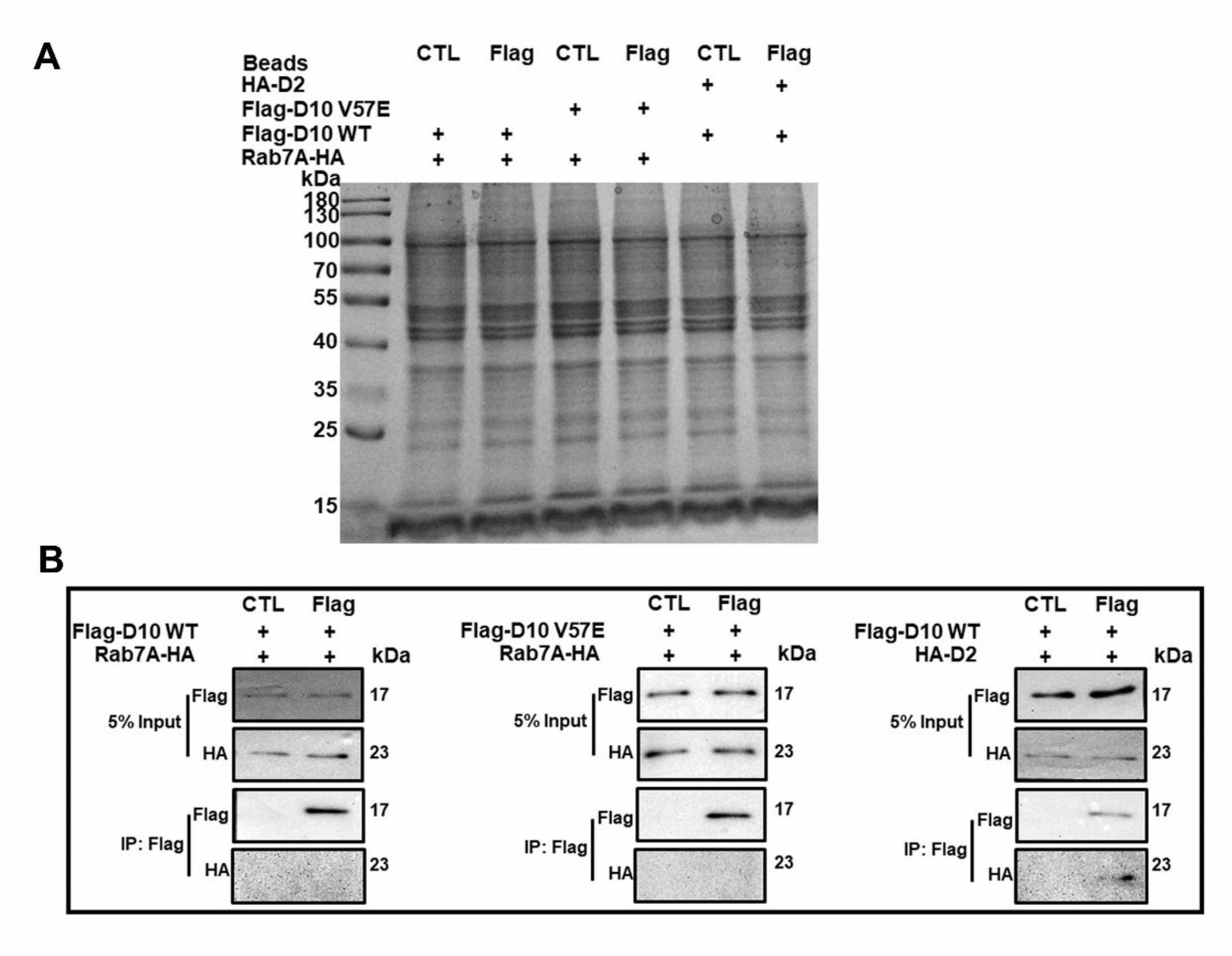

Figure S7. The immunoprecipitation assay failed to detect the Interaction between CHCHD10 variants and Rab7A. (**A-B**) Co-immunoprecipitation analysis of the interaction between Flag-CHCHD10^{WT} or Flag-CHCHD10^{V57E} and Rab7A-HA proteins. (**A**) 5% of cell lysates were analyzed by SDS-PAGE and Coomassie blue staining as a loading control. (**B**) Both 5% of cell lysates and immuno-purified HA and Flag complexes were analyzed by immunoblot. Co-IP analysis of Flag-CHCHD10^{WT} with HA-CHCHD2 was included as a positive control.

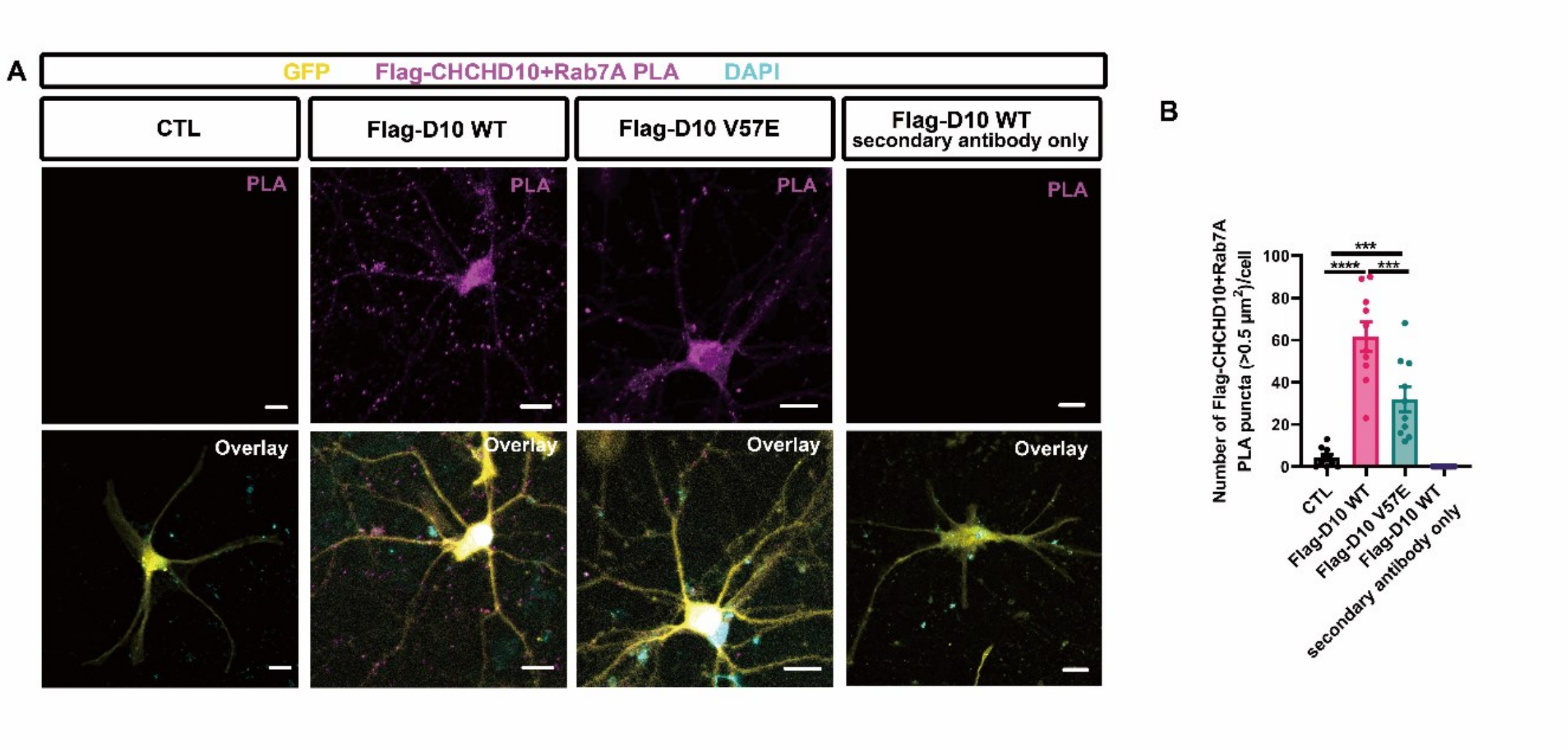

Figure S8. CHCHD10^{V57E} disrupts the proximity between CHCHD10 and Rab7A in rat primary cortical neurons. (**A**) Rat primary cortical neurons were transduced with lentivirus encoding a GFP reporter for CTL, CHCHD10^{WT}, and CHCHD10^{V57E}. PLA was performed to detect interactions between Flag-CHCHD10 and endogenous Rab7A (violet puncta). Scale bars: 20 μm. (**B**) Quantification of Flag-CHCHD10+Rab7A PLA puncta (>0.5 μm²) per cell in **A** (one-way ANOVA with Bonferroni's multiple comparisons test, n=10, *** p<0.001; **** p<0.0001; ns, non-significant).

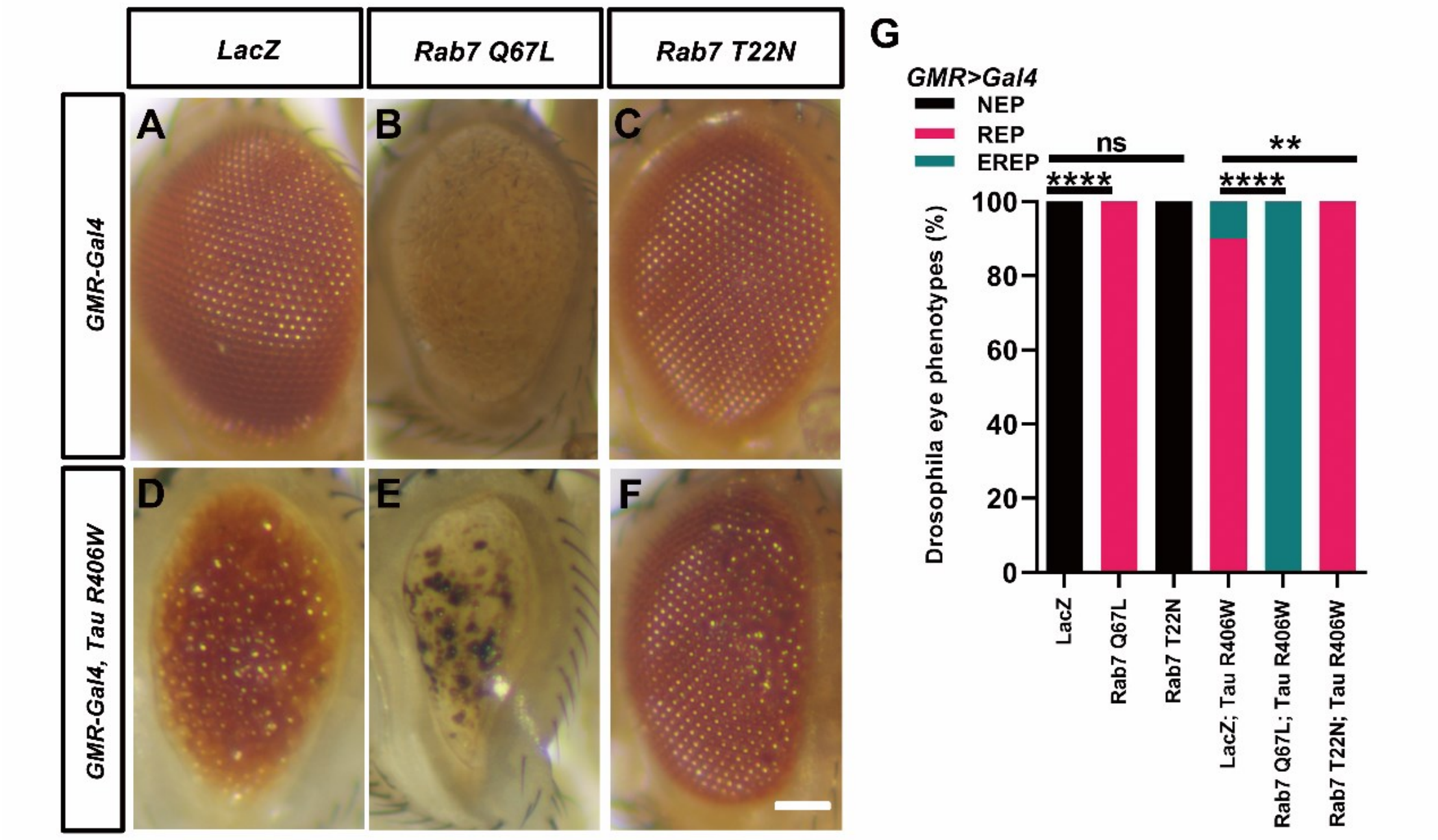

Figure S9. Tau^{R406W}-induced rough eye phenotypes are dependent on Rab7 GTPase activity. (**A-F**) Eye morphology of *Drosophila* with indicated genotypes imaged by stereoscopic microscopes (Scale bar: 100 μm). (**G**) Quantification of the eye phenotypes from flies with the indicated genotypes in **A-F** (Chi-square test, n=50, ** p<0.01; **** p<0.0001; ns, non-significant).

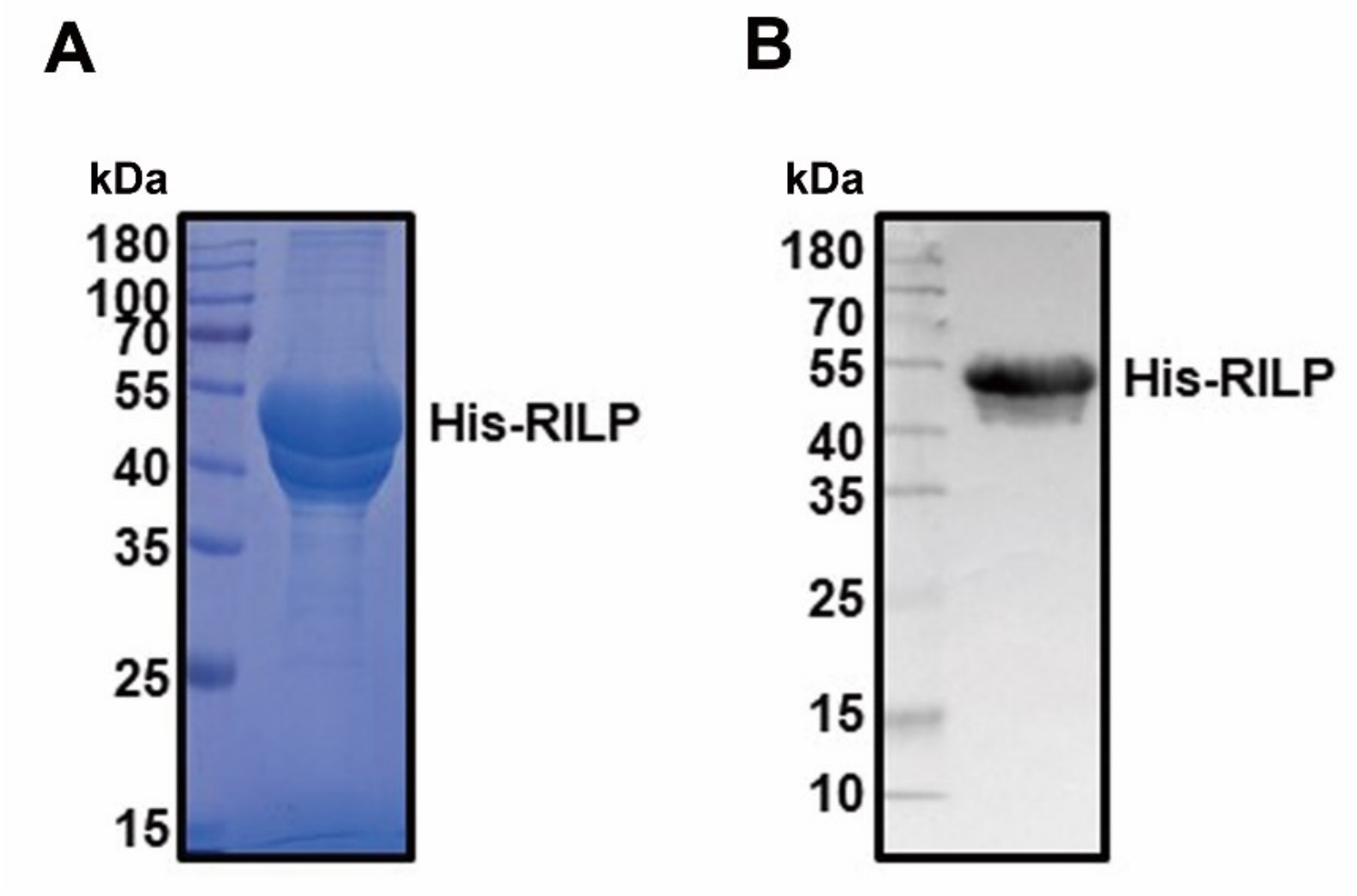

Figure S10. Purification of His-RILP protein. (**A-B**) Purification analysis of His-RILP from *Escherichia coli*: Coomassie blue-stained SDS-PAGE in **A** and anti-His immunoblot in **B**.

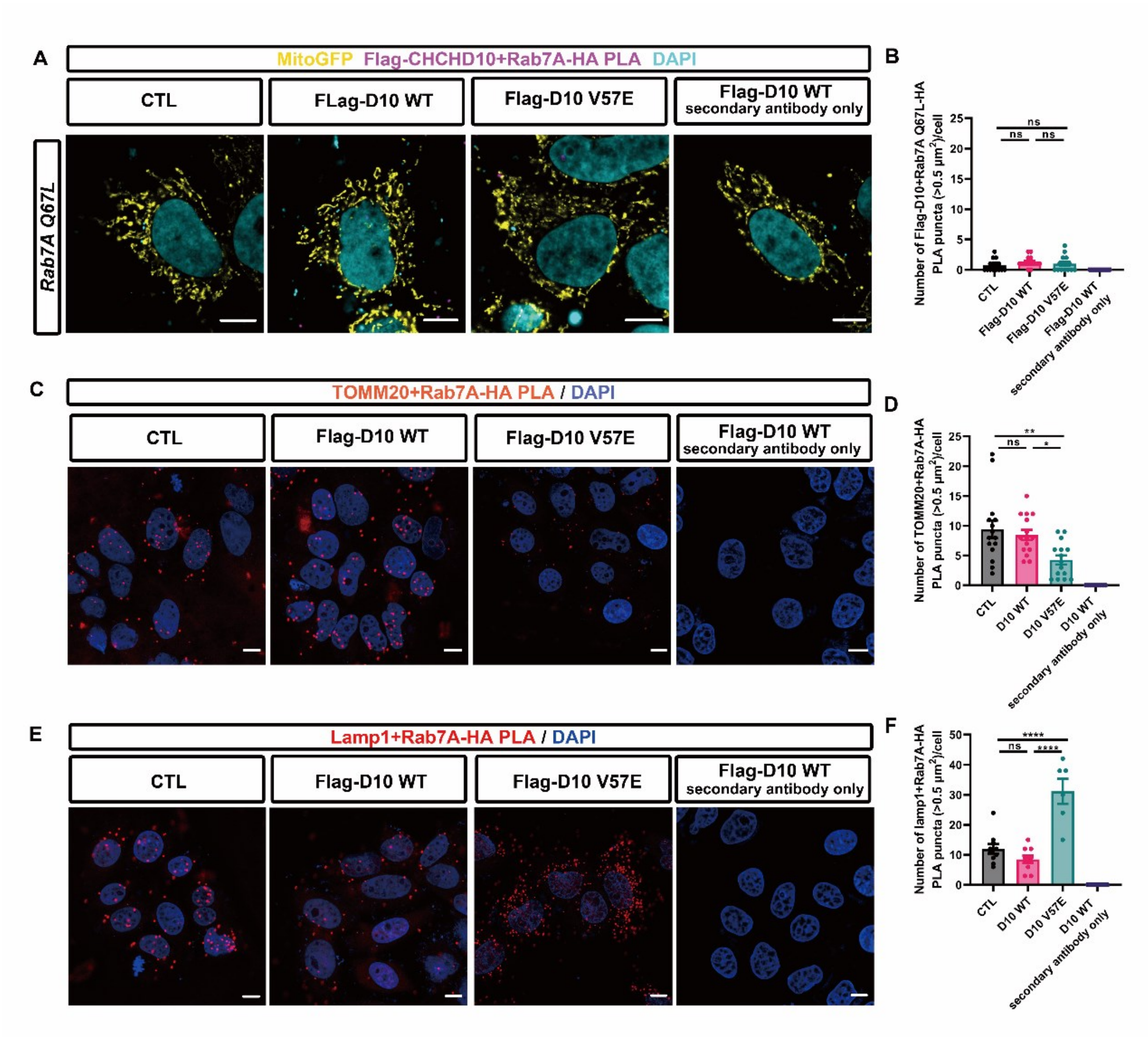

Figure S11. CHCHD10^{V57E} promotes dissociation of Rab7A from TOMM20 while enhancing Rab7A-Lamp1 proximity. (**A**) HeLa cells stably expressing Flag-CHCHD10^{WT}, Flag-CHCHD10^{V57E}, or control were transfected with *Rab7A^{Q67L}-HA* and *MitoGFP* constructs. Subsequently, the PLA was performed and the representative PLA images were shown. (**B**) Quantification of Flag-CHCHD10+ Rab7A^{Q67L}-HA PLA puncta count (>0.5 μm²) per cell in **A** (one-way ANOVA with Bonferroni's multiple comparisons test, n=15, ns, non-significant). (**C**) HeLa cells stably expressing Flag-CHCHD10^{WT}, Flag-CHCHD10^{V57E}, or control were transfected with Rab7-HA construct. Subsequently, the PLA was performed based on the anti-TOMM20 antibody and anti-HA antibody. Representative PLA images showing endogenous TOMM20 and Rab7A-

HA complexes (red puncta) in HeLa cells. Scale bars: 10 μm. (**D**) Quantification of TOMM20+Rab7A-HA PLA puncta count (>0.5 μm^2) per cell (one-way ANOVA with Bonferroni's multiple comparisons test, n=15, * p<0.05; ** p<0.01; ns, non-significant). (**E**) HeLa cells stably expressing *Flag-CHCHD10*^{WT}, *Flag-CHCHD10*^{V57E}, or empty vector (control) were transfected with Rab7-HA construct. Subsequently, the PLA was performed based on the anti-Lamp1 antibody and anti-HA antibody. Representative PLA images of endogenous Lamp1 and Rab7A-HA complex (red puncta) in HeLa cells. Scale bars: 10 μm. (**F**) Quantification of Lamp1+Rab7A-HA PLA puncta count (>0.5 μm^2) per cell (one-way ANOVA with Bonferroni's multiple comparisons test, n=6-10, **** p<0.01; ns, non-significant). Images in **A** was presented in pseudocolors to enhance accessibility.

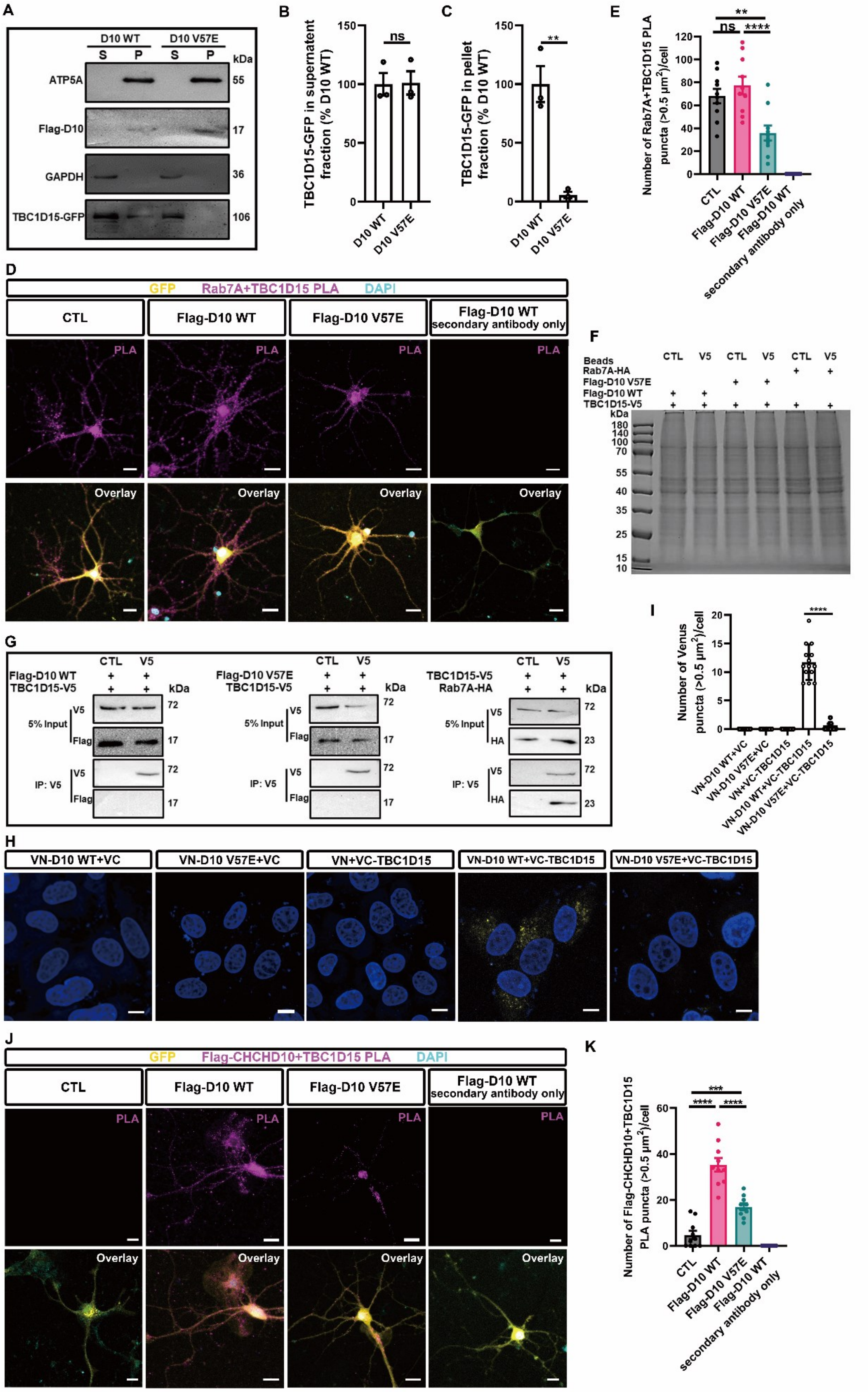

Figure S12. CHCHD10^{V57E} disrupts Rab7-TBC1D15 complex formation. (**A**) HeLa

cells stably expressing *Flag-CHCHD10^{WT}* or *Flag-CHCHD10^{V57E}* protein were transfected with TBC1D15-GFP construct. Subsequently, immunoblotting analysis of soluble (S) and mitochondrial (P) fractions was collected. Various antibodies were subjected to immunoblot. Molecular weights: ATP5A: 55 kDa, Flag-CHCHD10: 17 kDa, GAPDH: 36 kDa, TBC1D15-GFP: 106 kDa. (**B-C**) Quantification of TBC1D15-GFP levels in supernatant and pellet fractions in **A** (Student's t-test, n=3, ** $p<0.01$; ns, non-significant). (**D**) Rat primary cortical neurons were transduced with lentivirus encoding a GFP reporter for CTL, Flag-CHCHD10^{WT} and Flag-CHCHD10^{V57E}. PLA was performed to detect interactions between endogenous Rab7A and TBC1D15. Scale bars: 20 μm. (**E**) Quantification of Rab7A+TBC1D15 PLA puncta (>0.5 μm²) per cell in **D** (one-way ANOVA with Bonferroni's multiple comparisons test, n=10, ** p<0.01; **** $p<0.0001$; ns, non-significant). (**F-G**) Co-immunoprecipitation analysis of the interaction between Flag-CHCHD10^{WT} or Flag-CHCHD10^{V57E} and TBC1D15-V5 proteins. (**F**) 5% of cell lysates were analyzed by SDS-PAGE and Coomassie blue staining as a loading control. (**G**) Both 5% of cell lysates and immuno-purified V5 and Flag complexes were analyzed by immunoblot. Co-IP analysis of Rab7A-HA with TBC1D15-V5 was included as a positive control. (**H**) Venus fluorescent images with the co-expression of indicated plasmids. Scale bars: 10 μm. (**I**) Quantification of Venus puncta count (>0.5 μm²) per cell (Student's t-test, n=14, **** $p<0.0001$). (**J-K**) Rat primary cortical neurons were transduced with lentivirus encoding a GFP reporter for CTL, Flag-CHCHD10^{WT} and Flag-CHCHD10^{V57E}. PLA was performed to detect interactions between Flag-CHCHD10 and endogenous TBC1D15 (violet puncta).

Scale bars: 20 μm. (**K**) Quantification of Flag-CHCHD10+TBC1D15 PLA puncta (>0.5 μm^2) per cell in **J** (one-way ANOVA with Bonferroni's multiple comparisons test, n=10, *** $p<0.001$; **** $p<0.0001$). Images in **D** and **J** were presented in pseudocolors to enhance accessibility.

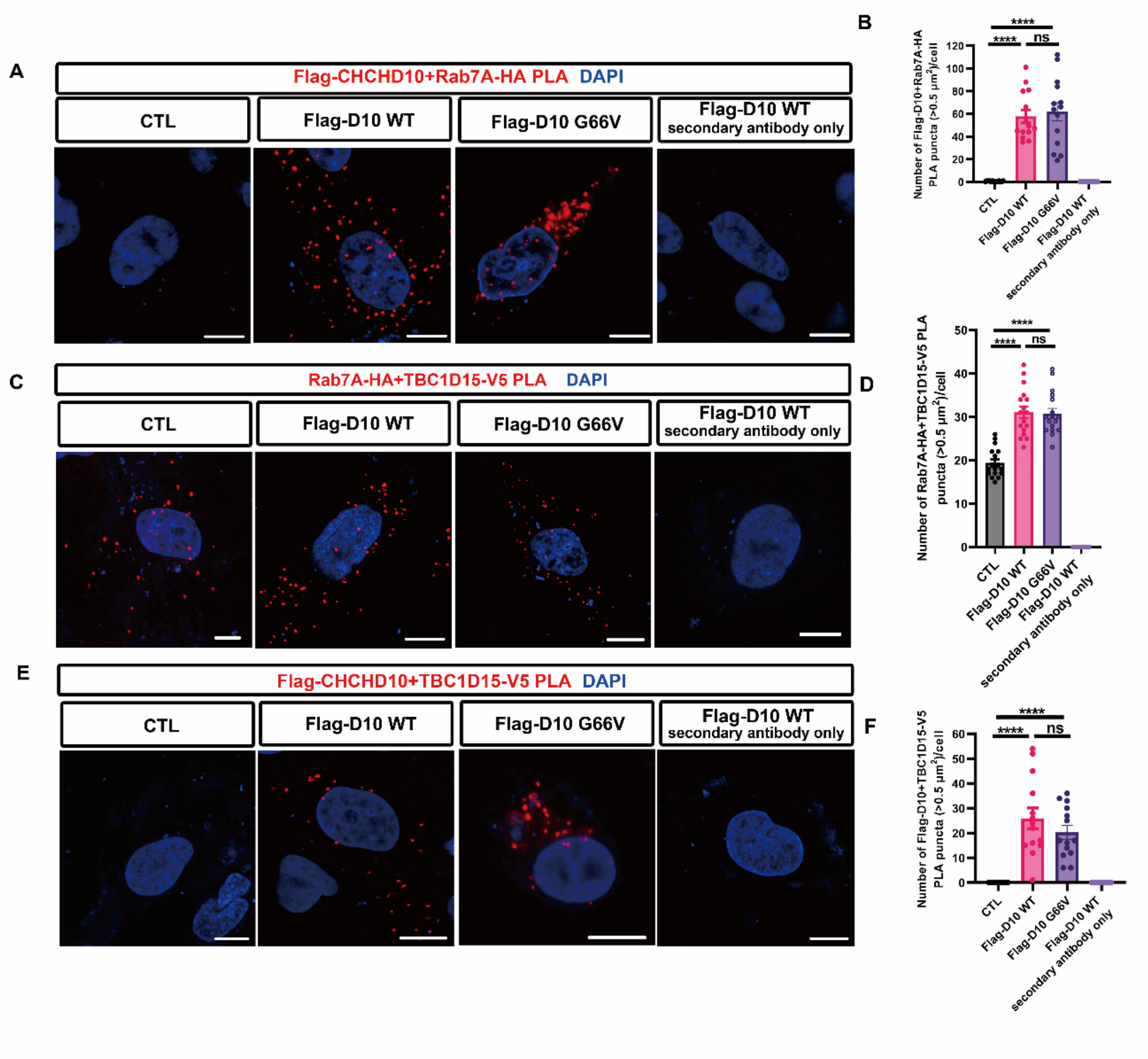

Figure S13. G66V mutation of CHCHD10 does not alter CHCHD10's proximity with Rab7-TBC1D15. (**A**) Representative PLA images showing the interaction between Flag-CHCHD10 variants and Rab7A-HA complex (red puncta) in HeLa cells. (**B**) Quantification of Flag-CHCHD10+Rab7A-HA PLA puncta (>0.5 μm²) per cell in **A** (one-way ANOVA with Bonferroni's multiple comparisons test, n=14, **** *p*<0.0001; ns, non-significant). (**C**) Representative PLA images showing the interaction between Rab7A-HA and TBC1D15-V5 in HeLa cells. (**D**) Quantification of Rab7A-HA+TBC1D15-V5 PLA puncta (one-way ANOVA with Bonferroni's multiple comparisons test, n=17, **** *p*<0.0001; ns, non-significant). (**E**) Representative PLA

images showing the interaction between Flag-CHCHD10 and TBC1D15-V5 in HeLa cells. (**F**) Quantification of Flag-CHCHD10+TBC1D15-V5 PLA puncta (one-way ANOVA with Bonferroni's multiple comparisons test, n=14, **** $p<0.0001$; ns, non-significant). Scale bars: 10 μm.

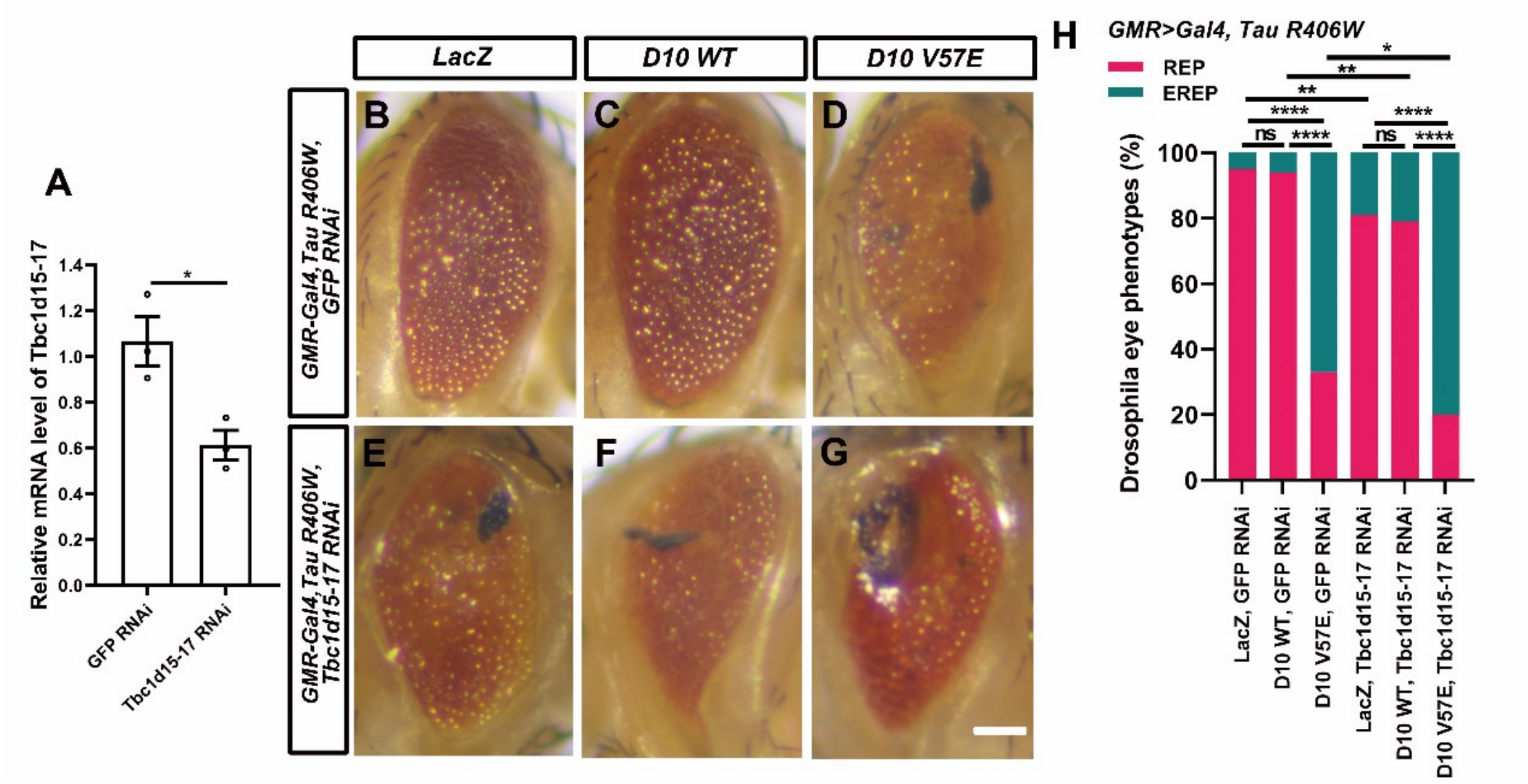

Figure S14. *TBC1D15* knocking down aggravates Tau^{R406W}-induced eye defects in *Drosophila*. (**A**) Flies with *GMR-Gal4* driving *UAS-shGFP* or *UAS-shTbc1d15-17* were collected 1-3 days post-eclosion. Tbc1d15-17 mRNA levels in eyes were measured by RT-qPCR and quantified (Student's t-test, n = 3, * p<0.05). (**B-G**) Eye morphology of *Drosophila* with indicated genotypes imaged by stereoscopic microscopes (Scale bar: 100 μm). (**H**) Quantification of the eye phenotypes from flies with the indicated genotypes (Chi-square test, n=50, * p<0.05; ** p<0.01; **** p<0.0001; ns, non-significant).

Fly genetics:

The genotypes of the fly strains generated in the paper are as followings:

Figure 1B: UAS-LacZ/GMR-Gal4; UAS-Tau^{R406W}/+. **B':** UAS-LacZ//GMR-Gal4. **C:** UAS-CHCHD10^{WT}-3HA/GMR-Gal4; UAS-Tau^{R406W}/+. **C':** UAS-CHCHD10^{WT}-3HA/GMR-Gal4. **D:** UAS-CHCHD10^{V57E}-3HA/GMR-Gal4; UAS-Tau^{R406W}/+. **D':** UAS-CHCHD10^{V57E}-3HA/GMR-Gal4. **E:** UAS-CHCHD10^{G66V}-3HA/GMR-Gal4; UAS-Tau^{R406W}/+. **E':** UAS-CHCHD10^{G66V}-3HA/GMR-Gal4. **H-I:** Elav-Gal4/UAS-LacZ. Elav-Gal4/UAS-CHCHD10^{WT}-3HA. Elav-Gal4/UAS-CHCHD10^{V57E}-3HA. Elav-Gal4/UAS-LacZ; UAS-Tau^{R406W}/+. Elav-Gal4/UAS-CHCHD10^{WT}-3HA; UAS-Tau^{R406W}/+. Elav-Gal4/UAS-CHCHD10^{V57E}-3HA; UAS-Tau^{R406W}/+.

Figure 3A: UAS-CHCHD10^{WT}-3HA/Cg-Gal4MitoGFP. UAS-CHCHD10^{V57E}-3HA/Cg-Gal4MitoGFP.

Figure 4A-A': UAS-LacZ/Cg-Gal4Rab5GFP. **B-B':** UAS-CHCHD10^{WT}-3HA/Cg-Gal4Rab5GFP. **C-C':** UAS-CHCHD10^{V57E}-3HA/Cg-Gal4Rab5GFP. **D-D':** UAS-LacZ/Cg-Gal4Rab7GFP. **E-E':** UAS-CHCHD10^{WT}-3HA/Cg-Gal4Rab7GFP. **F-F':** UAS-CHCHD10^{V57E}-3HA/Cg-Gal4Rab7GFP. **G-G':** UAS-LacZ/Cg-Gal4Lamp1GFP. **H-H':** UAS-CHCHD10^{WT}-3HA/Cg-Gal4Lamp1GFP. **I-I':** UAS-CHCHD10^{V57E}-3HA/Cg-Gal4Lamp1GFP.

Figure 5A: UAS-LacZ/GMR-Gal4; UAS-Tau^{R406W}/UAS-GFP RNAi. **B:** UAS-CHCHD10^{WT}-3HA/GMR-Gal4; UAS-Tau^{R406W}/UAS-GFP RNAi. **C:** UAS-CHCHD10^{V57E}-3HA/GMR-Gal4; UAS-Tau^{R406W}/UAS-GFP RNAi. **D:** UAS-LacZ/GMR-Gal4; UAS-Tau^{R406W}/UAS-Rab7 RNAi. **E:** UAS-CHCHD10^{WT}-3HA/GMR-Gal4; UAS-Tau^{R406W}/UAS-Rab7 RNAi. **F:** UAS-CHCHD10^{V57E}-3HA/GMR-Gal4; UAS-Tau^{R406W}/UAS-Rab7 RNAi. **H-I:** Elav-Gal4/UAS-LacZ; UAS-Tau^{R406W}/UAS-GFP RNAi. Elav-Gal4/UAS-CHCHD10^{WT}-3HA; UAS-Tau^{R406W}/UAS-GFP RNAi. Elav-Gal4/UAS-CHCHD10^{V57E}-3HA; UAS-Tau^{R406W}/UAS-GFP RNAi. Elav-Gal4/UAS-LacZ; UAS-Tau^{R406W}/UAS-Rab7 RNAi. Elav-Gal4/UAS-CHCHD10^{WT}-3HA; UAS-Tau^{R406W}/UAS-Rab7 RNAi. Elav-Gal4/UAS-CHCHD10^{V57E}-3HA; UAS-Tau^{R406W}/UAS-Rab7 RNAi.

Figure S1A: UAS-LacZ/GMR-Gal4; UAS-Tau^{R406W}/+. **A':** UAS-LacZ/GMR-Gal4. **B:** UAS-CHCHD10^{WT}-3HA/GMR-Gal4; UAS-Tau^{R406W}/+. **B':** UAS-CHCHD10^{WT}-3HA/GMR-Gal4. **C:** UAS-CHCHD10^{V57E}-3HA/GMR-Gal4; UAS-Tau^{R406W}/+. **C':** UAS-CHCHD10^{V57E}-3HA/GMR-Gal4.

Figure S3C,3F-G,3K-L: UAS-LacZ/Cg-Gal4. UAS-CHCHD10^{WT}-3HA/Cg-Gal4. UAS-CHCHD10^{V57E}-3HA/Cg-Gal4.

Figure S4A: UAS-CHCHD10^{WT-}3HA/Cg-Gal4Atg8amCherry. **B:** UAS-CHCHD10^{V57E}-3HA/Cg-Gal4Atg8amCherry. **C:** UAS-CHCHD10^{WT}-3HA/Cg-Gal4Atg8aGFP. **D:** UAS-CHCHD10^{V57E}-3HA/Cg-Gal4Atg8aGFP. **E:** UAS-CHCHD10^{WT}-3HA/Cg-Gal4Atg8aGFPRFP. **F:** UAS-CHCHD10^{V57E}-3HA/Cg-Gal4Atg8aGFPRFP. **G:** UAS-

CHCHD10^{WT}-3HA/Cg-Gal4Atg5GFP. **H:** *UAS-CHCHD10^{V57E}-3HA/Cg-Gal4Atg5GFP.* **I:** *UAS-CHCHD10^{WT}-3HA/Cg-Gal4; mito-QC/+.* **J:** *UAS-CHCHD10^{V57E}-3HA/Cg-Gal4; mito-QC/+.* K**:** *UAS-CHCHD10^{WT}-3HA/Cg-Gal4; UAS-Syx17GFP/+.* **L:** *UAS-CHCHD10^{V57E}-3HA/Cg-Gal4; UAS-Syx17GFP/+.***P:** *UAS-LacZ/Cg-Gal4mCherryAtg8a; UAS-Tau^{R406W}/+. UAS-CHCHD10^{WT}-3HA/Cg-Gal4mCherryAtg8a; UAS-Tau^{R406W}/+. UAS-CHCHD10^{V57E}-3HA/Cg-Gal4mCherryAtg8a; UAS-Tau^{R406W}/+.*

Figure S5A: *UAS-LacZ/Cg-Gal4Rab7GFP; UAS-Tau^{R406W}/+. UAS-CHCHD10^{WT}-3HA/Cg-Gal4Rab7GFP; UAS-Tau^{R406W}/+. UAS-CHCHD10^{V57E}-3HA/Cg-Gal4Rab7GFP; UAS-Tau^{R406W}/+.*

Figure S6A: *GMR-Gal4/+; UAS-GFP RNAi/+. GMR-Gal4/+; UAS-Rab7 RNAi/+.*

Figure S9A: *UAS-LacZ/GMR-Gal4.* **B:** *UAS-Rab7^{Q67L}/GMR-Gal4.* **C:** *+/GMR-Gal4; UAS-Rab7^{T22N}-V5/+.* **D:** *UAS-LacZ/GMR-Gal4; UAS-Tau^{R406W}/+.* **E:** *UAS-Rab7^{Q67L}/GMR-Gal4; UAS-Tau^{R406W}/+.* **F:** *+/GMR-Gal4; UAS-Tau^{R406W}/UAS-Rab7^{T22N}-V5.*

Figure S14B: *UAS-LacZ/GMR-Gal4; UAS-Tau^{R406W}/UAS-GFP RNAi.* **C:** *UAS-CHCHD10^{WT}-3HA/GMR-Gal4; UAS-Tau^{R406W}/UAS-GFP RNAi.* **D:** *UAS-CHCHD10^{V57E}-3HA/GMR-Gal4; UAS-Tau^{R406W}/UAS-GFP RNAi.* **E:** *UAS-LacZ/GMR-Gal4; UAS-Tau^{R406W}/UAS-Tbc1d15-17 RNAi.* **F:** *UAS-CHCHD10^{WT}-3HA/GMR-Gal4; UAS-Tau^{R406W}/UAS-Tbc1d15-17 RNAi.* **G:** *UAS-CHCHD10^{V57E}-3HA/GMR-Gal4; UAS-Tau^{R406W}/UAS-Tbc1d15-17 RNAi.*

Lists of fly stocks and constructs

Drosophila stocks

Stock description	Stock number	Flybase ID	Source
w[1118]; P{w[+mC]=Cg-GAL4.A}2	7011	FBst0007011	Bloomington Drosophila Stock Center
w[*];P{GAL4-ninaE.GMR}12	1104	FBti0002994	Bloomington Drosophila Stock Center
P{w[+mC]=GAL4-elav.L}2/CyO	8765	FBst0008765	Bloomington Drosophila Stock Center
UAS-LacZ	1776		Bloomington Drosophila Stock Center
UAS-mitoQC	91641		Bloomington Drosophila Stock Center
UAS-Rab7 Q67L	42707		Bloomington Drosophila Stock Center
UAS-Rab7 RNAi	THU2437		Tsinghua Fly Center
UAS- Tbc1d15-17 RNAi	THU0704		Tsinghua Fly Center
UAS-GFP RNAi	N/A		R. Zhou (Johns Hopkins All Children Hospital, St. Petes, FL)
UAS-Tau R406W	BCF588		Center for Excellence in Molecular Cell Science
Cg-gal4 UAS-GFP-Lamp1	N/A		'C. Tong (Zhejiang University, Hangzhou, China)
Cg-gal4 UAS-mCherry-Atg8a	N/A		C. Tong (Zhejiang University, Hangzhou, China)
Cg-gal4 UAS-GFP-Atg8a	N/A		C. Tong (Zhejiang University, Hangzhou, China)
Cg-gal4 UAS-GFPRFP-Atg8a	N/A		C. Tong (Zhejiang University, Hangzhou, China)
Cg-gal4 UAS-GFP-Atg5	N/A		C. Tong (Zhejiang University, Hangzhou, China)
Cg-gal4 UAS-GFP-Syx17	N/A		C. Tong (Zhejiang University, Hangzhou, China)
Cg-gal4 UAS-GFP-Rab5	N/A		C. Tong (Zhejiang University, Hangzhou, China)
Cg-gal4 UAS-GFP-Rab7	N/A		C. Tong (Zhejiang University, Hangzhou, China)
Cg-gal4 UAS-MitoGFP	N/A		C. Tong (Zhejiang University, Hangzhou, China)

Transgene flies list

Transgene vectors	Insertion site	Verified
pUAST-attB-CHCHD10 WT-HA	75B1	Yes
pUAST-attB-CHCHD10 V57E-HA	75B1	Yes
pUAST-attB-CHCHD10 G66V-HA	75B1	Yes
pUAST-attB-Rab7 T22N-V5	25C6	Yes

Table 1 Primers used for plasmids construction

Constructs	Forward primers	Reverse primers
pCMV-tau-v5-IRES-puro	5'-aagtcgacatggctgagccccgcca-3'	5'-cggaattctcacgtagaatcgagaccgaggagagggttagggataggcttacccaaaccctgcttggccagggag-3'
pLV2-EF1a-Tau-v5-P2A-GFP	5'-tttcaggtgtcgtga cgtacg atggctgagccccgccaggagttc-3'	5'-gagaagttagtggctccggatcccgtagaatcgagaccgaggagagggtt-3'
pLV2-EF1a-CHCHD10 WT-3HA-P2A-mcherry	5'-tttcaggtgtcgtga cgtacg atgcctcggggaagccgcagc-3'	5'-tggcccgggattctcttcgacatcccctgcttgtttcaacagggagaagttagtggctccggatccatgtggtaacgcgctag-3'
pcdna3.1-Rab7A-HA	5'-cccaagcttatgacctctaggaagaaagt-3'	5'-ccctctagatcaagcgtaatctggaacatcgtatgggtagcaactgcagctttctgccg-3'
pcdna3.1-Rab5-HA	5'-cccaagcttatggcgggtcggggaggcgc-3'	5'-ccctctagatcaagcgtaatctggaacatcgtatgggtagttgctgcagcactggctcc-3'
pCMV-Flag-CHCHD10 WT-IRES-puro	5'-acgcgtcgacatgcctcggggaagccgcag-3'	5'-ggaattctcacttgtcatcgtcatccttgtagtcgggcagggagctcagaccat-3'
pUAS-attB-CHCHD10 WT-3HA	5'-ggaattcatgcctcggggaagccgcag-3'	5'-ggggtaccgggcagggagctcagaccat-3'
p3x-Flag-CHCHD10 WT	5'-cgaagcttatgcctcggggaagccgcagcg-3'	5'-cggtcgactcagggcagggagctcagaccgta-3'
pBiFC-Flag-CHCHD10 WT-VN173	5'-caaagacgatgacgacaagcttatgcctcggggaagc-3'	5'-cctcgcccttgctcagaattcgcgggcagggagctc-3'
pBiFC-HA-Rab7A-VC155	5'-ggccatggaggcccgaattcggatgacctctaggaag-3'	5'-cacgccggacgggtacctcgagagcaactgcagctttc-3'
pBiFC-HA-TBC1D15-VC155	5'-ggccatggaggcccgaattcggatggcggcggcgggtg-3'	5'-cacgccggacgggtacctcgagatgcaggtgttaatctg-3'
CHCHD10 V57E	5'-acggccgcaggggtagccgagggctcggctgtgggacac-3'	5'-gtgtcccacagccgagccctcggctacccctgcggccgt-3'
Rab7A Q67L	5'-cacagcaggactggaacggttcc-3'	5'-ggaaccgttccagtcctgctgtg-3'
