# Supplementary material for: Frontotemporal dementia associated CHCHD10V57E mutation aggravates tau pathology via disrupting the CHCHD10-Rab7A-TBC1D15 complex
Source: Cell Death Dis. 2026 Jun 19;17(1):762. doi: 10.1038/s41419-026-08983-9 (PMC13526787; doi:10.1038/s41419-026-08983-9)

Figure 2E

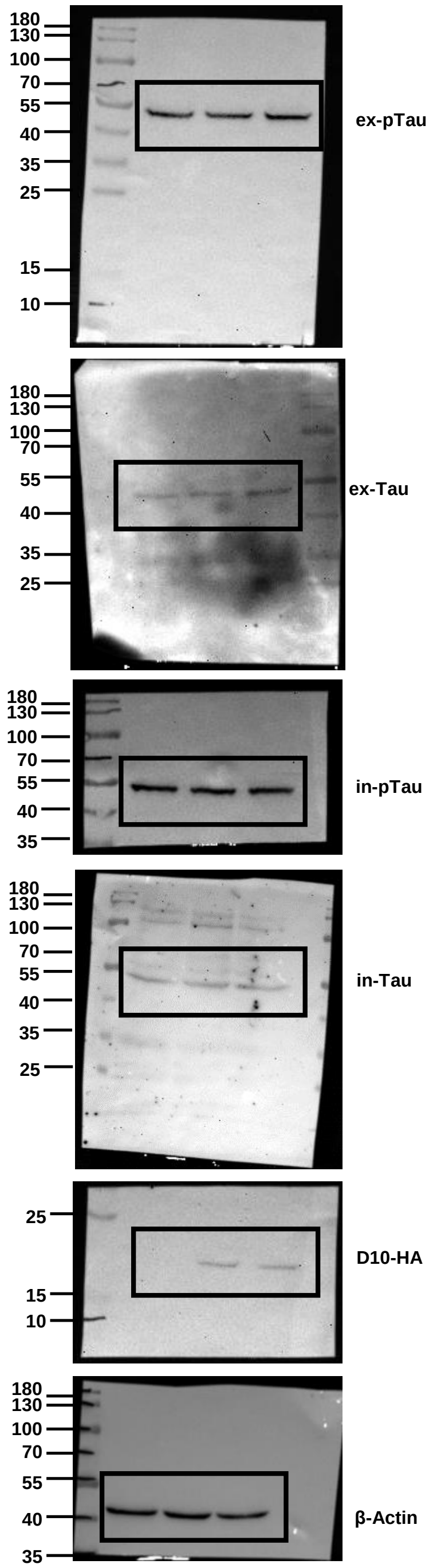

Figure 2J

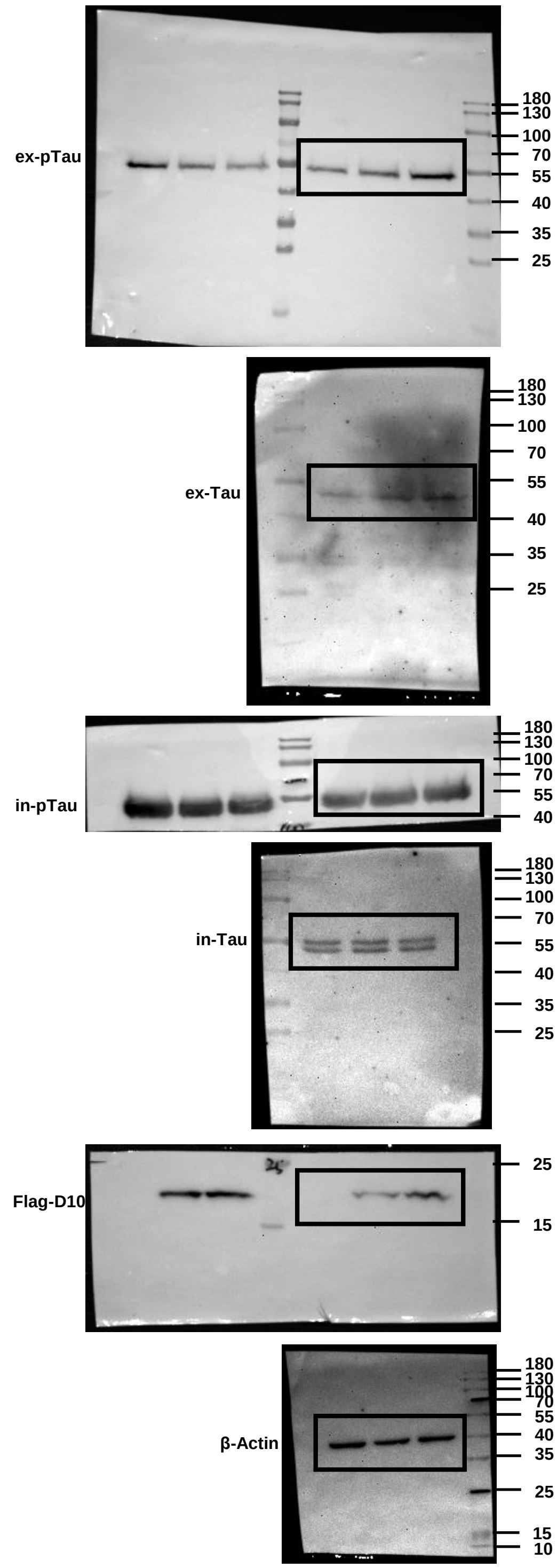

Figure 3G

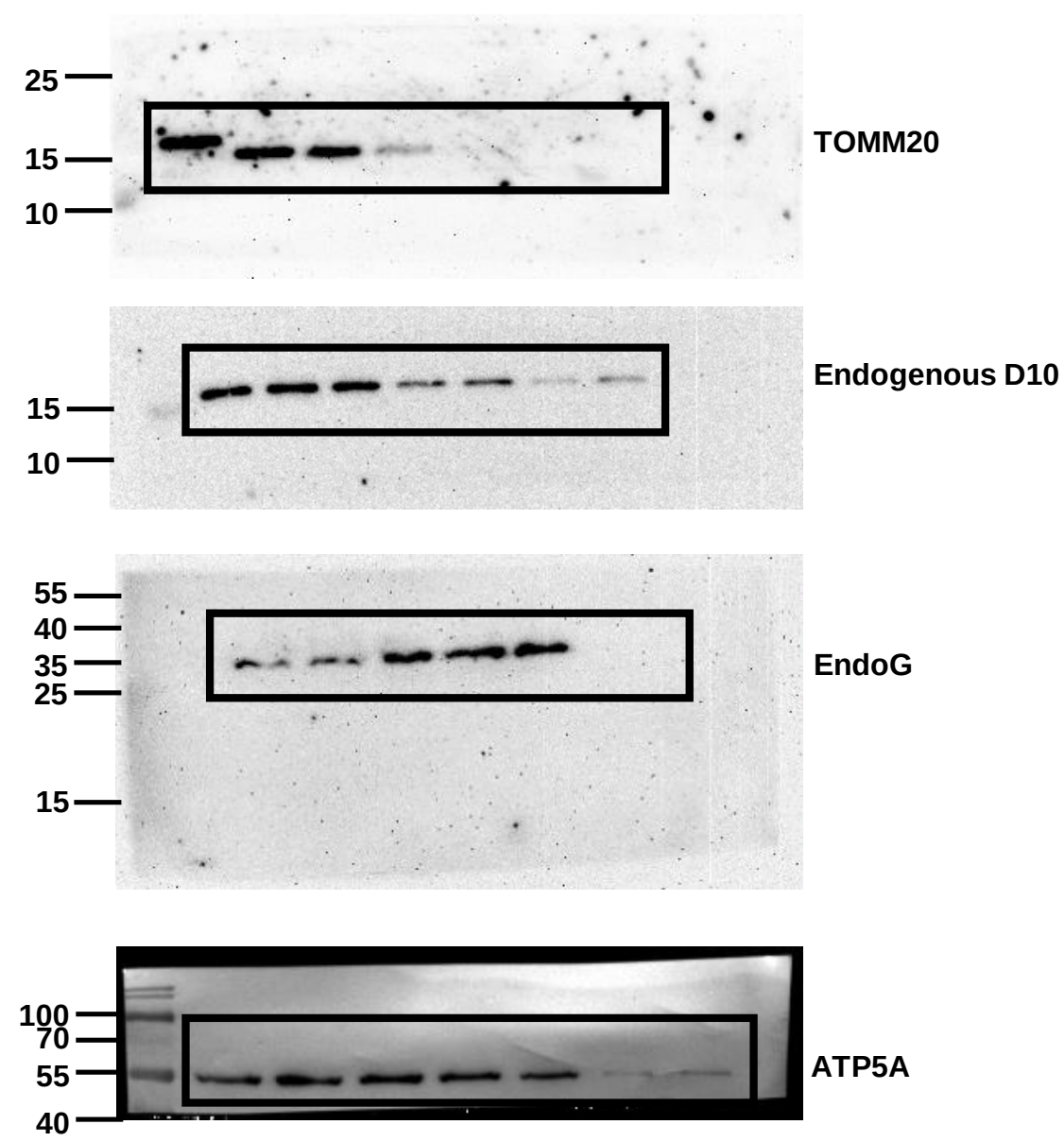

Figure 3H

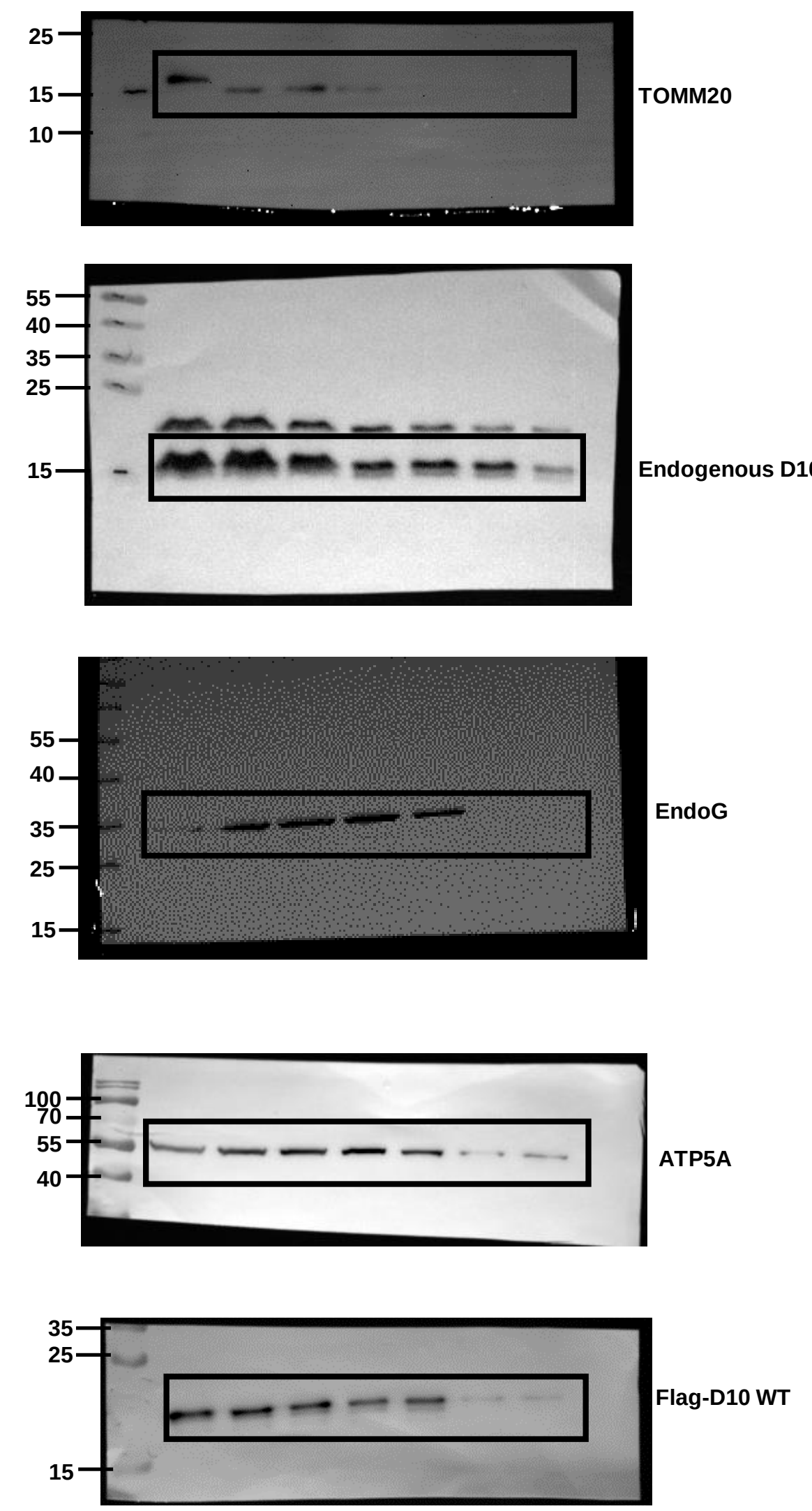

Figure 3I

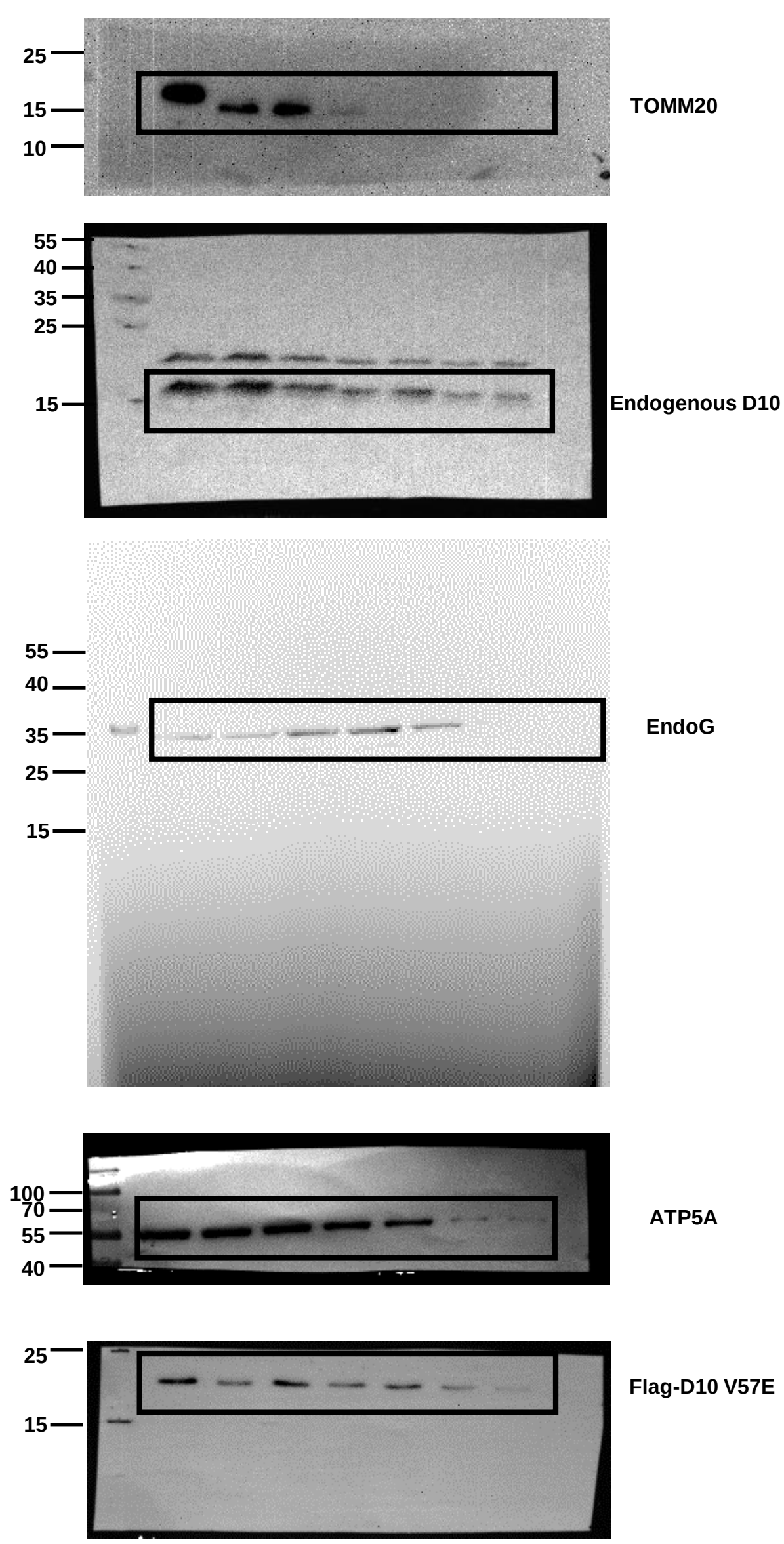

Figure 5J

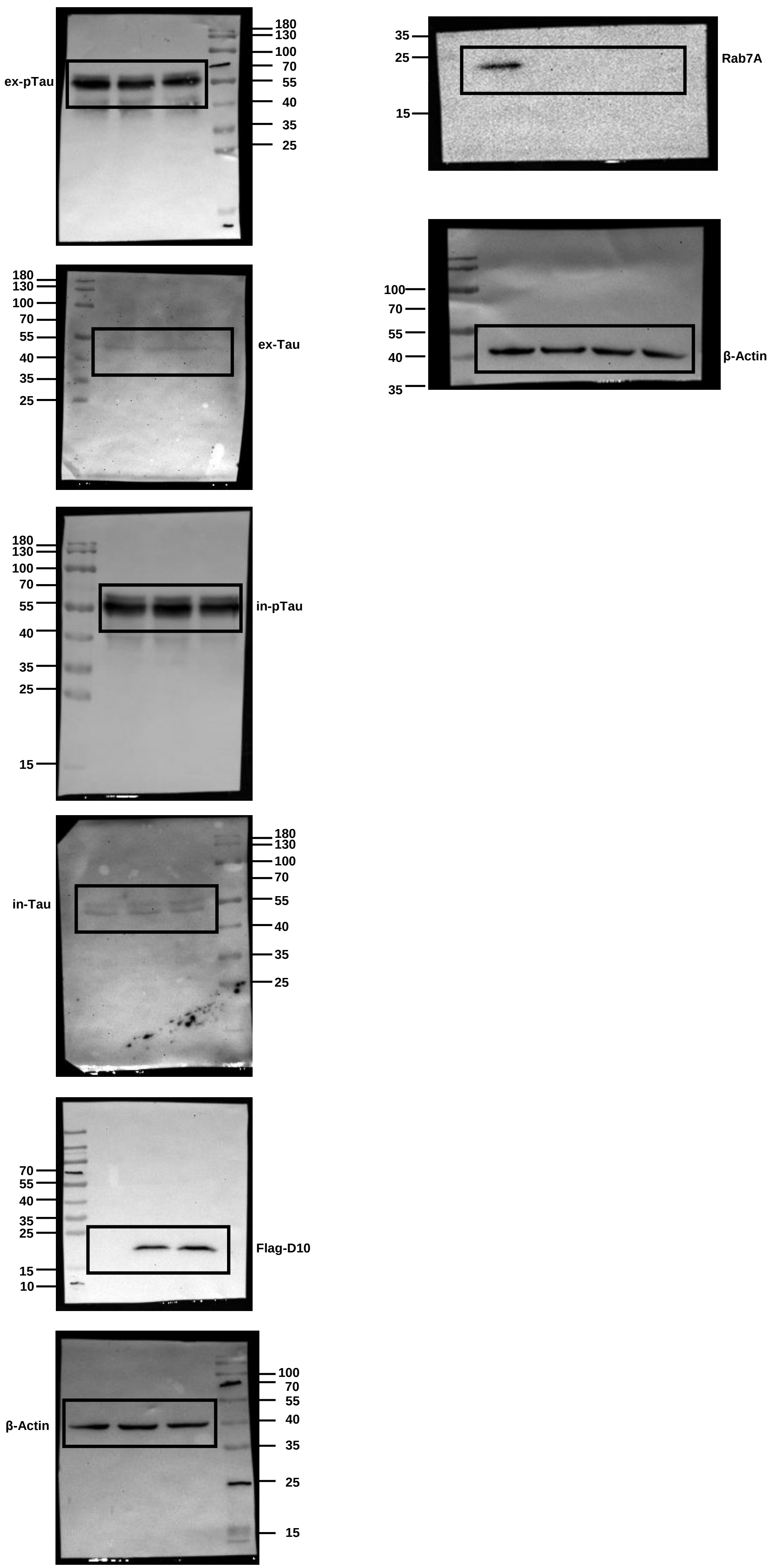

Figure 6K

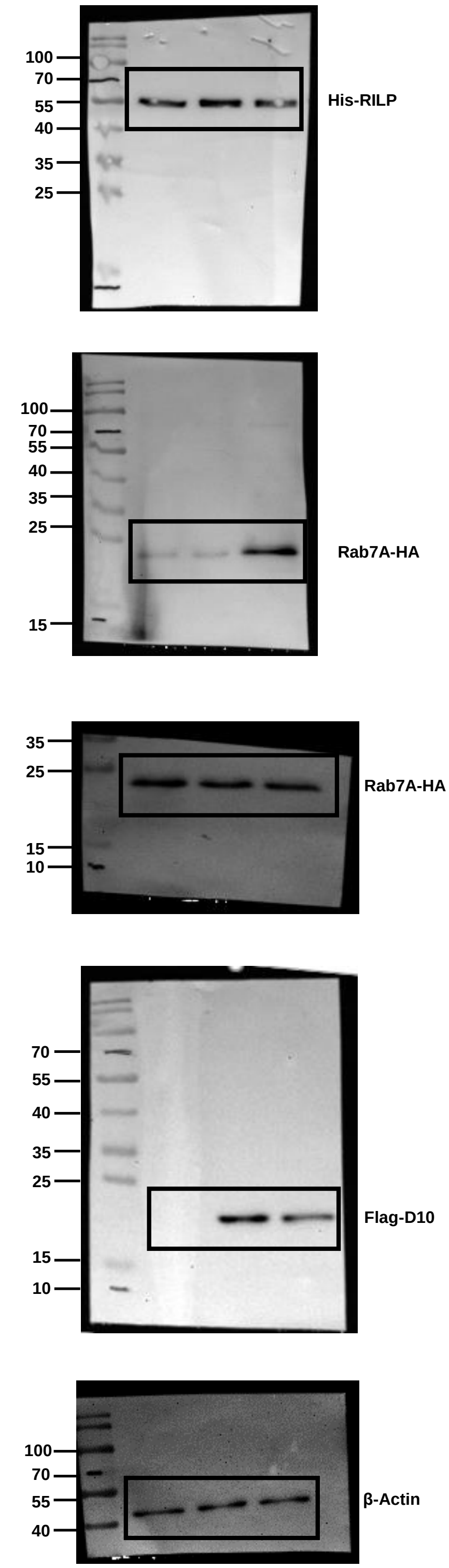

Supplementary Figure 2A

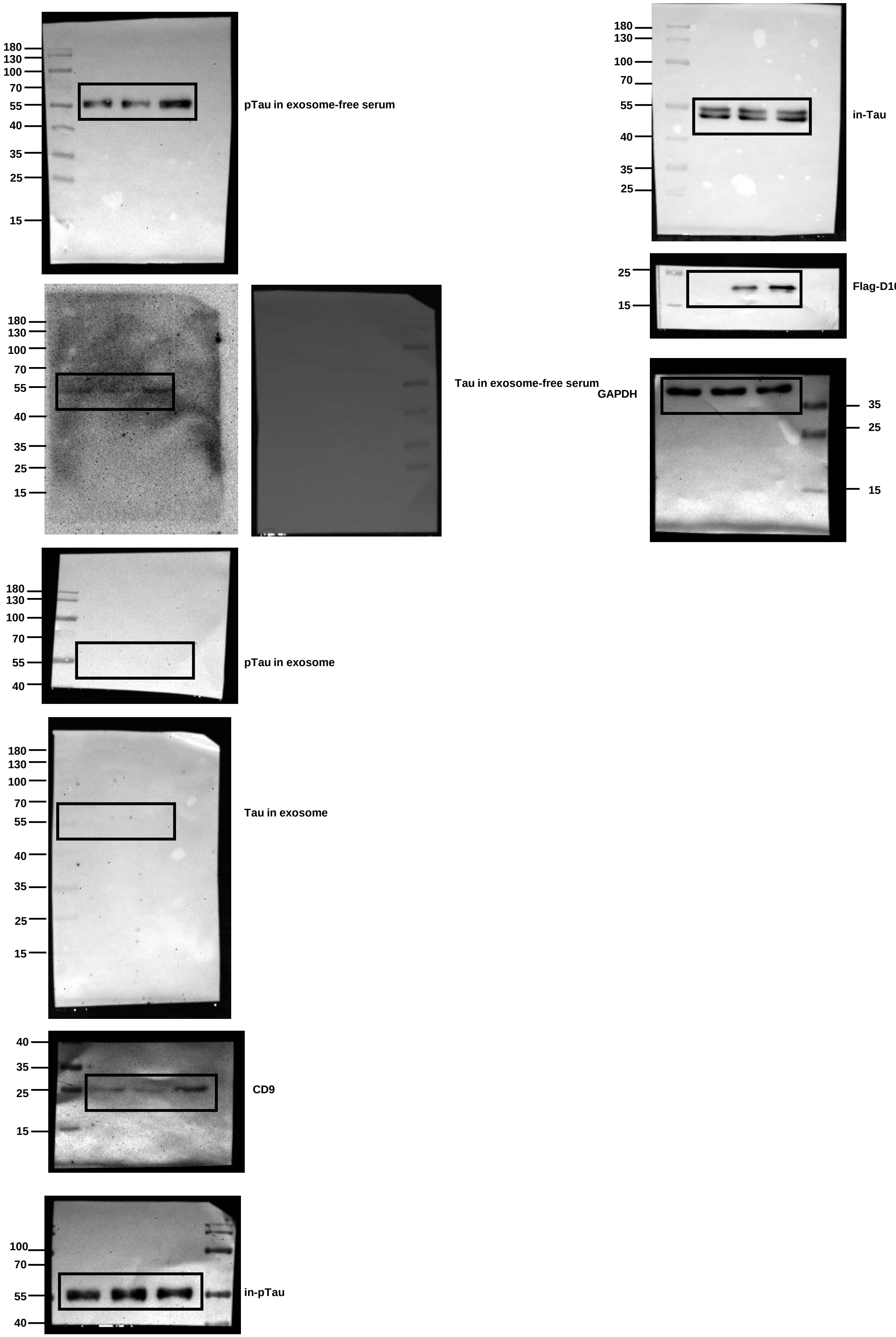

Supplementary Fig 7A

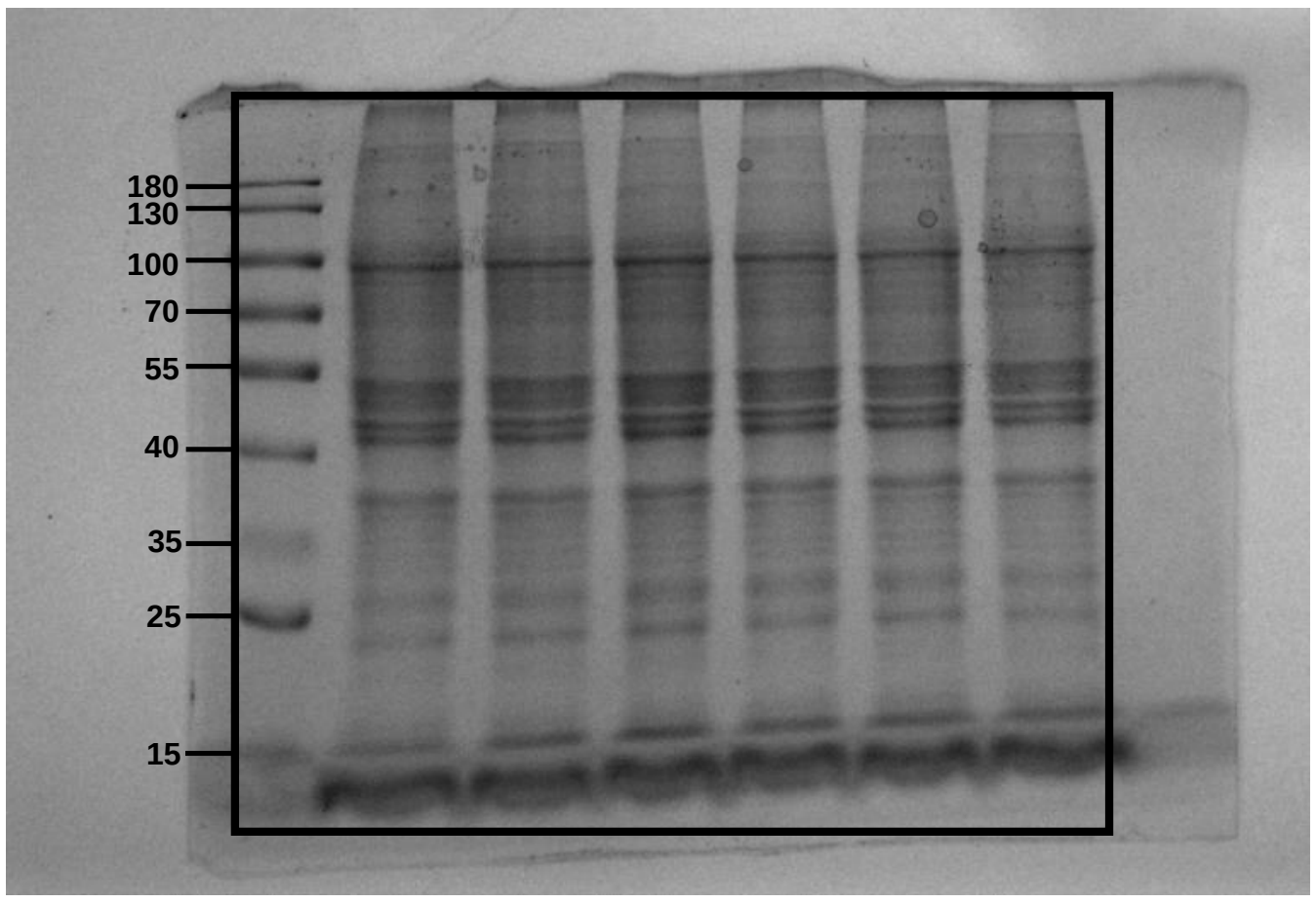

Supplementary Fig 6B

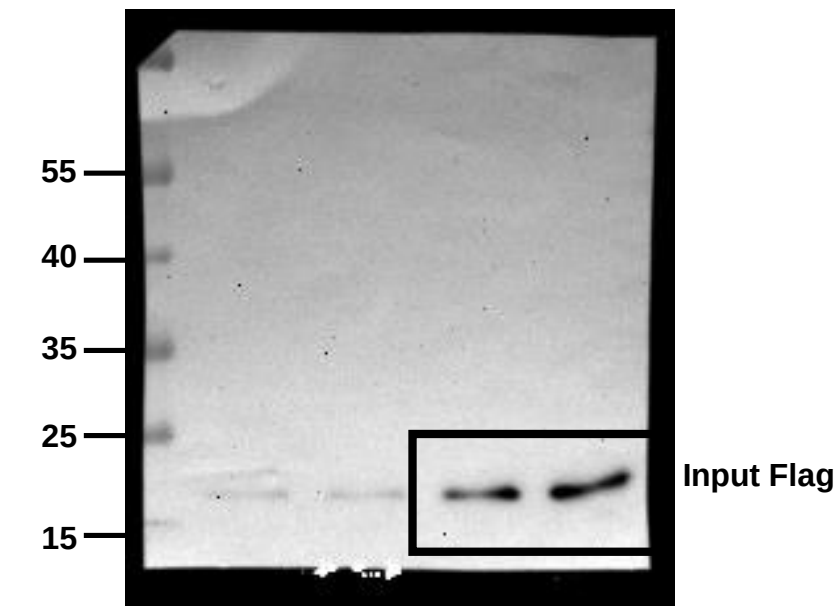

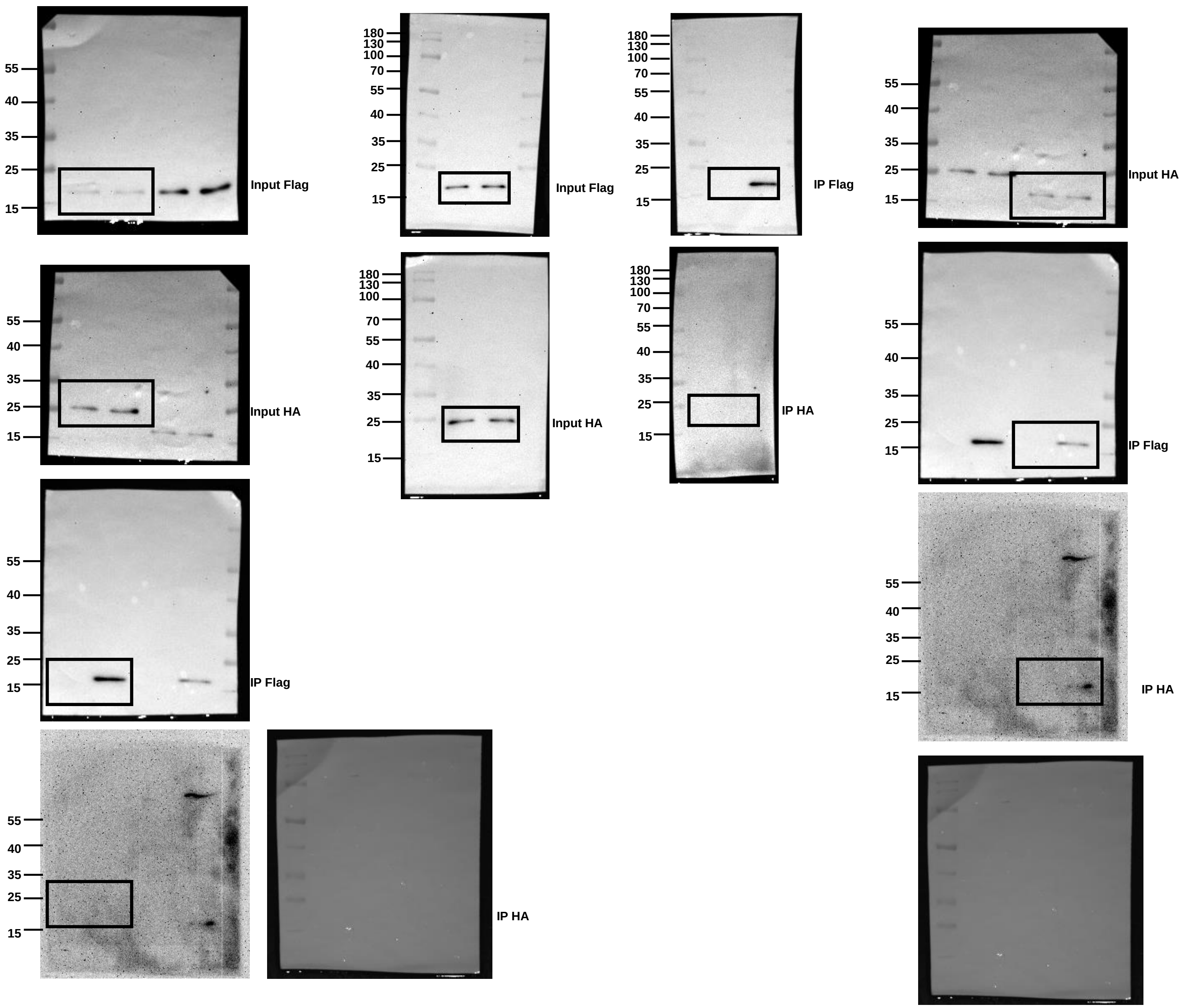

Supplementary Figure 10A

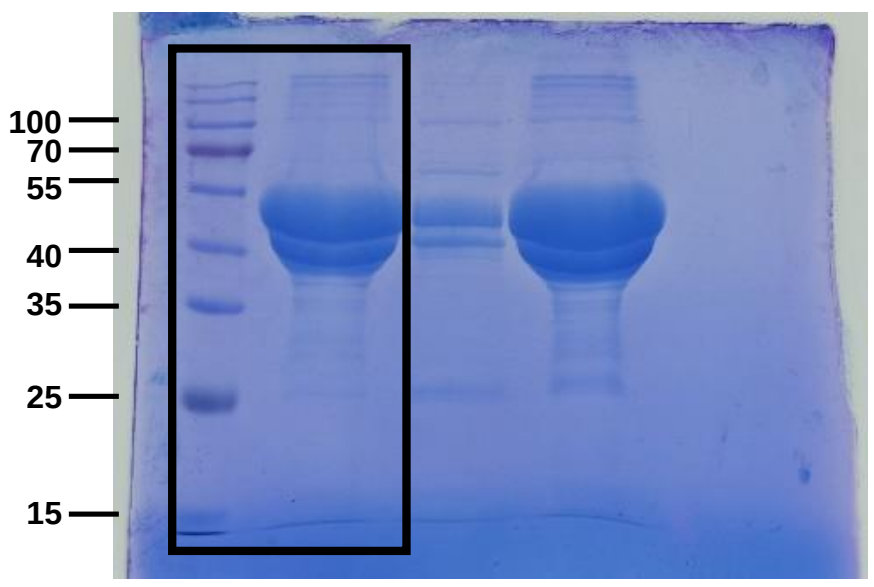

Supplementary Figure 10B

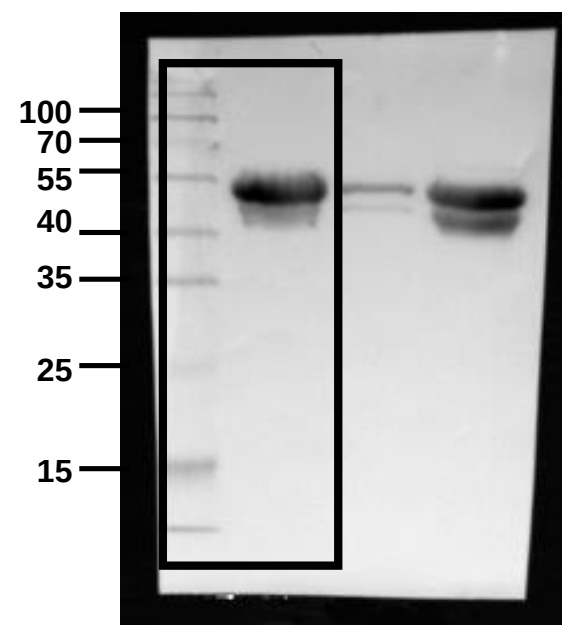

Supplementary Fig 12F

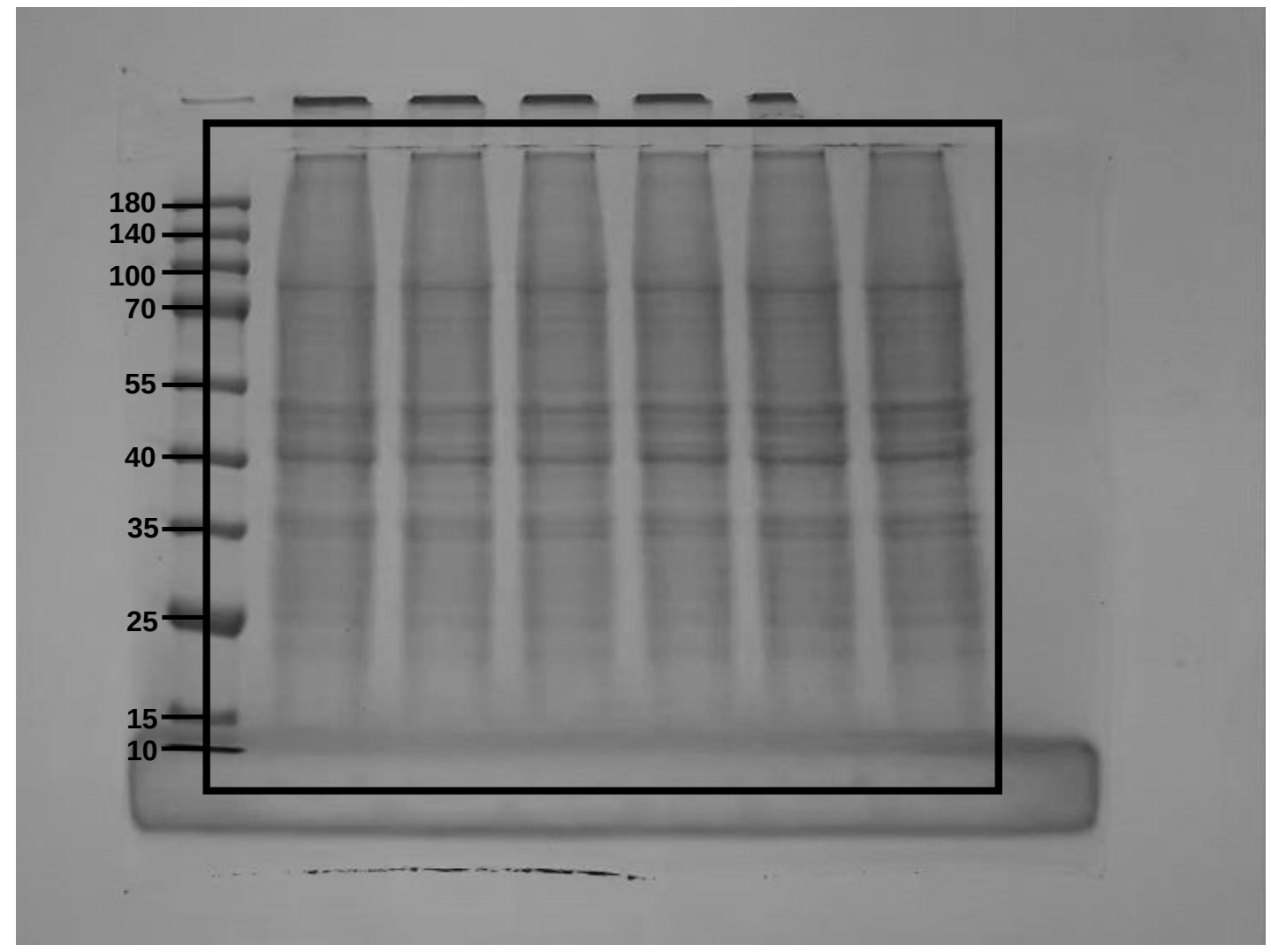

Supplementary Figure 12A

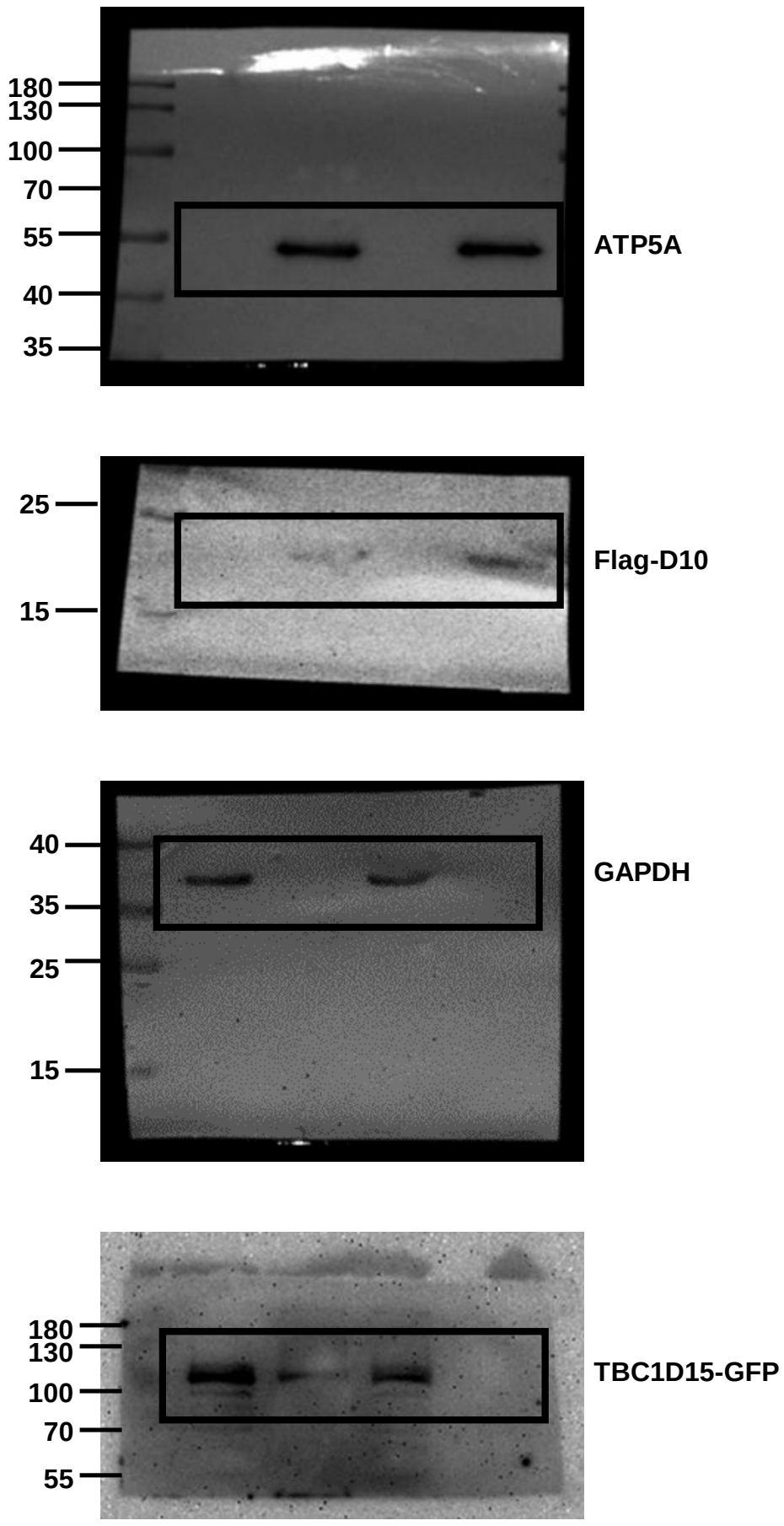

Supplementary Fig 12G

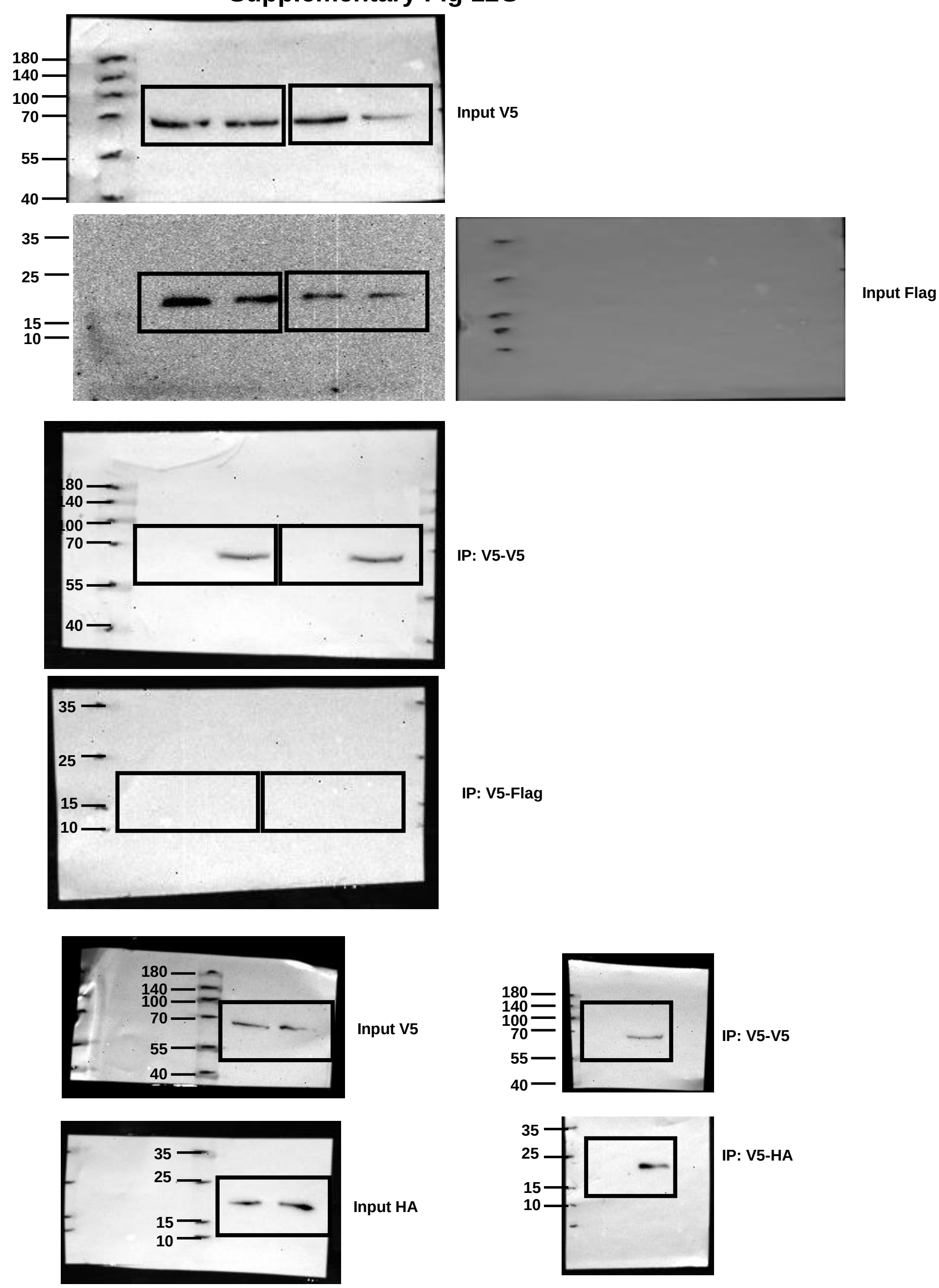

Supplement: Supplementary file 2 [file 41419_2026_8983_MOESM2_ESM.pdf]
